# Independent component analysis based gene co-expression network inference (ICAnet) to decipher functional modules for better single-cell clustering and batch integration

**DOI:** 10.1093/nar/gkab089

**Published:** 2021-02-22

**Authors:** Weixu Wang, Huanhuan Tan, Mingwan Sun, Yiqing Han, Wei Chen, Shengnu Qiu, Ke Zheng, Gang Wei, Ting Ni

**Affiliations:** State Key Laboratory of Genetic Engineering, Collaborative Innovation Center of Genetics and Development, Human Phenome Institute, Shanghai Engineering Research Center of Industrial Microorganisms, School of Life Sciences and Huashan Hospital, Fudan University, Shanghai, 200438, P.R. China; State Key Laboratory of Reproductive Medicine, Nanjing Medical University, Nanjing 211166, P.R. China; College of Life Science, South China Agricultural University, Guangzhou 510642, P.R. China; College of Agricultural, South China Agricultural University, Guangzhou 510642, P.R. China; State Key Laboratory of Genetic Engineering, Collaborative Innovation Center of Genetics and Development, Human Phenome Institute, Shanghai Engineering Research Center of Industrial Microorganisms, School of Life Sciences and Huashan Hospital, Fudan University, Shanghai, 200438, P.R. China; Division of Biosciences, Faculty of Life Sciences, University College London, London, WC1E 6BT, UK; State Key Laboratory of Reproductive Medicine, Nanjing Medical University, Nanjing 211166, P.R. China; State Key Laboratory of Genetic Engineering, Collaborative Innovation Center of Genetics and Development, Human Phenome Institute, Shanghai Engineering Research Center of Industrial Microorganisms, School of Life Sciences and Huashan Hospital, Fudan University, Shanghai, 200438, P.R. China; MOE Key Laboratory of Contemporary Anthropology, School of Life Sciences, Fudan University, Shanghai, 200438, P.R. China; State Key Laboratory of Genetic Engineering, Collaborative Innovation Center of Genetics and Development, Human Phenome Institute, Shanghai Engineering Research Center of Industrial Microorganisms, School of Life Sciences and Huashan Hospital, Fudan University, Shanghai, 200438, P.R. China

## Abstract

With the tremendous increase of publicly available single-cell RNA-sequencing (scRNA-seq) datasets, bioinformatics methods based on gene co-expression network are becoming efficient tools for analyzing scRNA-seq data, improving cell type prediction accuracy and in turn facilitating biological discovery. However, the current methods are mainly based on overall co-expression correlation and overlook co-expression that exists in only a subset of cells, thus fail to discover certain rare cell types and sensitive to batch effect. Here, we developed independent component analysis-based gene co-expression network inference (ICAnet) that decomposed scRNA-seq data into a series of independent gene expression components and inferred co-expression modules, which improved cell clustering and rare cell-type discovery. ICAnet showed efficient performance for cell clustering and batch integration using scRNA-seq datasets spanning multiple cells/tissues/donors/library types. It works stably on datasets produced by different library construction strategies and with different sequencing depths and cell numbers. We demonstrated the capability of ICAnet to discover rare cell types in multiple independent scRNA-seq datasets from different sources. Importantly, the identified modules activated in acute myeloid leukemia scRNA-seq datasets have the potential to serve as new diagnostic markers. Thus, ICAnet is a competitive tool for cell clustering and biological interpretations of single-cell RNA-seq data analysis.

## INTRODUCTION

With recent large-scale collaborative projects such as Human Cell Atlas, and technological advances such as droplet-based sequencing (1,2), single-cell RNA-sequencing (scRNA-seq) has become a useful tool for understanding cell fate decisions in human organs containing heterogeneous cell populations. Various bioinformatics methods have been developed to analyze the increasing amount of single-cell transcriptome data. Clustering individual single cells into biologically relevant sub-populations is one of the key steps in scRNA-seq analyses (3). However, it is still challenging to discover and characterize novel cell types in complex tissues owing to stochastic gene expression variation from various sources, such as transcriptional bursting (4), dropout during sequencing library construction (5), batch effect (6) and other technical sources.

To fully reveal the heterogeneity of single-cell expression data, multiple clustering algorithms, such as Seurat (7), SC3 (8), pcaReduce (9) and SINCERA (10), have been developed. The shared strategy among these algorithms is to project data into a lower dimensional space and calculate cell-cell distances to cluster cells. However, these methods usually measure cell similarity based on individual genes, ignoring that pathways and/or gene expression networks (a set of interacted genes) could also play important functions in cell-state decisions. To overcome such shortcomings, some computational methods integrate gene regulatory information to aid cell clustering and functional interpretations of scRNA-seq data. For example, SCENIC (11) integrates gene co-expression information inferred from a random forest model and putative transcription factor (TF) binding sites to facilitate cell-type prediction and biological interpretation. In contrast to integrating the transcriptional expression networks, SCORE (12) uses known protein–protein interaction (PPI) to trim gene co-expression networks, resulting in more credible and biologically meaningful networks (12). These network-based single-cell clustering methods enable more accurate cell-type predictions and have revealed biological insights into diverse biological processes, such as development, immune responses and tumorigenesis (11,12).

Despite the significant contributions that these network-based cell-clustering methods have made to the study of single-cell transcriptomes, a comprehensive understanding of the complex nature of cell heterogeneity in real tissues/organs is still lacking. Most of the currently used methods infer gene-expression modules by calculating gene-gene correlations to reflect co-expression among all cells (13). However, gene expression regulation is highly context specific (14–16). Consequently, correlation-based methods potentially overlook local co-expression effects existing in only a subset of cells (13,17), which has been supported by the evidence that different cell types exhibited distinct gene co-expression structure (18). Therefore, the use of regular network-based clustering algorithms may lead to the omission of the gene co-expression patterns of certain cell types.

Matrix decomposition methods, like principal component analysis (PCA), non-negative matrix factorization (NMF) and singular value decomposition, provide complementary strategies for gene co-expression module detection (13). Because it does not require genes within a module to be co-expressed in all the samples, matrix decomposition methods capture local co-expressions and provide low-dimensional representations of data in terms of ‘components’ that are usually linearly uncorrelated (19,20). Of note, this linear de-correlation does not mean every component is independent at higher dimensions, which may result in the failure to map components to independent biological processes, thereby hampering the correct understanding of activated modules or pathways in different single-cell clusters (21).

Here, we introduced the concept of independent component analysis (ICA) to single-cell clustering and network analysis. ICA has been extensively applied to gene expression data since the age of microarray (22,23). ICA has also been used to predict gene co-expression modules in bulk RNA-seq data analysis (18,23,24). ICA models the expression level of each gene in a given sample as a linear weighted sum of several independent components, and thus, decomposes expression matrix into a number of independent components, each termed an ‘expression program’ (21). Genes with the greatest projected values in a component are those most strongly correlated with the processes associated with this component (25). The statistically independent nature of ICA makes it more informative in gene function discovery (22,25), superior to other methods, like weighted gene correlation network analysis, PCA and NMF (13,18,21,26,27). Therefore, using ICA to infer gene co-expression modules in single-cell transcriptome analysis has the following advantages: (i) ICA can uncover essential data structure through its linear representation of the statistically independent components; (ii) ICA is more beneficial for predicting gene co-expression module(s) associated with rare cell type(s) owing to its ability to capture local gene co-expression structure; and (iii) ICA can detect consensus expression patterns across scRNA-seq datasets through clustering independent components (expression programs) from datasets of diverse origins, including their library construction strategies, sequencing platforms, laboratories or individuals, and other possible affecting factors (25,28). These ICA properties are beneficial for identifying more biologically meaningful modules and integrative analysis of scRNA-seq datasets from diverse sources.

ICA has mainly been used for dimensionality reduction or trajectory inference in scRNA-seq analysis (29); however, it has not been used to infer functional gene modules. Most previous works did not consider the distribution of gene attribute values in the context of gene interaction networks (such as PPI and TF-gene network), consequently, they failed to improve expression module prediction by integrating known gene network information. A current network-based single-cell clustering algorithm has been highly successful by integrating TF-regulon network into scRNA-seq analysis (11), but other types of molecular networks have rarely been utilized. SCORE firstly incorporated PPI and gene-gene correlation coefficients to single-cell clustering analysis (12), however, its performance relies heavily on the quality (such as gene coverage) of the scRNA-seq dataset (12). Therefore, an efficient scRNA-seq analysis tool incorporating both ICA and PPI information will aid the functional gene-module prediction and improve cell clustering in single-cell transcriptome study.

In this study, we developed a computational method called ICAnet (independent components analysis-based network inference) to decipher functionally relevant gene co-expression modules for improving the performance of single-cell clustering and batch-effect correction in scRNA-seq data analysis. ICAnet used ICA to infer shared and specific expression patterns across different batches, and it also incorporated PPI network or TF-gene regulatory network to detect ‘activated’ sub-networks (or modules) across diverse datasets, resulting in a better cell-clustering performance compared with other algorithms. ICAnet is the first tool integrating ICA with PPI information in cell clustering and gene interaction module predictions from scRNA-seq data. ICAnet also has the ability to perform batch-effect correction, which is helpful in integrating scRNA-seq data from different sources. The accuracy, scalability, robustness and reproducibility of ICAnet were also validated using several public high-quality single-cell datasets. More intriguingly, ICAnet has the capacity to find novel rare cell types that have not been revealed by previous computational methods.

## MATERIALS AND METHODS

### ICAnet overview

ICAnet is a module-based single-cell RNA-seq analysis tool, designed for integration, clustering and network analysis. This tool integrates gene expression information and high-quality PPI network in a novel way to precisely recover the landscape of single-cell expression atlas. ICAnet consists of three main steps: (i) Gene expression matrix preprocessing and decomposition; (ii) Cross-batch expression programs clustering; and (iii) Walk-trap-based activated ‘sub-network’ (module) identification. The details of these major steps are described as below.

### Gene expression matrix preprocessing and decomposition

#### Gene expression normalization

ICAnet requires single-cell gene expression matrices as input, which are then normalized through a standard pre-processing step (log-normalization for all gene expression matrices using the size factor 10 000 per cell, log_2_CP10K). Users can also specify other types of gene expression quantification (e.g. TPM or RSEM) and normalization methods (e.g. *SCTransfrom*) before running the subsequent core steps of ICAnet.

#### Denoising gene expression matrix

In each dataset used for integration or clustering, ICAnet aimed to identify biological signals from gene expression matrices and to identify shared expression patterns. For different batches of datasets with different levels of data sparsity, the variability of the data sparsity will adversely affect comparisons of expression programs across different datasets, because part of data variation (signal) is driven by the data sparsity, not the actual biological signal (30). To diminish interference from data sparsity, ICAnet implemented two alternative strategies: (i) Computing top *K* variable genes for each batch according to the coefficient of variation for each gene, taking the intersection set of all sets of variable genes as the filtered gene set, and using their corresponding expression profile to perform ICAnet; (ii) Using a recently developed Python module (named *randomly*) based on random matrix theory to denoise the dataset (30), which works very efficiently in eliminating single-cell sparsity-driven signals (30). We used it to denoise the dataset at first to prevent the influence of data sparsity on the matrix decomposition of ICAnet. In this study, we only applied the denoising preprocessing step to the pancreatic islet scRNA-seq datasets to improve the batch effect correction performance of ICAnet, because these datasets were generated from different library types and each dataset had different degrees of data sparsity.

#### Biological signal extraction via independent component analysis

To identify the biological signals (expression programs) in the dataset, we used ICA to decompose gene expression matrices into gene expression programs. The number of expression programs is a very important parameter in ICAnet, thus we proposed an unsupervised method based on random matrix theory (31,32) to determine this parameter (see Supplementary Notes, Section 1 in the Supplementary Materials). Each dataset was centered before performing ICA for matrix decomposition. Two different implementations of ICA can be utilized by ICAnet. The first implementation is the joint approximate diagnalization of eigenmatrices (JADE) (33). The major advantage of JADE over other implementation solutions is that it is based on matrix computations involving matrix diagonalization, resulting in non-stochastic components. Other algorithms (e.g. FastICA) rely on an optimization procedure (e.g. starting points and optimization paths) (34), therefore, may yield variable results. The second implementation is based on the R package *MineICA* (25), which uses the same strategy as *Icasso*, to alleviate the stochastic problem when running FastICA (35) through iterative component clustering. In this study, we used JADE-based ICA to decompose gene expression matrices into independent components (source matrix), and the gene weights (importance) of each component have unit variance and zero means.

### Cross-batch expression programs grouping

#### Grouping expression programs across batches to find shared biological signals

One key feature of ICAnet is the grouping of independent components (or expression programs) across different datasets/batches. First, ICA was performed independently on each dataset/batch. Then, the independent components computed from two (or more) single-cell datasets were compared by computing Pearson's correlation coefficient between corresponding gene weights of selected genes (projection value > 2.5 standard deviations in the identified component). After grouping of the components from different datasets/batches, Partitioning Around Medoids (PAM) algorithm (36) with the average silhouette width was used to estimate the optimal number of expression patterns. Finally, the medoids were chosen as the ‘basal programs’ shared across batches for further network weighting.

### Activated ‘sub-network’ (module) identification

#### Construction of weighted PPI networks with basal programs shared across batches

In the following step, we combined PPI networks and expression programs to integrate their information. The PPI networks were obtained from the STRING database, a common and widely used PPI database (37). In this analysis, we used a threshold of a combined interaction score >600 to filter interactions, which is also a commonly used criterion for obtaining credible PPI networks (12,38).

Those genes that significantly contribute to each expression program have been defined previously as the ‘activated genes’, which are identified using a weight threshold of three or four standard deviations from the mean. Here, we constructed weighted PPI network to produce activated sub-networks (or modules), wherein the edge-weight density is significantly greater than the rest of the network. We used the same weight scheme that used previously in computational epigenome model research (39). Specifically, for each component, the absolute weight value of each gene was determined and defined as ICA statistic $(IC{A_g})$. Assuming genes *g* and *h* are connected in the PPI, we assigned the edge weight as the average of the individual node (or gene) statistics, i.e. ${w_{gh}} = \frac{1}{2}\ ( {IC{A_g} + IC{A_h}} )$. To avoid prohibitive computational expenditures, we only assigned the edge weights to the edges with endpoint ICA statistics that passed the weight threshold and zero was assigned to other edges. The weight threshold can be manually adjusted, and in this analysis, we set it as 2.5 standard deviations from the mean.

#### Random walk trapping to identify sub-networks in weighted PPIs

To rapidly and robustly identify dense connected and activated sub-networks, we used the random walk approach (40) to decipher all the possible sub-networks (modules). We performed random walks of different lengths using our ICA statistics-weighted PPI networks and detected modules by applying walk-trap algorithm on each random walk-based distance matrix. All the detected modules greater than three were saved and pooled together as module sets. We then applied the AUCell algorithm to the raw single-cell datasets to construct activated module–cell matrix that calculates the enrichment of each module in each cell as an area under the recovery curve (AUC) across the expression value-based rankings of all or some of the genes. The cell–module activity is summarized in a matrix (termed as module activity matrix) wherein columns represent single cells and rows represent the predicted modules.

### Evaluation of clustering performance

#### Adjusted Rand Index (ARI)

When cell labels and batch information are available, the ARI can be used to calculate the similarity between the ICAnet clustering result and the known cell or batch labels (see Supplementary Notes, Section 2 in the Supplementary Materials).

We calculated the batch and cell-type ARIs for all the tested methods to evaluate their batch-effect correction performance. In addition, a combined F1 score was obtained for each batch correction method by computing the harmonic mean of the ARI score, as follows:

$$\begin{equation*}F{1_{ARI}} = \frac{{2AR{I_{{\rm cell}{\rm{\ }}{\rm type}}}\left( {1 - AR{I_{{\rm batch}}}} \right)}}{{1 - AR{I_{{\rm batch}}} + AR{I_{{\rm cell}{\rm{\ }}{\rm type}}}}}.\end{equation*}$$

#### Inverse Simpson's Index (LISI)

We used a score metric, named as LISI, to measure local diversity based on local neighborhood distribution (See Supplementary Notes, Section 2 in the Supplementary Materials). This index represents the expected number of cells that need to be sampled before neighboring cells are drawn from the same batch. The greater the score, the stronger the local batch_ID (iLISI) or cell_type (cLISI) heterogeneity is.

To measure the data from mixed batches, we calculated the value of the Area Under the Cumulative Distribution Function Curve (AUCDF). For the lowest batch or cell type mixing after integration, most of the iLISI or cLISI values is close to the beginning value of the iLISI or cLISI distribution (close to 1); therefore, the AUCDF value tends to be large. For the ideal batch (or cell type) mixing after integration, most of the iLISI or cLISI value is close to the end of the distribution (close to the number of the batches/cell_types); therefore, the AUCDF value tends to be small. We calculated the AUCDF using the following formulae:

$$\begin{equation*}{\rm{\ }}AUCD{F_{cLISI}} = \int_{1}^{{{n_{{\rm cell}\ type}}}}{{CD{F_{cLISI}}\left( x \right)dx}},\end{equation*}$$

$$\begin{equation*}AUCD{F_{iLISI}} = \int_{1}^{{{n_{{\rm batch}}}}}{{CD{F_{iLISI}}\left( x \right)dx.}}\end{equation*}$$

The AUCDF of the iLISI distribution with a good integration tends to be small and the AUCDF of the cLISI distribution with a good integration tends to be large. Also, a metric considering batch mixing and cell-type purification on all cells simultaneously is required; therefore, we defined the F1 score based on LISI for each batch-effect correction method by computing the harmonic mean of AUCDF as follows:

$$\begin{equation*}F{1_{LISI}} = \frac{{2AUCD{F_{cLISI}}\left( {1 - AUCD{F_{iLISI}}} \right)}}{{1 - AUCD{F_{cLISI}} + AUCD{F_{cLISI}}}}.\end{equation*}$$

### Clustering methods for cell states identification

For the cell states identification benchmark task, several methods were systemically compared. Before running clustering methods, we used count per million to derive a normalized count matrix. For t-Distributed Stochastic Neighbor Embedding (t-SNE)+*k*-means, pcaReduce and SC3, we used a log-transformed dataset and adjusted the number of clusters to optimize the clustering performance, which was evaluated by the ARI_cell type_. For SINCERA, we used z-score normalized data for the clustering analysis, and we also adjusted the number of expected clusters to optimize the ARI_cell type_ values. For Seurat, we used the Seurat packages and processed related datasets in accordance with the tutorial (https://satijalab.org/seurat/v3.2/pbmc3k_tutorial.html). We then performed cell clustering multiple times using Louvain clustering with multi-level refinement algorithms on a shared-nearest-neighbor-based cell graph, during which we adjusted the parameter resolution for the maximal ARI_cell type_. Three module-based clustering methods, SCENIC, SCORE and ICAnet, were compared in this study. All these methods quantified module activity based on AUCell. We ran each method and used the same *aucMaxRank* parameters to derive a module-based activity matrix.

For each clustering method, we used two variable gene selection criteria: the Top 5000 genes with the largest coefficient of variation, and the whole gene set. We then performed the above variable gene selection steps separately to select the criterion that produced the best clustering performance. For each test dataset, we re-analyzed the identifying novel rare cell types using Louvain clustering with a multilevel refinement algorithm (7) on a shared-nearest-neighbor-based cell graph derived from the module activity matrix to infer cell expression state.

### Clustering methods for multi-batch datasets integration

In benchmarking different multi-batch integration methods, we used Louvain clustering with multilevel refinement algorithms on a shared-nearest-neighbor-based cell graph for each method, and adjusted the resolution parameter to obtain the optimal ARI_cell type_ value. We then calculated corresponding LISI, iLISI and ARI_batch_ values. Additionally, for methods that correct batch effects on the Uniform Manifold Approximation (UMAP) space but not on the gene expression or PCA space in our study [e.g. BBKNN(41)], we applied Hierarchical DBSCAN + UMAP to cluster cells, and adjusted the parameters *minPts* to optimize the cell-clustering performance for comparisons.

### Identification of cell type-specific activated modules

To identify activated modules for each cell type, we first identified cell type-associated modules using a receiver operating characteristic (ROC) curve analysis (7). For each gene, we evaluated a classifier that was built on that module alone, to distinguish a specific group of cells from other cells. An AUC value close to 1 indicates that this module is more specifically expressed in a specific cell group. We implemented the above analysis using the FindMarker function provided by Seurat (7), with AUC > 0.75 as a threshold to call cell type-associated modules. Then, among the cell type-associated modules, continuous module activity was converted into binary values using AUCell (11) and the Spearman's correlation coefficient between each cell type and the binarized module were calculated. The modules with Spearman's coefficient < 0.3 were filtered out. Finally, the resulting modules with statistical significances greater than the threshold (*P*-value < 0.05, see Supplementary Notes, Section 5 in the Supplementary Materials) were selected and defined as cell type-specific activated modules.

### Stability and robustness evaluation of three module-based clustering algorithms

To test the stability of three module-based clustering algorithms [ICAnet, SCENIC (11) and SCORE (12)], we performed two different tests: (i) down-sampling the datasets with varied cell numbers (2000, 1000, 500 and 100); and (ii) simulation of low-sequencing depth by reducing the expression level to one-fifth of the original. We used the same down-sampling and gene expression simulation procedures for all the three tested methods, and the tSNE+DBSCAN clustering algorithm was performed to evaluate the newly predicted clusters. Finally, we calculated the ARI between the labels of identified clusters and previously annotated cell-type labels. In the clustering step, we ran DBSCAN multiple times, during which we altered the parameter *epsilon* in the range of 1.0–4.0 and *minPts* in the range of 1–50 to determine a maximal ARI.

### Module recovery analysis

Both SCORE and ICAnet intend to infer heavy sub-networks (modules) with average weight density values significantly larger than the rest of the network. The only difference is that SCORE defines the weights using gene co-expression coefficients, while ICAnet is based on ‘ICA statistics’. A well-inferred sub-network needs to be preserved or consistent across different datasets from the same tissue. We used the Monte Carlo randomization algorithm to measure the reproducibility of the oligodendrocytes-associated modules. First, we used the dataset by Zeiel *et al.* (42) to infer ‘SCORE’ and ‘ICAnet’ modules. Based on the module activity level, a cell type-association module for each cell type was first identified. Furthermore, we used the dataset by Marques *et al.* (43) to create new SCORE- and ICAnet-weight PPI networks, and tested whether the oligodendrocyte-associated module inferred from the dataset by Zeiel *et al.* could be reproduced in the dataset by Marques *et al.* (43). To compare the two algorithms, we assumed that they were based on the identical PPI topology. Therefore, we reassigned zero-weighted raw PPI edges with the smallest positive non-zero values (typically this value is close to zero, i.e. 0.001). Then, we permuted (1000 permutations) the edge weights around the network and recomputed modularities for the previously inferred oligodendrocytes-associated modules. Here, we defined modularity as the average weight of the modules. Finally, we computed the empirical module recovery score for each inferred oligodendrocytes associated module as follows:

$$\begin{eqnarray*}&&{\rm ModuleRecoveryScore} \nonumber\\ &&\quad = \frac{{{\rm num}\left( {{\rm Modularit{y_{permutation}}} > {\rm Modularit{y_{inferred}}}} \right)}}{{100}}.\end{eqnarray*}$$

For the ICAnet-weighted PPI network, a *K* number of different weighted PPI networks were determined. An over estimation of the number of weighted PPI networks results in some false positives during module recovery; therefore, we only computed the first independent component and created corresponding weighted PPI networks for the downstream analysis.

### Label-association analysis using graph signal processing

To identify which is the novel cell type (or state) among our cell-type labeling results, the intrinsic ‘label association’ between our cell-type annotations and those defined by the original author need to be determined. Inspired by a recently proposed signal-enhancing model (44), we used graph signal smoothing to transform the binary ‘cell-type label signal’ into a continuous ‘cell-type label signal’ to enhance label association.

For each cell type (denoted as *i*), we initialized a binary vector ${X_i}$ defined as follows:

$$\begin{equation*}{\rm{\ }}{\left( {{X_i}} \right)_k} = \left\{ {\begin{array}{@{}*{1}{c}@{}} {1\ {\rm if}\ {\rm cell}\ k\ {\rm is\ a\ member\ of\ cell\ type}\ i}\\ {0\ {\rm if}\ cell\ k\ {\rm is\ not\ a\ member\ of\ cell\ type}\ i} \end{array}} \right.\end{equation*}$$

To recover the latent continuous signal from the raw label, we used Laplacian regularization combined with the L2 norm loss function to reconstruct the signal, as follows:

$$\begin{equation*}{\rm{y\ }} = {\rm argmi{n}_z}\ \left\| {x - z} \right\|_2^2 + \beta {z^T}Lz,\end{equation*}$$

where *L* represents the Laplacian matrix of the cell–cell adjacency graph. We used a *k*-nearest neighbor graph with *k* = 30 to calculate the Laplacian matrix. The analytic solution for the above optimization issue is as follows:

$$\begin{equation*}{\rm{y\ }} = {\left( {I + \beta L} \right)^{ - 1}}{\rm{\ }}x.\end{equation*}$$

Therefore, the *y* is the reconstructed continuous signal vector for cell type *i*. We applied graph smoothing to each cell type to derive their continuous signal vector, and calculated the Pearson's correlation matrix between our annotated cell types and those in the raw cell-type annotations. *β* was assigned a value of 0.8 in this step. Furthermore, we used cor’ = (1+cor)/2 to transform the correlation matrix, and used 0.6 (Pearson's correlation coefficient > 0.2) and the FDR (false discovery rate) < 0.05 as thresholds to identify significant associations.

### Gene set enrichment analysis

We used the software GSEA (version 4.1.0), a Java desktop application to assess potential enrichment of specific gene sets in a ranked list of differentially expressed genes for each cell type. The curated gene sets are consistent of cancer stemness/risk associated gene-sets (45) and AML risk-gene *BAALC* expression associated gene-set (46).

### Survival analysis of acute myeloid leukemia (AML) patient based on module activity

To measure the activity levels of modules inferred from the scRNA-seq datasets in bulk RNA expression datasets, we first used gene set variation analysis (GSVA) (47) to calculate the module activity in each bulk sample. After converting the gene expression matrix into a module activity matrix, we selected the best subset of modules to predict survival in the training cohort. We used a linear regression model named Least Absolute Shrinkage and Selection Operator (LASSO) implemented by the *glmnet* R package (48). By enabling a 10-fold cross-validation to fit a Cox regression model, we were able to identify an optimized set of modules to predict survival. Owing to the randomness of the LASSO model, we applied a bootstrapping strategy to score each module. This procedure generated 100 resampled datasets from the complete sample sets, with a sample size equal to 80% of the whole samples. LASSO was performed with 10-fold cross validation to optimize the parameters for module selection in each resampled dataset. Finally, we scored each module based on how frequent this module was selected by the regression model during bootstrapping. On the basis of the resulting scores, we selected the top-*K* modules and performed PAM clustering on the samples guided by the selected feature modules to predict patient survival. We used the Top30 modules as AML patient-associated modules, because they yielded the most significant patient survival difference in the training dataset.

## RESULTS

### The principle and workflow of ICAnet

**Figure 1.**
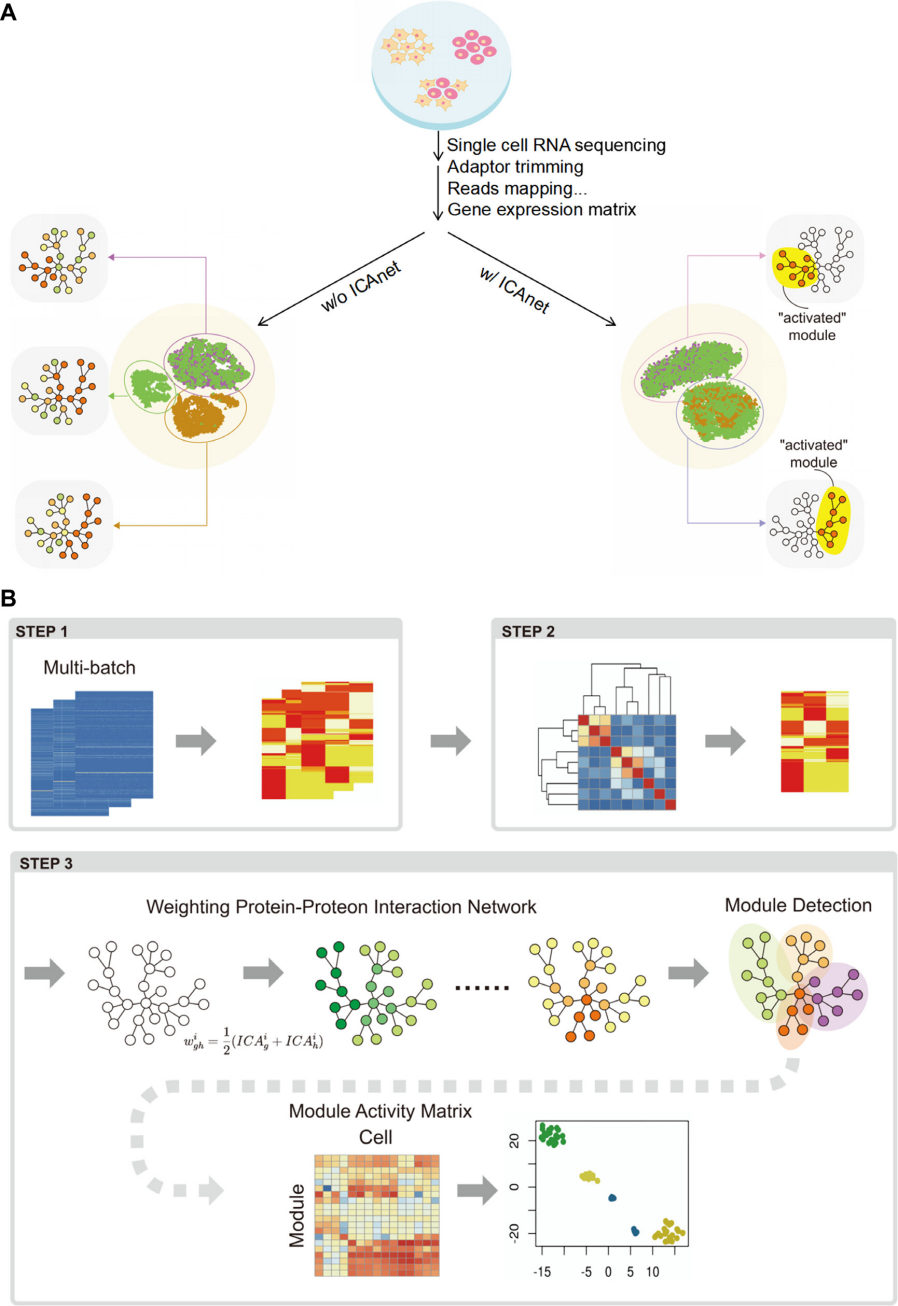
The principle and workflow of ICAnet. (**A**) ICAnet corrects batch effects via identifying shared gene co-expression module across different batches of data. Cell clustering on multi-batch dataset without ICAnet (left) cannot effectively separate cells of the same type (round or triangle dots in the central circle) coming from different batches (represented by different colors, i.e. brown, green and purple here). For example, purple and green batches on the up half (cell type A) have same gene co-expression network (purple arrow directed) while brown and green batches at the bottom (cell type B) have slightly different co-expression network (brown and green arrows directed). The area labeled with gray stands for gene co-expression network, within which colored circles and lines represent genes and their interactions, respectively. Red circle means these genes were detected to have co-expression in corresponding dataset. After correction by ICAnet (right), as it can identify shared gene co-expression module (exemplified by the ‘activated’ module highlighted in yellow area), these two clusters of cells were grouped together and regarded as the same cell type, consistent with the fact that they were derived from the same type of cells but subjected to different batch treatment, such as library type, sequencing platform and constructed by different labs or individuals. t-SNE visualization was used for cell clustering exhibition. (**B**) The workflow of ICAnet consists three main steps. In step 1, ICAnet applies ICA on each batch of data. In step 2, ICAnet grouped components from different batches together through Partition Around Medoids clustering algorithm (49). The average silhouette width is used to estimate the optimal cluster number, which acted as basal components for further analysis. In step 3, these components were used to incorporate PPI network information based on contribution of genes to each component. Each component combined with the PPI to generate a weighted PPI, followed by random walk with trapping to decompose each network to detect activated module. Finally, each module is scored within each single cell through AUCell algorithm (11). The resulted module-cell matrix was then used to perform downstream clustering analysis.

We introduced ICA (34) into single-cell clustering by decomposing a gene expression matrix into a number of independent components. Each component was characterized by a co-expression pattern and associated with certain meaningful biological pathways. Such concept enables ICAnet to identify shared gene co-expression module(s) across datasets from different batches (Figure 1A). Different batches of scRNA-seq datasets derived from the same cell type may not have exactly the same gene expression patterns but the key co-expression modules tend to be consistent. ICAnet pairs the same sub-population of cells among different batches, regardless of their library type, sequencing platform or other influences. These features of ICAnet make it perform well in cell clustering and integrative analysis on scRNA-seq datasets from different batches.

ICAnet takes a matrix of log_2_-transformed normalized gene expression value acquired by regular scRNA-seq analysis methods as input. For scRNA-seq data from multiple batches, ICA was used to decompose the gene expression matrix of each batch into a number of independent components. Each component was termed as an ‘expression program’, which was latently associated with certain transcriptional regulatory networks. To diminish the noise across batches (or batch-effect), ICAnet adopted an algorithm called PAM (49) to cluster all independent components, and the resulting clusters were defined as ‘basal programs’ (denoted by its medoid defined by PAM), which represented gene expression programs shared by (or similar among) different batches. However, these ‘basal programs’ may not necessarily represent real expression programs, they can also come from technical noise. Genes having protein products that interact with each other tend to have similar functions and co-expression patterns (16). To determine the genuine co-expression pattern shared among batches (or the featured expression module characterizing a give cell/tissue type), we incorporated PPI network information into ICAnet, and used the ‘basal programs’ to score PPI. The PPI sub-networks with high scores represented activated sub-networks (or gene-expression modules), which were inferred by a graph clustering algorithm named random walk with trapping (40). These resulting activated gene-expression modules minimized the influence coming from batch effect and represented the real biological signals shared across batches. Finally, ICAnet scored each cell based on the activated modules using the AUCell algorithm (11) and constructed a module-cell matrix for further analysis (such as cell clustering). The workflow of ICAnet is illustrated in Figure 1B and detailed in the Materials and Methods. In addition, to extend the usability of ICAnet to other types of molecular interaction networks, such as TF-target networks used by other tools (e.g. SCENIC), we also provided another version of ICAnet (called ICAnetTF) to incorporate TF-target interaction networks into scRNA-seq data analysis (see Supplementary Notes, Section 6 in the Supplementary Materials).

### ICAnet improves cell clustering and batch integration of cell-line scRNA-seq datasets

**Figure 2.**
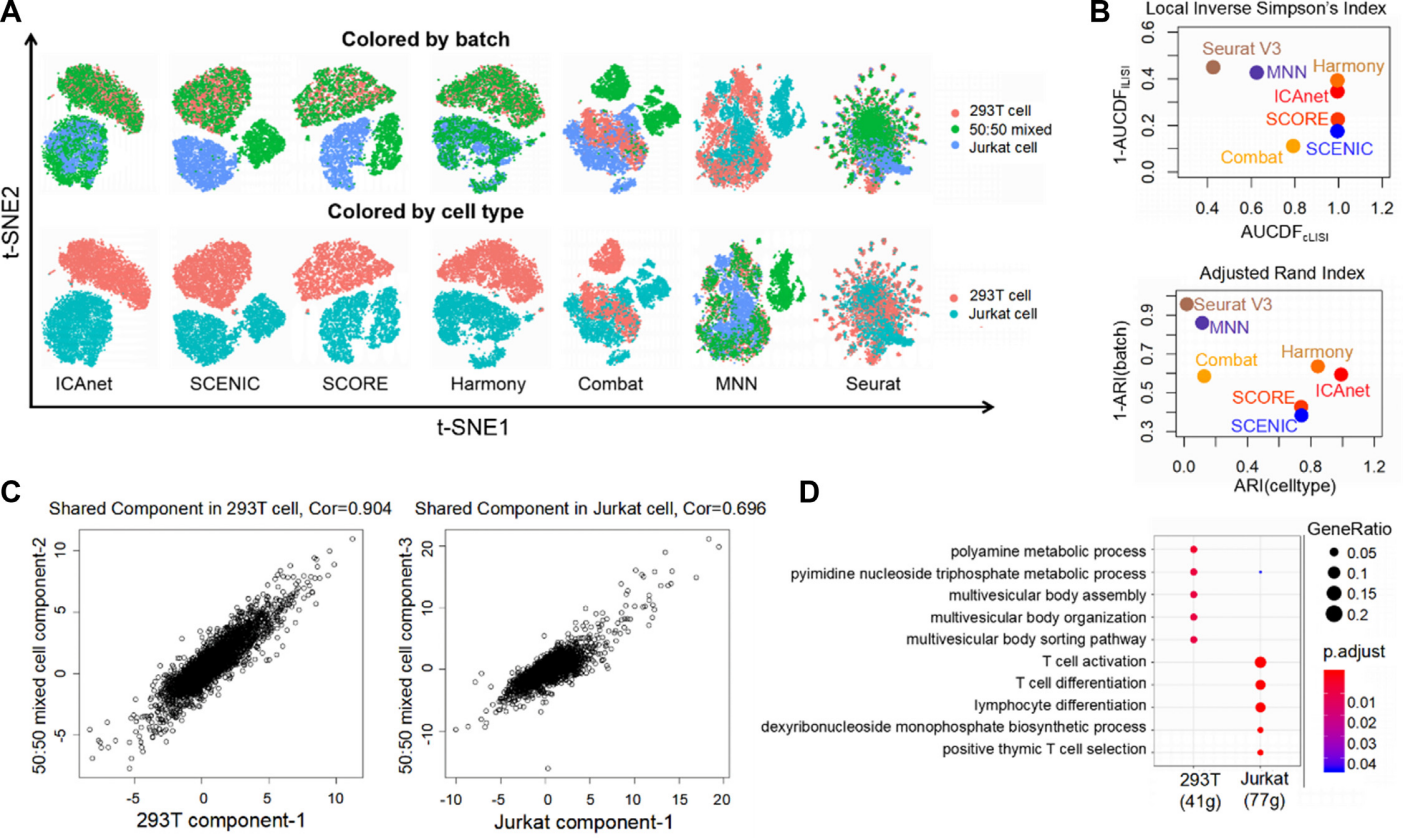
Performance assessment of seven batch-effect correction methods on cell line scRNA-seq data. (**A**) t-SNE visualization of ICAnet plus six other batch-effect correction approaches regarding batch label (pure 293T, pure Jurkat and 50:50 mixture) and cell type label (293T and Jurkat) provided by original authors. (**B**) Scatter plots to evaluate the performance of six batch-effect correction methods by AUCDF (top) and ARI (bottom). See ‘Materials and Methods’ section for details. (**C**) Scatter plots for independent components comparison between 50:50 mixed sample and pure cell line (left is pure 293T and right is pure Jurkat). Each point in the plots represents a gene, and Spearman correlation (Cor) is computed. *X* and *Y* axis represent attribute value of each genes on corresponding component. (**D**) GO enrichment analysis by clusterProfiler (104) for module genes belong to shared components indicated in panel (C).

To evaluate whether ICAnet can identify shared expression patterns among datasets from different batches to enhance both data integration and cell clustering, we first tested ICAnet on scRNA-seq datasets of known cell lines. Three scRNA-seq datasets [pure Jurkat cell (an immortalized human T-lymphocyte cell line), pure 293T cell (human embryonic kidney cell line) and a 50:50 mix of Jurkat and 293T cells generated by 10× Genomics were used for the analysis (Dataset DS1, see Supplementary Table S1). Both UMAP and t-SNE plots based on principal components (PCs) generated from the gene expression matrix indicated that strong batch effect existed (Supplementary Figure S1A–D). For a side-by-side comparison, these datasets were also analyzed using other algorithms, including SCENIC (11), SCORE (12), Harmony (50), fastMNN (51), Combat (52) and Seurat V3 (CCA) (7,53). ICAnet and Harmony clustered all the cells into two major groups in t-SNE and UMAP spaces, consistent with the fact that these datasets consisted of two cell types (Figure 2A and Supplementary Figure S1G). In addition, ICAnet performed a better batch-effect correction compared with other algorithms, even when integrating with other type of gene interaction networks (e.g. TF-gene) (Figure 2A; Supplementary Figure S1H and I).

Next, ARI and LISI were used to quantitatively evaluate the performances of these seven algorithms. The ARI score assesses the coincidence between predicted cell clusters and cell type/batch labels given by the original authors (ARI_cell type_ and ARI_batch_, respectively) (54), while LISI measures the local diversity of cell types (cLISI) or batches (iLISI) (50). Although Harmony performed a slightly better batch mixing compared with ICAnet (as indicated by higher AUCDF_iLISI_, Figure 2B, top panel; Supplementary Figure S1E and F), ICAnet outperformed Harmony in cell-type prediction accuracy [ARI_cell type_ (ICAnet) = 0.99, ARI_cell type_ (Harmony) = 0.84, Figure 2B, bottom panel; Supplementary Figure S1J and K]. SCENIC and SCORE grouped all the cells into three clusters (Figure 2A). The remaining three methods showed no clear clustering (Figure 2A). These above results suggest that ICAnet is a competitive method for batch correction and cell clustering in scRNA-seq analysis.

Because ICAnet separates independent components from a mix of single cells, a certain component from the 50:50 mixed cells could represent features from either 293T or Jurkat. To validate such an expectation, we extracted each independent component from the 50:50 mixed sample and compared them with each of the cell types. The second and third components of the 50:50 mixed dataset were highly correlated with the top components of the Jurkat and 293T cell lines, respectively (Figure 2C), supporting the idea that ICAnet can successfully separate expression programs from mixed data. To further confirm that shared gene co-expression modules (‘activated’ modules in Figure 1A) could reflect cell-type features, we performed Gene Ontology (GO) analysis on those shared activated genes belonging to the activated modules (Figure 2C). Interestingly, genes in activated modules that correlated with 293T or Jurkat cells showed distinct enriched GO terms (Figure 2D). While the 293T-associated gene module was enriched in metabolic pathways such as polyamine metabolic process and UTP/GTP biosynthesis (55), the Jurkat-associated gene module was enriched more in T cell-related functional categories such as T-cell activation and differentiation, which was in line with Jurkat being an immortalized human T-lymphocyte cell line (56). These results indicate that ICAnet can find shared and specific expression programs across different batches, thereby increasing its batch correction and clustering efficiencies.

### ICAnet improves integration performance independent of library construction strategies

To demonstrate that ICAnet can be applied to more biologically relevant data with different types of batch effect, we analyzed two mouse hematopoietic cell datasets derived from different scRNA-seq library-construction approaches (Dataset DS2, see Supplementary Table S1). The first dataset is derived from a SMART-seq2 based scRNA-seq library preparation of hematopoietic stem and progenitor cell populations in 12-week-old female mice (57). The second dataset is derived from a MARS-seq library of myeloid progenitors from 6 to 8-week-old female mice (58). Each cell in both studies had been assigned a known cell type using fluorescence-activated cell sorting (57,58), facilitating the interpretation of the downstream analysis results. These two datasets mainly contained three shared cell types (including common myeloid progenitors (CMPs), granulocyte-monocyte progenitors (GMPs) and megakaryocyte-erythrocyte progenitors (MEPs), Supplementary Figure S2A) and were widely used for batch-effect correction evaluation of different methods (6). Since these two datasets have different sequencing depth, the direct concatenation of them to calculate the gene co-expression would result in false positive correlations for certain genes, such as *B2m* and *Xist* (Supplementary Figure S2B). An integrative analysis of such datasets requires a powerful computational method to correct the batch effect.

**Figure 3.**
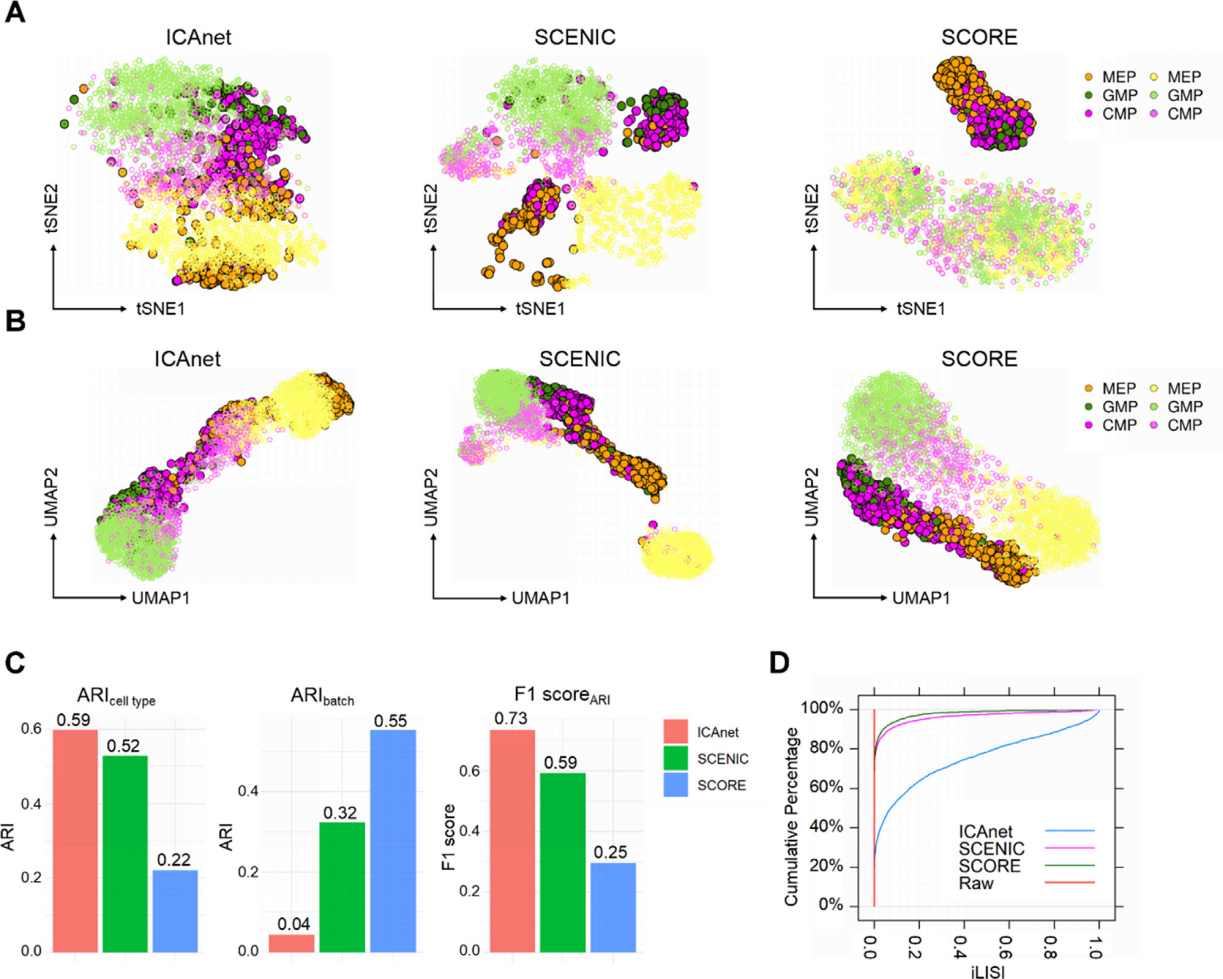
Performance comparison for three network-based methods between two library types of mouse hematopoietic cells. (**A** and **B**) t-SNE (A) and UMAP (B) visualization for three co-expression network-based clustering algorithms. Dark and light color pairs denote SMART-seq2 and MARS-seq, respectively. (**C**) Assessment of cell type accuracy (left), batch correction efficiency (middle) and combined performance (right) reflected by ARI_cell type_, ARI_batch_ and F1 score_ARI_, respectively. A larger ARI_cell type_ value means better performance, while a smaller value ARI_batch_ denotes better batch effect correction. A larger F1 score_ARI_ reflects a better performance on both aspects. (**D**) Assessment of batch mixing through iLISI.

For a fair comparison, we extracted the expression profiles of the three shared cell types in these two datasets and compared the results of ICAnet with SCENIC and SCORE (both are module-based methods) to examine whether ICAnet performs better in cell clustering and batch-effect correction. Results were visualized using both t-SNE (Figure 3A and Supplementary Figure S2C) and UMAP (Figure 3B and Supplementary Figure S2D), and they revealed that before batch-effect correction, the cells largely grouped according to batch resources, while ICAnet grouped cells of the same cell type between the two batches more efficiently than the other two methods. We also validated that ICAnetTF clustered cell efficiently regardless of batch sources (Supplementary Figure S2E). A quantitative evaluation with ARI also indicated that ICAnet and ICAnetTF had better cell-type prediction accuracy than the other two network-based methods (Figure 3C and Supplementary Figure S2F). Besides, ICAnet and ICAnetTF performed better batch-effect correction, as reflected by the higher AUCDF_iLISI_ (batch mixing) value (see ‘Materials and Methods’ for details) compared with SCENIC and SCORE (Figure 3D and Supplementary Figure S2G). Visualization with t-SNE and UMAP also showed that ICAnet produced a more accurate differentiation trajectory than the other two methods (Figure 3A and B), as MEP and GMP are differentiated from CMP (59). We also performed a similar analysis including all cell types and arrived the same conclusion (Supplementary Figure S2H).

To further validate the batch-effect correction efficiency of ICAnet on scRNA-seq datasets from different sources, we also applied ICAnet to three scRNA-seq datasets of human pancreatic islet cells that had been prepared using different library construction strategies (Dataset DS5, see Supplementary Table S1; two were 3′-tag sequencing and one was full-length sequencing) (60–62). Integrating these datasets was particular challenging because each dataset had a number of unique co-expression structures. ICAnet largely removed the batch effect originating from both donor and library type (Supplementary Figure S3A and B) and grouped the cells from these three independent datasets according to the cell types annotated by their original authors. We also analyzed these datasets with other eight batch-effect correction methods for performance comparisons with ICAnet. A visual inspection showed that ICAnet and Seurat V3 (CCA) grouped the cells according to their cell types but not the batch source on both t-SNE and UMAP spaces, while some methods, like BBKNN (41) only showed batch-effect correction on the UMAP space but not the t-SNE space (Supplementary Figures S3 and 4). Additionally, SCENIC and SCORE showed poor batch effect correction in these complicated datasets (Supplementary Figures S3–5). To compare the performance of different methods, we used F1 score_ARI_ to simultaneously evaluate the performance of both cell-type inference and batch-effect correction. We also used the harmonic mean of AUCDF_cLISI_ and 1-AUCDF_iLISI_ (defined as F1 score_LISI_, see ‘Materials and Methods’ section for details) to evaluate cell-type purification and batch mixing. We found that ICAnet had good performance regarding the F1 scores of both ARI and LISI [top 1 in F1 score_ARI_ (0.905) and top 3 in F1 score_LISI_ (0.66); Supplementary Figure S5A and B]. In summary, ICAnet can surpass or is comparable with the most state-of-the-art methods for batch-effect correction of scRNA-seq data of various origins.

### ICAnet works stably in multiple datasets having different sequencing depth and cell numbers

**Figure 4.**
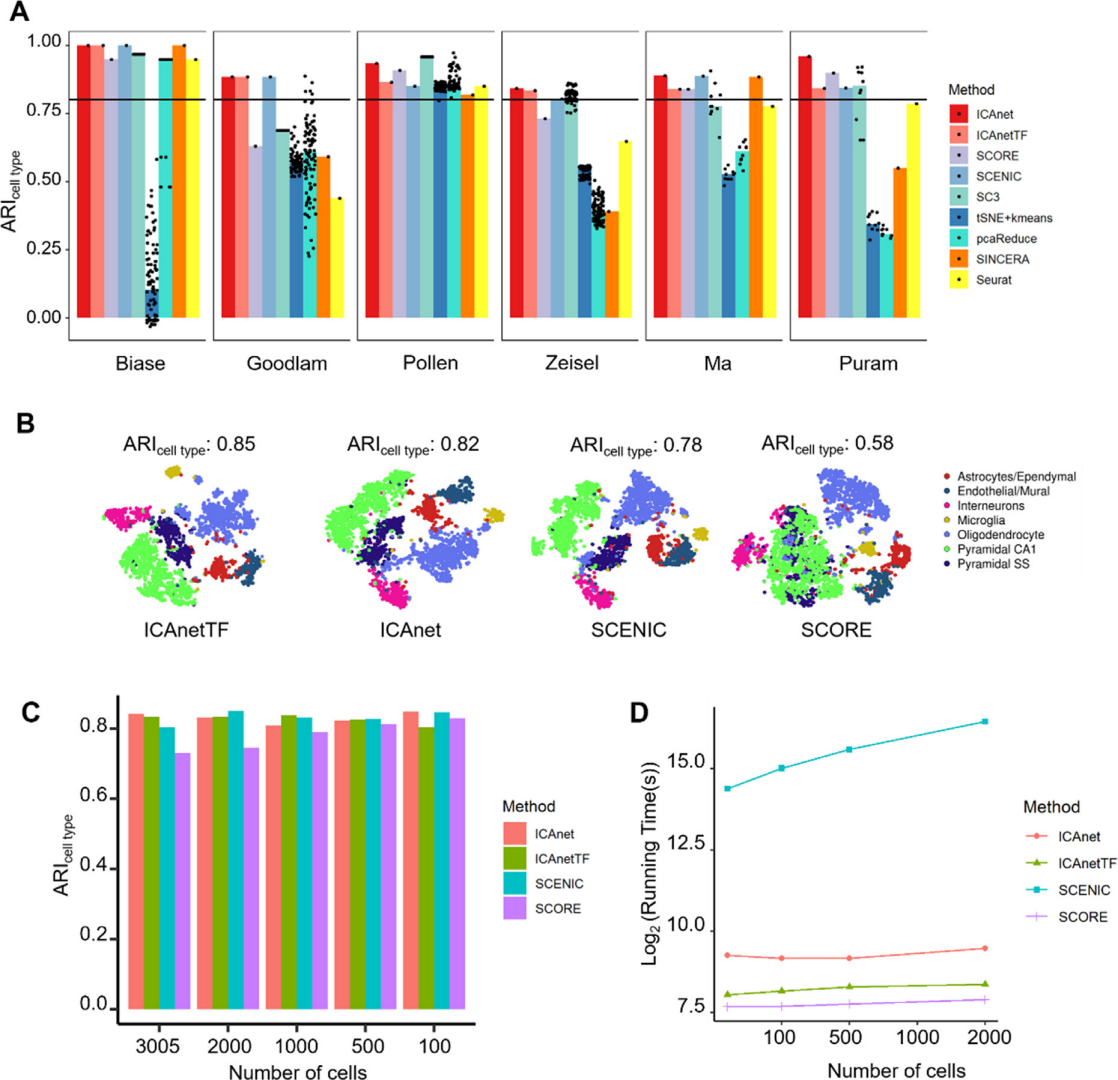
Performance comparison for different methods among multiple datasets with different read coverages and cell numbers. (**A**) Nine clustering methods were used for side-by-side ARI_cell type_ comparison in datasets with diverse sample sizes. The horizontal line denotes the position of 0.8 for ARI. The spots denote the rounds that each method ran (see ‘Materials and Methods’ section for details). The *X*-axis refers to the public datasets denoted by the name of first author. (**B**) t-SNE plots of four network-based methods on simulated dataset of low sequencing depth (3005 mouse brain cells). The calculation of clustering accuracy (represented by ARI_cell type_) here is based on DBSCAN clustering results on simulated datasets. (**C**) Clustering accuracy (ARI_cell type_) comparison of four network-based methods on simulated data with different sequencing cell numbers (mouse brain scRNA-seq data by Zeisel *et al.*). (**D**) Comparison of running time among these four network-based methods (data by Zeisel *et al.*). Y axis denotes the log2 transformed running time (second) and *X* axis represents the increasing cell numbers involved in simulated data.

To better evaluate the clustering performance of ICAnet, we compared ICAnet and ICAnetTF with seven other methods [SCENIC, SCORE, SC3, gene expression tSNE followed by *k*-means clustering (tSNE+*k*-means), pcaReduce, SINCERA and Seurat (7–10)] using six different scRNA-seq datasets (Dataset DS3–4, see Supplementary Table S1). Three of the datasets were of small sample size (< 2000 cells; Biase *et al.*, Goolam *et al.* and Pollen *et al.*) (63–65) and the rest three were of large sample size (> 3000 cells; Zeisel *et al.*, Ma *et al.* and Puram *et al.*) (42,66,67). The results showed that only ICAnet, ICAnetTF and SCENIC performed stably on all six datasets (ARI_cell type_ value > 0.8; Figure 4A), and ICAnet slightly preceded SCENIC in performance for three datasets (Figure 4A).

To further assess the impact of sequencing depth on the robustness of ICAnet, we simulated low-coverage data by reducing the coverage depth of each gene to one-fifth of the raw scRNA-seq data derived from mouse brain (one of the six datasets used above that has been widely used for benchmark studies) (42). The average number of detected genes per cell in simulated low-coverage data is 1240, while that of the raw data is 3713. We next ran ICAnet, ICAnetTF, SCENIC and SCORE simultaneously for cell-clustering comparison, and ICAnet and ICAnetTF still performed well on low-coverage datasets (having ARI_cell type_ values of 0.82 and 0.85, respectively; Figure 4B), better than SCENIC and SCORE (having ARI_cell type_ values of 0.78 and 0.58, respectively). These results suggest that ICAnet captures gene co-expression structure for better cell clustering even on low-coverage datasets. We also replaced ICA with other matrix decomposition algorithms (including PCA and NMF) to benchmark the influence of ICA on the clustering performance of ICAnet and found that ICA performed either better or comparable cell clustering than PCA and NMF (Supplementary Figure S6A). Additionally, we compared ICAnet with other ICA-based tools on single-cell clustering and found that ICAnet performed better than previously developed ICA-based transcriptome analysis tools on single-cell clustering (Supplementary Figure S6B).

The influence of sampling size (number of single cells sequenced) on cell clustering was next examined among ICAnet, ICAnetTF, SCENIC and SCORE. We evenly down-sampled the number of cells in the same mouse brain expression data used above (the original cell number is 3005) to 2000, 1000, 500 and 100 cells and then performed clustering on each sampled dataset using all these four methods. ICAnet consistently produced better ARI_cell type_ than SCORE, but was comparable to SCENIC on down-sampled datasets (Figure 4C). Of note, the running time of SCENIC was 30–130 times longer than ICAnet at different cell numbers, while the running times of ICAnet, ICAnetTF and SCORE were comparable with each other (Figure 4D). Considering the performance improvement of ICAnet (Figure 4A–C), it is tolerable that the running time of ICAnet was approximately three times longer than that of SCORE (Figure 4D).

### ICAnet facilitates functional interpretations of mouse brain dataset

**Figure 5.**
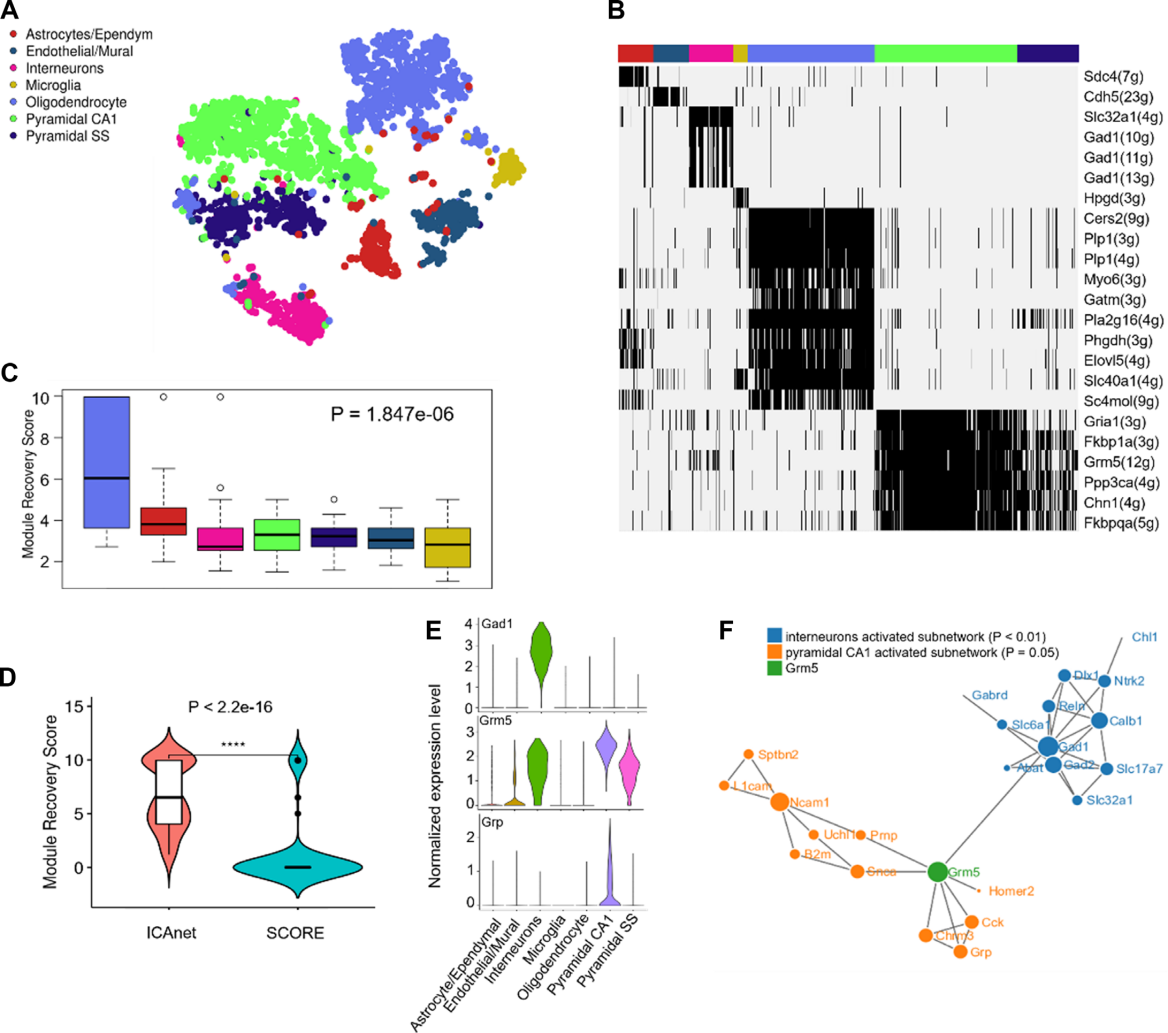
ICAnet facilities functional interpretation of cell clusters from mouse brain. (**A**) ICAnet groups 3005 mouse brain cells (the data by Zeisel *et al.*) into seven clusters shown by t-SNE. Each color represents a cell type annotated by the original authors. (**B**) Heatmap presentation of binarized modules identified by ICAnet for seven cell types. Black signal denotes the active status of a certain network module, the hub gene of each module is used for presentation. The number of genes in each module is denoted in the parenthesis. The colors of the horizontal bar at the top denote the same cell types as those indicated in panel a. (**C**) Boxplot displaying the distribution of module recovery scores, which reflects the reproducibility of the inferred modules in independent mouse oligodendrocyte single cell dataset. The *P*-value was based on Wilcoxon rank sum test. The box colors denote the same cell types as those indicated in panel (A). (**D**) Violin plot of oligodendrocyte-associated module recovery scores between ICAnet and SCORE. The *P*-value was calculated by Wilcoxon rank sum test. (**E**) Violin plots displaying the expression levels of *Gad1*, *Grm5* and *Grp* in each cell type annotated by original authors. (**F**) The sub-networks (modules) of interneurons and pyramidal CA1. Each dot represents a gene and each line means the interaction of two genes. The size of the dot reflects the importance (degree of connection) of the gene in the network.

To evaluate the efficiency of ICAnet in aiding cell clustering and biological interpretation of scRNA-seq datasets, we performed further investigations on the widely used mouse brain scRNA-seq dataset (Dataset DS4, see Supplementary Table S1) (42). The ICAnet analysis of this dataset identified 1078 ‘activated’ sub-networks (or modules), and clustered the cells into seven expected cell types (oligodendrocyte, astrocyte/ependymal, endothelial/mural, interneurons, microglia, pyramidal SS and pyramidal CA1) (Figure 5A) as reported in the original research. To infer the potential biological functions of each cell cluster, we binarized module activity values and identified cell type-specific modules (Figure 5B, detailed in ‘Materials and Methods’ section). Notably, oligodendrocytes, which are neuroectodermally derived glial cells that have a major role in myelinating central axons (68), had the largest number of activated modules (Figure 5B). To assess the reliability of ICAnet in detecting cell type-specific networks, we used an additional scRNA-seq dataset of mouse oligodendrocytes (43) to examine whether ICAnet could re-discover the activated modules specific for oligodendrocytes. We used the Monte Carlo method (39) to calculate the module recovery score (MRS, see ‘Materials and Methods’ section for details) for each sub-network (Zeisel *et al.*) to evaluate its recurrence in the independent oligodendrocyte single-cell dataset. As expected, only the activated modules in oligodendrocytes had much higher MRSs compared with those of other cell types (Wilcoxon test, *P*-value = 1.1e-08; Figure 5C). For comparison, we also calculated MRSs of the modules in oligodendrocytes detected by using ICAnet and SCORE (both use PPI information for integrative analyses). The MRSs of the modules detected by ICAnet were significantly greater than those detected by SCORE (*P*-value < 2.2e-16; Figure 5D), indicating that ICAnet has better network reproducibility than SCORE.

Signature genes have been widely used to identify/infer certain cell types (69). However, genes always function in the context of a network, and the same gene may play different, even opposite roles when interacting with different partners. Thus, revealing signature genes involved in a network and the expression status of their interacting partners in different subpopulations will facilitate our deeper understanding of these cell types in a certain tissue. For instance, ICAnet revealed that *Grm5*, which encodes a subunit of glutamate metabotropic receptor (mGluRs), whose role is to bind with the excitatory neurontransmitter glutamate (70), showed higher expression in three (interneurons, pyramidal SS and pyramidal CA1) out of seven cell types from the mouse brain (Figure 5E). Interestingly, we found that two distinct active subnetworks containing *Grm5* existed in interneuron and pyramidal CA1 cell types, respectively (Figure 5F). In the interneuron subnetwork, *Grm5* connects to *Gad1*, which helps the synthesis of GABA and plays an inhibitory role (71). However, in the pyramidal CA1 subnetwork, *Grm5* interacts with the gene *Grp* (Figure 5F), which is specifically expressed in pyramidal CA1 cells (Figure 5E) and known to enhance the excitatory synaptic transmission through facilitating glutamate release (72). This example indicates that ICAnet can identify cell-type-specific active networks to label the biologically relevant information in single-cell clusters.

### ICAnet has the ability to identify rare cell types

**Figure 6.**
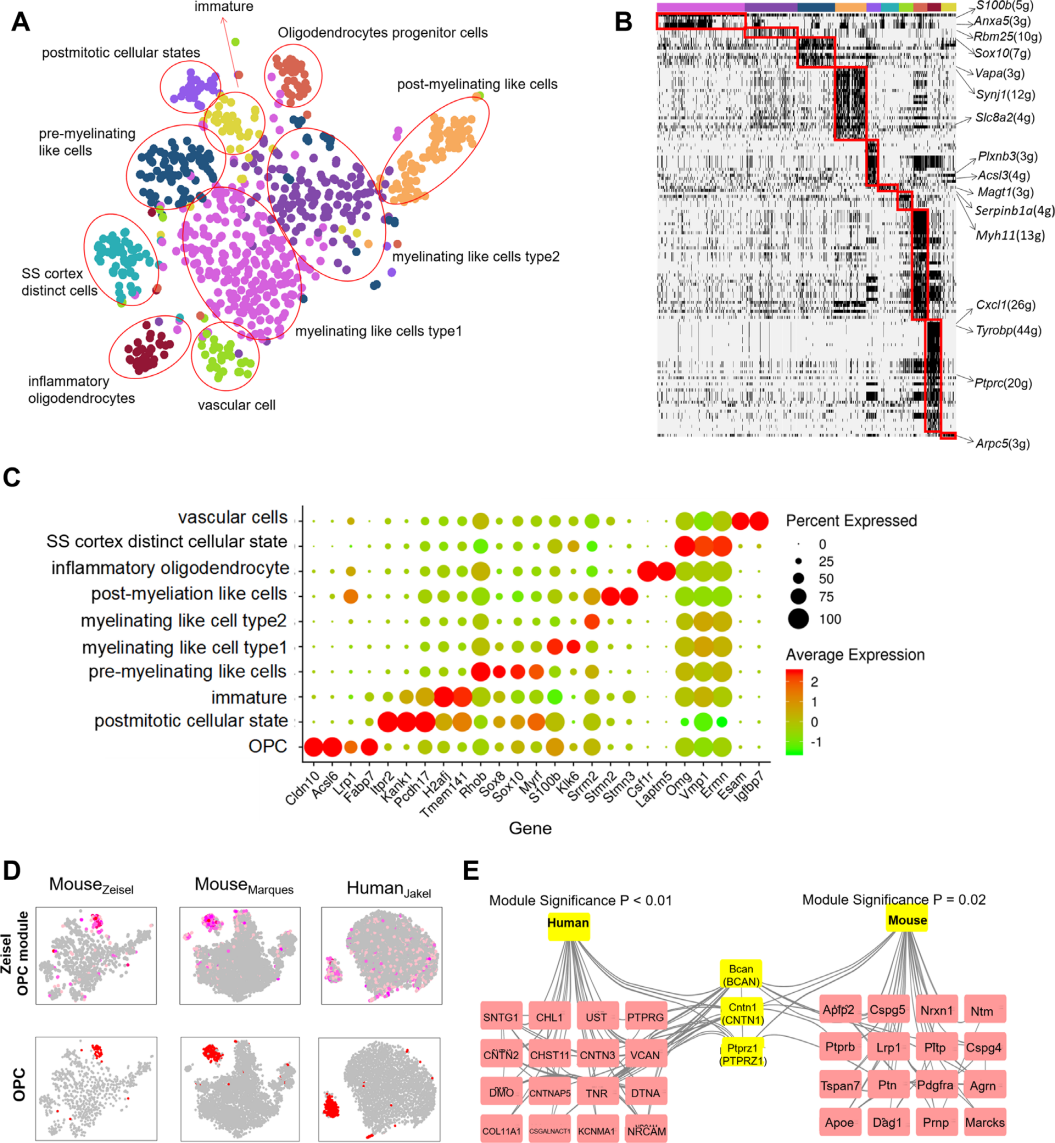
ICAnet dissects heterogeneous expression states of mouse oligodendrocytes. (**A**) t-SNE plot of ICAnet identified cell clusters in mouse oligodendrocyte. Cell clusters were circled and the cell types were annotated by marker genes. The colors of the horizontal bar at the top denote the same cell types as those indicated in panel (A). (**B**) Heatmap presentation of gene expression modules (labeled by red rectangle) specific to certain cell types/clusters (denoted by the horizontal color bars at the top) in mouse oligodendrocyte dataset analyzed by ICAnet. (**C**) Dotplot displaying the expression levels of representative marker genes of oligodendrocyte cell subgroups. Spot size denotes the percentage of cells expressing the gene within each cluster and color intensity denotes their expression level (*Z*-score transformed log_2_CPM value). (**D**) Activity of OPC associated modules derived from mouse brain dataset (by Zeisel *et al.*) in independent related single cell datasets from mouse (by Marques *et al.*) and human (by Jakel *et al.*). In the upper panel, color density represents the intensity of module activity. In the bottom panel, red spots represent cells defined as OPC. (**E**) Gene network in OPC associated modules inferred from mouse and human oligodendrocyte scRNA-seq data. Three shared genes were labeled yellow.

We went on to inspect whether ICAnet could identify rare cell types that were usually hard to discover using regular analysis methods. A previous study had found that oligodendrocytes could be further classified into six subpopulations using the BackSPIN clustering method (42). However, we found that they could be divided into 10 subpopulations with ICAnet (Figure 6A and B), and each subpopulation was supported by signature gene(s) (Figure 6C). Some of the newly-found cell subpopulations were of biological significance (Supplementary Figure S7). Oligodendrocyte progenitor cells (OPCs), a subtype of glial cells that can differentiate to oligodendrocytes in the central nervous system, was discovered by ICAnet but missed in the original study (Figure 6A–C). This was supported by several lines of evidence, for example, ICAnet identified a *Ptprc*-centered subnetwork in the OPCs (Figure 6A and B; Supplementary Table S2). PTPRC (also known as CD45) is a key phosphatase involved in OPC differentiation (73). Genes in this network were also enriched in GO terms related to OPC differentiation, such as glutamate metabolic process and oligodendrocyte differentiation (Figure 6A and B; Supplementary Table S2).

To examine whether the activated modules in the OPCs identified by ICAnet exist across different datasets, we analyzed two more scRNA-seq datasets derived from mouse oligodendrocytes (43) and human oligodendrocytes (74). The activated modules in the OPCs discovered above also existed in these two validation datasets (Figure 6D). Thus, ICAnet could sensitively identify sub-networks specific to rare cell types and improve the cell-type interpretation of scRNA-seq data. Further investigations revealed that the OPC network existing in both mouse and human contained three orthologous genes, *Bcan*, *Cntn1* and *Ptprz1* (Figure 6E). A literature search largely supported the functional OPC-relevance of these three genes. *Bcan* encodes a member of the lectican family of chondroitin sulfate proteoglycans, which are usually highly expressed in gliomas and a subtype of the OPCs, and may promote the growth and cell motility of brain tumor cells (74); *Cntn1* encodes the cell adhesion molecule contactin 1, which has been proven to bind to its ligand PTPRZ1. The PTPRZ1/CNTN1 complex represses OPC proliferation and promotes oligodendrocyte maturation and differentiation (73). *Ptprz1* encodes Protein Tyrosine Phosphatase Receptor Type Z1 (PTPRZ1), which is expressed in both adult and fetal human OPCs and has been reported to regulate the tyrosine dephosphorylation of β-catenin, the key Wnt pathway intermediate (75). This shared sub-network and related genes in specific cell types across species may have some evolutionary implications worthy of further study.

Moreover, we examined the ability of ICAnet to infer rare cell types by combining the pancreas-originated scRNA-seq datasets generated by different construction strategies (Dataset DS5, see Supplementary Table S1) (60–62). Interestingly, a rare cell type was identified when combing these scRNA-seq datasets (Supplementary Figure S8A and B). Further analysis suggested that this rare cell type was a subpopulation of beta cells that underwent endoplasmic reticulum (ER) stress, as evidenced by the high expression of ER stress-related marker genes *DDIT3* and *PPPR15A* (Supplementary Figure S8A and B). Additionally, the activated sub-network in this cell type contained eight more genes, including *KRT8, HSPA5, XBP1, DNAJB9, PDIA4, MANF, HSP90B1* and *CRELD2* (Supplementary Figure S8C and D), all of which are associated with the ER-stress pathway (76–83). The hub gene of this activated sub-network, *HSPA5* (Supplementary Figure S8C), is the central mediator of ER stress and can be quickly induced by the unfolded protein response (UPR) upon ER stress (84). These lines of evidence support the notion that ICAnet identifies a rare cell type relevant to ER stress in the pancreas.

### ICAnet identifies developmental trajectories using time-course scRNA-seq datasets through batch-effect correction

**Figure 7.**
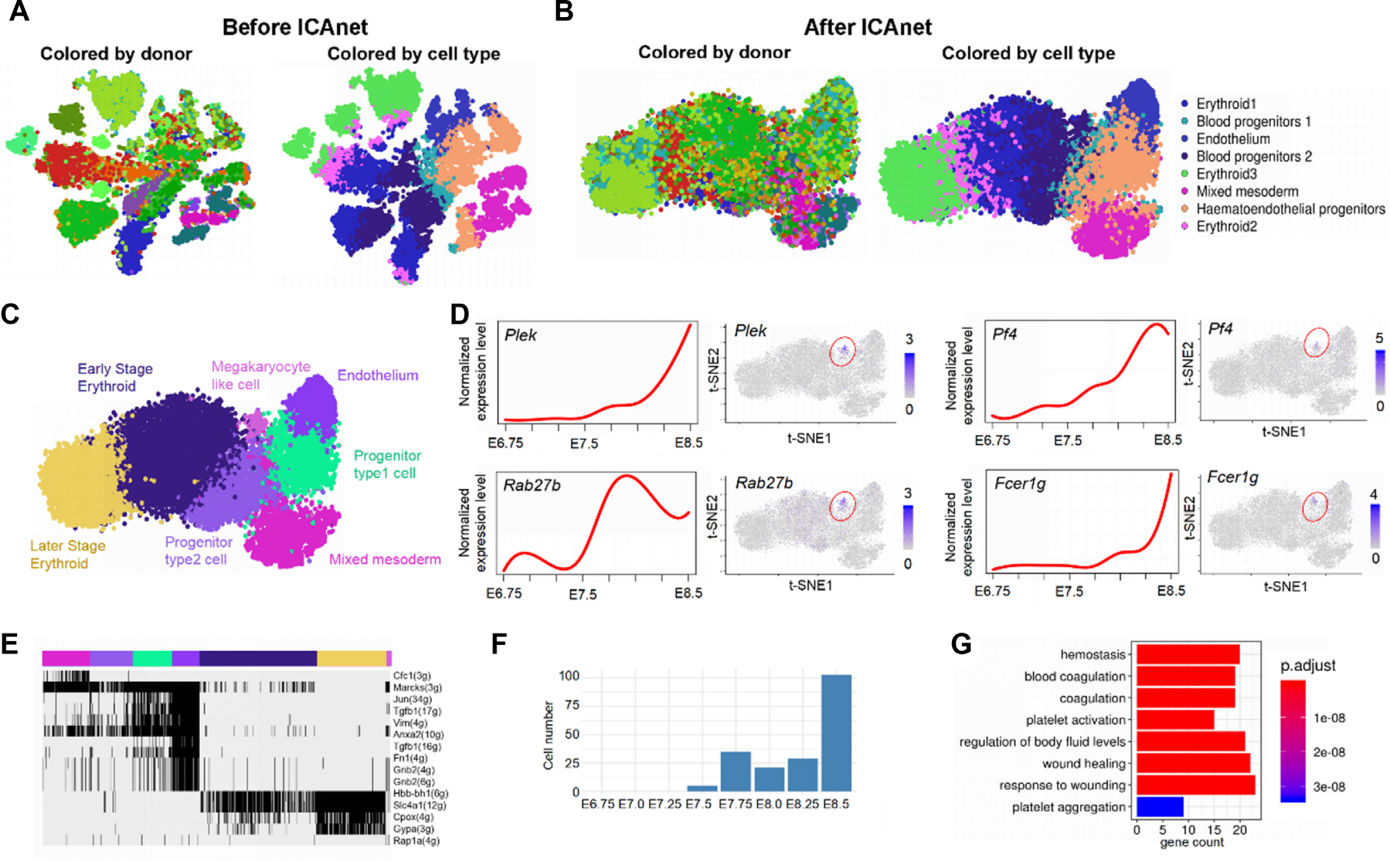
ICAnet identifies a rare megakaryocytes like cell type through integration analysis of time course hematopoietic datasets. (**A** and**B**) t-SNE visualization of cell clustering results of scRNA-seq datasets generated from mouse hematopoietic cells of eight embryonic stages (E6.75–E8.5) (86) analyzed without (A, PCA-based Seurat method) and with (B) ICAnet. Cell types defined by original authors were used for presentation. (**C**) Seven cell types were identified by applying Louvian clustering algorithm based on module activity. (**D**) The log_2_-transformed expression level (counts per 10 000, CP10K) of four key genes related to megakaryocytes cell identity was shown during eight embryonic development stages (left). Cells highly express these four genes were highlighted by red circles in the t-SNE plots (right). (**E**) The activity of representative modules was binarized and visualized through heatmap to show their regulation pattern in different cells. Each module is labeled according to their hub gene. The number of genes within each module was indicated in the parenthesis. (**F**) The barplot of number of megakaryocyte-like cells in different embryonic development stages. (**G**) GO enrichment analysis by clusterProfiler for genes highly expressed in megakaryocytes like cells. The top eight significant GO terms were shown. Bar length (*X*-axis) denotes the gene number and the color key denotes the Benjamini–Hochberg adjusted *P*-values.

Time-course scRNA-seq datasets are important for revealing crucial biological processes during development. Current integrative analysis methods applied on time-course datasets remove batch effect with the risk of eliminating real biological signals (85). As demonstrated above, ICAnet can characterize the similarities of co-expression structures among different batches. To investigate how well ICAnet performed on time-course scRNA-seq datasets, we analyzed scRNA-seq data of 15 022 cells from eight time points (E6.75 to E8.5) during mouse embryonic hematopoiesis (86) (Dataset DS6, see Supplementary Table S1). We first analyzed the data without batch-effect correction step (no ICAnet) and found that cells largely grouped according to their batches rather than cell type (Figure 7A), suggesting that a severe batch effect existed. However, ICAnet analysis on the same datasets grouped the mixed cells from different mice according to known cell types (Figure 7B). ICAnet also constructed a smooth and continual trajectory structure in the t-SNE manifold space (Figure 7B), consistent with hematopoietic development process (86). For comparison, other methods including SCORE, Combat and Harmony, were also used to analyze the same dataset. Both SCORE and ICAnet revealed continual differentiating processes with t-SNE and UMAP, uncovering a branching trajectory structure representing the processes from mixed mesoderm and hematoendothelial progenitor populations to differentiated endothelial and erythroid populations (Supplementary Figure S9). In contrast, Harmony and Combat resulted in relatively discrete cell distributions (Supplementary Figure S9). These results suggest that ICAnet can reliably capture developmental trajectories during blood cell differentiation. To further test the capacity of ICAnet in developmental trajectory inference, we applied ICAnet to our recently sequenced mouse testis scRNA-seq dataset to predict the differentiation trajectory during spermatogenesis. ICAnet generated a differentiating trajectory consistent with that produced by DDRtree-based embedding methods (see Supplementary Notes, Sections 8 and 9 in the Supplementary Materials).

Additionally, ICAnet identified a new type of cells (Figure 7C–E) not reported in the original study (86). The number of these cells is relatively small and featured an activated module centered on *Rap1* (Figure 7E). This cluster also showed high expression level of four key genes (*Plek, Rab27b, Pf4* and *Fcer1g*) that are crucial for the megakaryocyte cell identity and function (Figure 7D). Consequently, we named this novel cell type megakaryocyte-like cells. Interestingly, the expression levels of these four genes increased with development stages (Figure 7D), and the number of cells belonging to this type also increased accordingly (Figure 7F). What's more, highly expressed genes in this cluster were enriched in GO terms like platelet activation, coagulation and platelet aggregation (Figure 7G), in line with the role of megakaryocytes (87,88). Together, ICAnet can reveal developmental trajectories and even rare cell types from time-course scRNA-seq datasets.

### ICAnet discovers activated modules that may act as prognostic markers for AML patients

**Figure 8.**
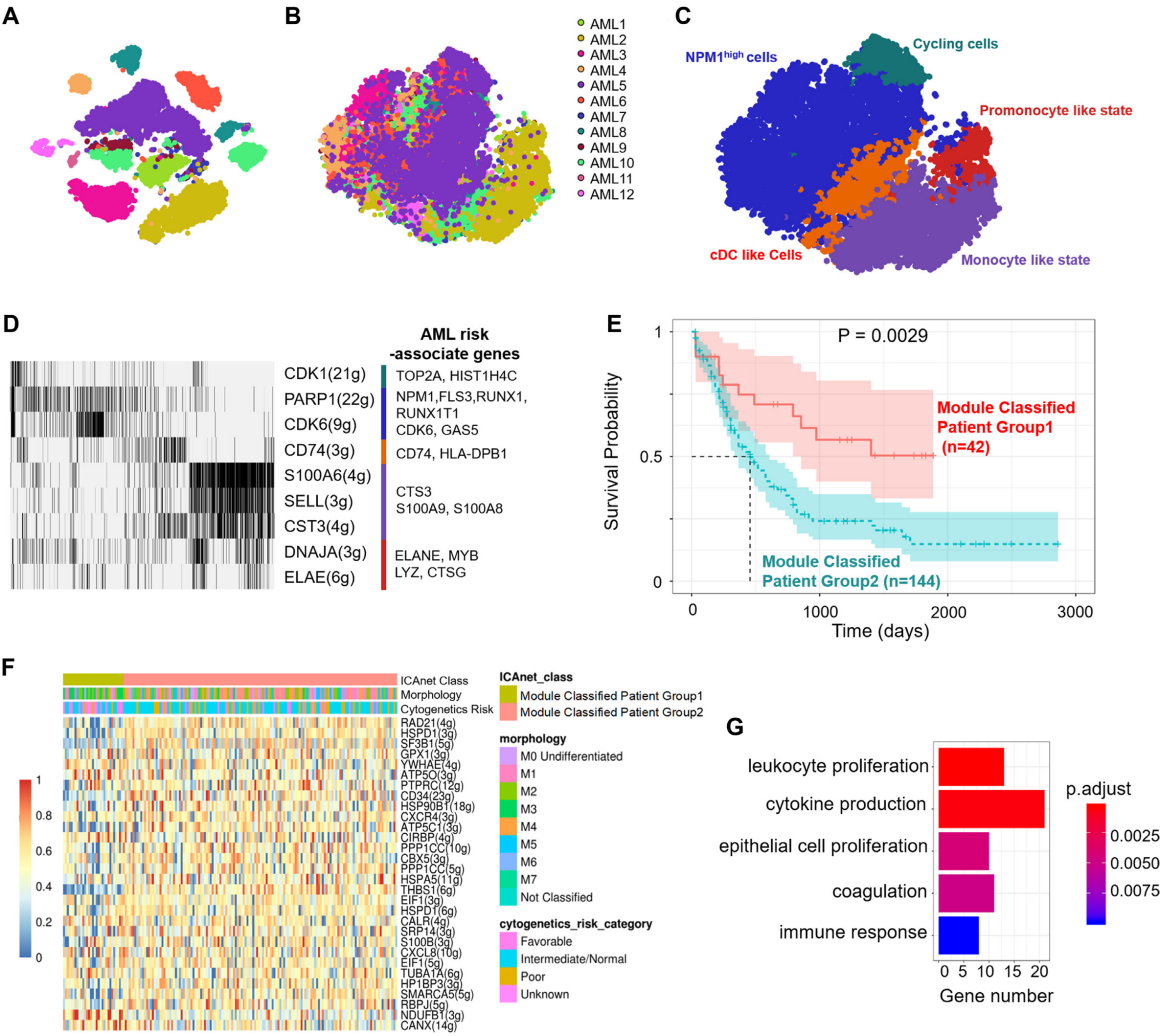
ICAnet-identified active module from scRNA-seq is a prognosis marker for AML patients. (**A** and**B**) t-SNE plot based on the expression matrices without (A) or with (B) ICAnet analysis on scRNA-seq datasets from 12 patients’ day 0 leukemia sample. (**C**) Five main express states of AML malignant cells identified based on ICAnet defined modules. (**D**) Heatmap representation of activated modules identified by ICAnet in AML scRNA-seq dataset. Gene number and the hub gene in each module were indicated on the right. Color bar denotes the expression state in panel (C) and AML risk associated genes involved were indicated on the right. (**E**) The survive curves of 186 AML patients (from TCGA) stratified by prognosis modules identified by ICAnet. *P*-value was based on log-rank test. *X*-axis denotes the survival days and *Y*-axis represents the survival probability. (**F**) Heatmap showing activity of 30 prognosis modules (rows) across 186 bulk AML expression profiles in TCGA. Color key represents the value of module activity. (**G**) GO enrichment analysis by clusterProfiler for genes in the prognosis modules. The X axis denotes the number of the related genes and the color key denotes the Benjamini–Hochberg adjusted *P*-values.

As ICAnet can identify biologically meaningful gene-expression modules, we were curious whether it could discover modules that would be useful for survival analysis of cancer patients. We performed ICAnet analysis on publicly available scRNA-seq datasets from 12 patients with acute myeloid leukemia (AML) (89) (Dataset DS7, see Supplementary Table S1). ICAnet largely eliminated batch effect and correctly grouped the malignant cells without obvious donor effect (Figure 8A and B). We also compared the ICAnet clustering using four other algorithms (SCENIC, SCORE, Harmony and Combat) with previously defined cell labels (89) as references to evaluate the clustering performance. We used ARI and LISI to evaluate the five methods comprehensively, and the result showed that ICAnet performed better integration (based on F1_LISI_ and F1_ARI_ scores) than the other methods (Supplementary Figure S10, see Supplementary Notes, Sections 2 in the Supplementary Materials). By integrating all the scRNA-seq data derived from malignant cells from the 12 AML patients, ICAnet clustered these AML cells into five major groups (Figure 8C and Supplementary Figure S11A–C), each having a distinct activated module (Figure 8D; Supplementary Figure S11A and D). The first group corresponded to the cycling-cell-like state, wherein a module with the hub gene *CDK1* was activated (Figure 8D). CDK1 is a cyclin-dependent kinase that interacts with RARγ to influence cell-cycle progression and cellular differentiation in AML (90). The second group corresponded to the nucleophosmin 1 (*NPM1*)^+^-like state, wherein modules with three hub genes (*NPM1, PARP1* and *CDK6*) were activated (Figure 8D). NPM1 is a nucleolar phosphoprotein with diverse biological functions (including molecular chaperoning, ribosome biogenesis, DNA repair and genome stability) and has been implicated as a famous prognostic marker for AML (91). ICAnet also discovered other well-known AML molecular markers in the NPM1 module, such as *FLT3, RUNX1* and *RUNX1T1*. Gene set enrichment analysis (GSEA) on the curated gene set (see ‘Materials and Methods’ section) revealed that these NPM1^+^-like cells were enriched with AML risk-associated genes and leukemic stem cell marker genes (Supplementary Figure S11E). The third group was dendritic like cell, wherein modules with the hub genes *LYN* and *CD74* were activated (Figure 8D). Notably, both *LYN* and *CD74* play important functions in dendritic cells (92,93). The fourth group was promonocyte-like cells, wherein modules with the hub genes *ELANE* and *DNAJA1* were activated (Figure 8D). The final group was monocyte-like cells, wherein modules with the hub genes *S100A6*, *SELL* and *CST3* were activated (Figure 8D).

Since the proportions of these five types of cells may change as AML progresses, we hypothesized that activated gene modules may serve as prognostic markers for AML patients. To validate this idea, we incorporated bulk RNA-seq data and survival data to further examine whether ICAnet-defined modules could group AML patients with different survival curves. A dataset of 562 AML patients (Gene Expression Omnibus (GEO) accession number GSE37642) was used as the training dataset. We first calculated the activity of the ICAnet-predicted modules in this training dataset using GSVA (detailed in ‘Materials and Methods’ section), and then applied bootstrap LASSO (see ‘Materials and Methods’ section) to related modules and patient survival information in the training cohort. Next, according to the recurrence rate (detailed in ‘Materials and Methods’ section), we selected the top 30 modules identified in malignant cells and used them to predict patient survival, based on the survival prediction performance of the patients in the training dataset (Figure 8E and F). Interestingly, three genes (*CXCR4*, *GPX1* and *SF3B1*) among these modules had already been reported as prognostic markers. For example, the chemokine receptor CXCR4 mediates cell anchorage in the bone marrow micro-environment and over-expressed in 25–30% of patients with AML (94). Besides, *CXCR4* is associated with poor prognosis in AML patients with and without the *FLT3* mutation (95,96).

We next tested these 30 modules in expression datasets related to AML from The Cancer Genome Atlas (TCGA) (97). Using the unsupervised clustering method PAM, we found patients were clearly separated into two groups with statistically different survival statuses (Figure 8E and F). Interestingly, most modules were upregulated in the poor prognosis group (Figure 8F). GO analysis found that genes belonging to these upregulated modules were enriched in functional terms, such as leukocyte proliferation, cytokine production, epithelial cell proliferation, coagulation and immune response (Figure 8G). Furthermore, we compared our newly identified prognostic factors with well-known AML prognostic markers (98–101), The result showed that ICAnet defined modules were the most significant prognostic markers across all known molecular markers (*P-*value = 0.02; Supplementary Table S3). Thus, ICAnet has the ability to discover new prognostic markers by discovering activated modules specific to certain cell types in scRNA-seq datasets, at least for AML blood cancer.

## DISCUSSION

Single-cell transcriptome analyses have been increasingly applied to reveal cellular heterogeneity in a tissue, which is important for understanding its biological roles and even the pathological state of a diseased tissue. Based on the idea that different types of cells have different gene–gene interaction networks, recent bioinformatics tools, such as SCENIC and SCORE, began to adopt gene co-expression networks to perform cell clustering and biological interpretations in scRNA-seq data analysis. With the unprecedented increase in publicly available scRNA-seq data, integrative analysis capable of discovering new knowledge has become extremely important, although it is still a challenging task. Batch-effect correction is one of the key obstacles that needs to overcome in integrating analysis of multiple datasets. Current batch-effect correction methods usually adopt a strategy searching for nearest neighbor cells across different batches and then applied different weight or transformation schemes to construct a corrected expression matrix or cell embedding vectors (51,53). Although these strategies have been widely used in multi-batch scRNA-seq data integration, most of them are weak in biological interpretations of the data. Methods based on gene co-expression networks, such as SCENIC (11) and SCORE (12), concatenate all the analyzed datasets into one dataset and then directly apply correlation learning algorithms (random forest importance and Pearson's correlation) to identify co-expression modules. Such direct data merging ignores batch-specific properties and, thus, may result in certain false positive correlations that impair the batch-effect correction. In contrast, to improve the batch-effect correction efficiency, ICAnet learns shared and independent expression programs from different datasets and also integrates PPI network information (Figure 1A). The ability of ICAnet to efficiently perform batch-effect corrections was validated using scRNA-seq data of various conditions (tissues/donors/library-type), and they all indicate that ICAnet can largely eliminate batch effect originating from multiple sources.

In addition to its batch-effect correction ability, ICAnet can also detect cell types (or expression states) through local co-expression modules of functionally relevant genes (Figure 1B), which enables rare cell type discovery. Currently network-based clustering algorithms, such as SCIENC and SCORE, tend to miss the gene co-expression structures of rare cell types possibly owing to gene–gene correlations are calculated based on all the cells. ICAnet decomposes the gene expression of single cells into a number of independent components, with each component linked to a certain number of activated modules. By analyzing both simulated and real scRNA-seq data, the accuracy and robustness of ICAnet were also validated. The theoretically valid concept was also practically confirmed using three datasets of different tissue origins, brain (both mouse and human), pancreatic islet and blood cell development (hematopoiesis with time courses). ICAnet identified a rare cell type (OPCs) in the mouse brain single-cell dataset. In addition, the activated gene modules in OPCs identified by ICAnet were also found in two independent scRNA-seq datasets from both mouse and human oligodendrocytes, supporting the robustness of ICAnet in identifying rare cell types. ICAnet also identified a rare type of beta cells under ER stress in pancreatic islet. Multiple lines of evidence support the reliability of this conclusion. First, the ER-stress marker genes *DDIT3* and *PPPR15A* were highly expressed in this cluster; second, the active sub-network of this cluster also contained eight more genes associated with the ER-stress pathway; and lastly, the hub gene *HSPA5* of this active sub-network in the cell type is a central mediator of ER stress. In the scRNA-seq dataset of mouse blood cell development with eight time points during hematopoiesis, ICAnet revealed the developmental trajectories of the blood cells and also identified a novel rare cell type missed by the original study (86). This rare cell type showed high expression levels of four genes that play important roles in megakaryocyte cell identity. Interestingly, their expression increased with development stage and the functional enrichment analysis also supported their megakaryocyte-like features. Together, we demonstrated by three independent datasets from different tissues to show that ICAnet is a powerful tool to discover biologically meaningful rare cell types for further study.

Information and knowledge of PPIs in human and mouse have been increasingly accumulated in recent years (102). Thus, using PPIs as the backbone to discover gene-expression modules in scRNA-seq analyses is now feasible. Previous studies have revealed that genes with PPIs showed co-expression trends at the RNA level (16,103), suggesting that integrating PPI information into scRNA-seq data analyses could be beneficial. In practice, we performed multiple simulations and statistical tests to demonstrate the importance of incorporating PPI networks into cell clustering and module interpretations of scRNA-seq data (see Supplementary Notes, Sections 2 and 3 in the Supplementary Materials). We also applied ICAnet on other species (*Drosophila* as an example) and the results showed that ICAnet could be extended to other species through incorporating species-specific PPI network (see Supplementary Notes, Section 7 in the Supplementary Materials). These analyses results, combined with those from our comprehensive analysis on scRNA-seq datasets from different tissue/species, suggest that integrating PPI networks and gene expressions at the single-cell level could have great potential to reveal dynamic molecular regulatory mechanisms underlying different cell states.

In summary, we have shown the accuracy, robustness and reproducibility of ICAnet in single-cell transcriptome analysis. We demonstrated that ICAnet performs efficient cell clustering and batch-effect correction, which eventually facilitate the functional interpretation of the resulted cell cluster. Moreover, ICAnet also shows a promising capacity for discovering new prognostic markers by analyzing scRNA-seq data from patients of certain disease. We believe that ICAnet will benefit studies in multiple research fields that utilize scRNA-seq techniques.

## DATA AVAILABILITY

ICAnet is freely available at https://github.com/WWXkenmo/ICAnet/. The raw single-cell RNA-seq dataset of mouse whole testis (fastq format) has been uploaded to NCBI-SRA with the accession number PRJNA650016. The single-cell gene expression matrix and cell-type annotation by ICAnet is available at https://github.com/WWXkenmo/MouseGerm/. Besides, the gene lists correspond to related modules (Figures 5B, 6B, 7E, 8D and F) are provided as the Supplementary Table S4. For the detail information about the public datasets used in this manuscript, see Supplementary Notes, Section 10 in the Supplementary Materials and Supplementary Table S1.

## Supplementary Material

gkab089_Supplemental_Files
