# Supplementary material for: Independent component analysis based gene co-expression network inference (ICAnet) to decipher functional modules for better single-cell clustering and batch integration
Source: Nucleic Acids Res. 2021 Feb 22;49(9):e54. doi: 10.1093/nar/gkab089 (PMC8136772; doi:10.1093/nar/gkab089)
Supplement: gkab089_Supplemental_Files [file gkab089_supplemental_files.zip › Supplementary Materials.pdf]

Supplementary Materials

Weixu Wang[1], Huanhuan Tan[2], Mingwan Sun[3], Yiqing Han[4], Wei Chen[1], Shengnu Qiu[5], Ke Zheng[2,*], Gang Wei[1,*], Ting Ni[1,*]

[1] State Key Laboratory of Genetic Engineering, Collaborative Innovation Center of Genetics and Development, Human Phenome Institute, School of Life Sciences, Fudan University, Shanghai, 200438, P.R. China

[2] State Key Laboratory of Reproductive Medicine, Nanjing Medical University, Nanjing, 211166, P.R. China

[3] College of Life Science, South China Agricultural University, Guangzhou, 510642, P.R. China

[4] College of Agricultural, South China Agricultural University, Guangzhou, 510642, P.R. China

[5] Division of Biosciences, Faculty of Life Sciences, University College London, London, UK

*Correspondence: kezheng@njmu.edu.cn; gwei@fudan.edu.cn; tingni@fudan.edu.cn

Table of contents:

1. Supplementary Notes 1

Section 1. Determining the number (k) of independent components of a scRNA-seq dataset 1

Section 2. Illustration of evaluation metric used in the present study 4

Section 3. Justification of integrating PPI and ICA in scRNA-seq datasets 10

Section 4. Evaluation of biological significance for predicted modules 16

Section 5. Evaluation of statistical significance for predicted modules 23

Section 6. Incorporating TF-regulons into ICAnet (ICAnetTF) 28

Section 7. Integrating PPI from different species 29

Section 8. Dimensionality reduction methods used in ICAnet 32

Section 9. Using ICAnet to identify cell differentiation trajectory on mouse whole-testis single cell RNA-seq dataset 33

Section 10. Datasets used in the present study 35

2. Supplementary Figures 41

3. Supplementary Tables 55

1. Supplementary Notes

Section 1. Determining the number (k) of independent components of a scRNA-seq dataset

Determining the number (k) of components through Random Matrix Theory: Determining the number of components of a scRNA-seq dataset is important but very challenging. To address this issue, we used random matrix theory (RMT) (1,2) to estimate the dimension number of a given gene correlation matrix by comparing the statistics of the observed eigenvalues of the data correlation matrix. The “random” correlation matrix was defined through the formula below.

$$\mathrm{R} = \frac{1}{L} A A^T$$

where A is a matrix containing L cells and N genes with zero mean and unit variance. Statistical properties of random matrices such as R is known. In the limit N → ∞, L → ∞, such that Q = L/N is fixed. It was shown analytically that the distribution $P_{rm}(\lambda)$ of eigenvalues λ of the random correlation matrix R is given by (2).

$$P_{rm}(\lambda) = \frac{Q}{2\pi} \frac{\sqrt{(\lambda_+ - \lambda)(\lambda - \lambda_-)}}{\lambda}$$

where λ_+ and λ_- stand for the maximum and minimum eigenvalues of R respectively, given by

$$\lambda_\pm = 1 + \frac{1}{Q} \pm 2\sqrt{\frac{1}{Q}}$$

So the theoretical maximum and minimum eigenvalues for random matrices of any size are known. Therefore, the number of expression programs/components can be determined by the number of eigenvalues that are significantly different from this analytical maximum eigenvalues.

The eigenvalue can be computed through svds function in R package ‘rARPACK’. It can find largest k singular value/eigenvalue (eigenvalue and singular value are exactly the same under the setting of correlation matrix) from the correlation matrix A through Truncated Singular Value Decomposition, which computes eigenvalues on large

correlation matrix very efficiently.

Estimating the number of gene expression programs with ICA+RMT: To determine the number of expression programs (k) more appropriately, we hypothesized that k needs to be at least larger than the number of intrinsic cell types (expression states). Previous studies have shown that RMT can be applied to estimate the number of latent cell states (3). Practically, we found that the number of RMT-estimated dimensions could be influenced by the number of cells and highly variable genes in the expression matrix (Figure1 a,b). When sample size of gene expression data getting larger, RMT will give overestimated number of cell types, which may be caused by the increase in cell expression heterogeneity or stochastic signal as the number of cells increases.

To address above issue, we combined RMT and ICA to estimate the number of cell types. First, we used RMT to estimate the latent dimension of transcriptome data (denote as K), and then used ICA to decompose the transcriptome matrix X into source matrix $S^{n \times K}$ and mixture matrix $A^{m \times K}$ with the dimension estimated by RMT. The source matrix represents independent expression program, and mixture matrix represents each program's contribution magnitude of specific sample. So, the gene profile is considered to be a linear mixture of statistically independent expression programs, and the mixture matrix measures expression program variation across tested samples. We then re-applied RMT on mixture matrix to re-estimate latent dimensions. And we found this approach could efficiently predict the number of cell types and could not be influenced by the sample size (Figure1 c,d,e,f).

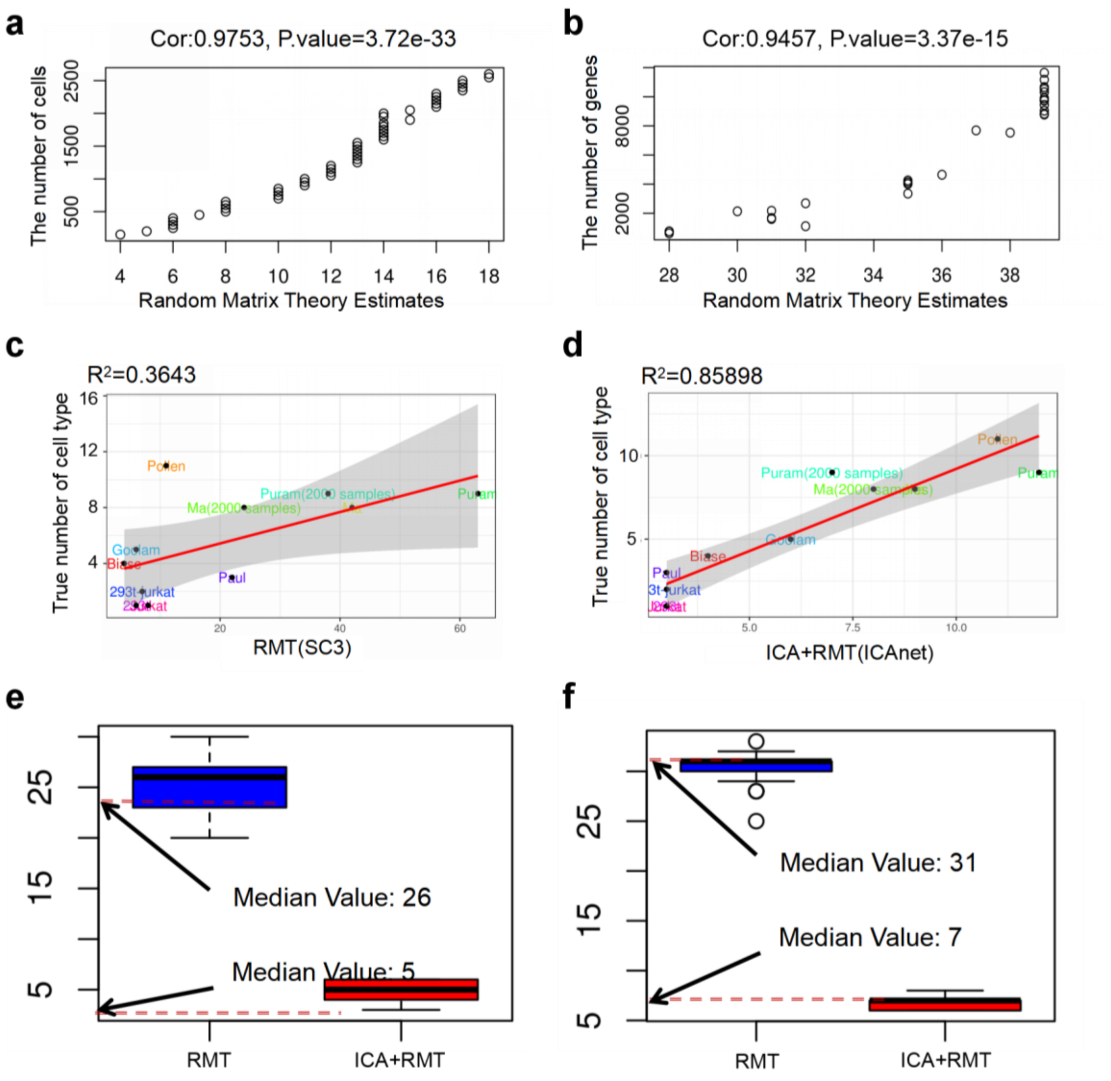

Figure 1. ICA+RMT predicts the number of cell types (or states) better than RMT only strategy.

a. Positive correlation between the numbers of cell types estimated by RMT (SC3) (X axis) and the numbers of cells randomly sampled from mouse brain scRNA-seq dataset (range from 500 cells to 3005 cells). Each black circle represents a simulated result. Cor means Pearson correlation coefficient. The correlation significance P value was calculated through T-test.

b. Positive correlation between the numbers of cell types estimated by RMT (SC3) (X axis) and the numbers of highly variable genes randomly sampled from mouse brain scRNA-seq dataset (range from 1000 genes to 10000 genes). Each black circle represents a simulated result. Cor means Pearson correlation coefficient. The

correlation significance P value was calculated through T-test.

c. Comparison between numbers of cell clusters predicted by two-step RMT (ICA+RMT) (X axis) and reference ones (Y axis, reported by original authors). Ten dots in the plot denote the 10 datasets used for comparison. Red line represents the regression line and grey area means confidence interval of regression line. R^2: coefficient of determination.

d. Comparison between numbers of cell types predicted by RMT (X axis) and the reference ones (Y axis, reported by 10 original authors). All the labels have the same meaning as that in panel c.

e-f. Boxplots of predicted cell type number based on different number of assayed cells (e, from 1000 to 3005 cells, 100 times) or variable genes (f, from 3000 to 5000 genes, 100 times). Comparison between RMT+ICA (ICAnet) and RMT only (SC3) was performed.

One thing worth mentioning is that the heterogeneity of cell expression states may be the results from real biological effect or artificial technical effect. A series of technical variables (such as the sequencing depth, the number of detectable genes across samples, and the number of single cells) can influence the predicted cell expression states. We suggest users to perform '*SCTransform*' (4) normalization or denoise preprocessing steps (1,5) before running RMT to estimate the number of expression programs, especially when users need to integrate datasets generated from different library types.

Section 2. Illustration of evaluation metric used in the present study

ARI: Supposing there are two different cell class vectors u,v, we can summarize the overlapping elements of these two vectors to create a contingency table n, let n_{ij} be the number of objects that are in both vectors. Let $n_{i.}$ and $n_{.j}$ be the number of objects in corresponding class u_i and class v_j respectively. Then the ARI can be defined as:

$$\text{ARI} = \frac{\sum_{i,j}\binom{n_{ij}}{2} - [\sum_i \binom{n_{i.}}{2}\sum_j \binom{n_{.j}}{2}]/\binom{n}{2}}{\frac{1}{2}\left[\sum_i \binom{n_{i.}}{2} + \sum_j \binom{n_{.j}}{2}\right] - [\sum_i \binom{n_{i.}}{2}\sum_j \binom{n_{.j}}{2}]/\binom{n}{2}}$$

LISI: LISI calculates batch probability on the local distribution around each cell. To be more sensitive to local diversity, LISI used Gaussian kernel-based distribution of neighborhoods. After calculating each batch probability based on this distribution, LISI used the inverse Simpson's Index to measure the effective batch numbers in each cell's neighborhood, which defined as follow:

$$\text{LISI} = \frac{1}{\sum_{b=1}^{B} p(b)^2}$$

where B represents the number of batches.

Evaluation of batch effect correction on multiple batches (>10): Performance evaluation of different methods on integrating multiple-batch scRNA-seq datasets is much more complicated and a trade-off between the following two aspects that need to be considered.

1. The cells from the same cell type and different batches need to be mixed up after integration.

2. The batch-specific cell type needs to be preserved after integration.

Ideally, the integration shall remove all non-biologically relevant noise while preserve all biologically relevant information. However, in most realistic cases, the biological and non-biological signals are promiscuous and hard to be separated from each other (so called confounding effects), especially for scRNA-seq datasets. This makes the integration and evaluation very challenging.

In the scRNA-seq datasets derived from 12 AML patients, we observed batch-specific cell types based on the annotation in the original publication (6) (Figure 2 below). For instance, the ProMono-like cell type was enriched in sample AML1, AML2 and AML10. The cDC-like cell type was enriched in AML9 and AML14. Actually, no cell type exists across all 12 patients (Figure 2). These observations indicate that we need to be cautious when the batch mixing value (iLISI) is high, because it could be, at least

in part, induced by batch effect over-correction.

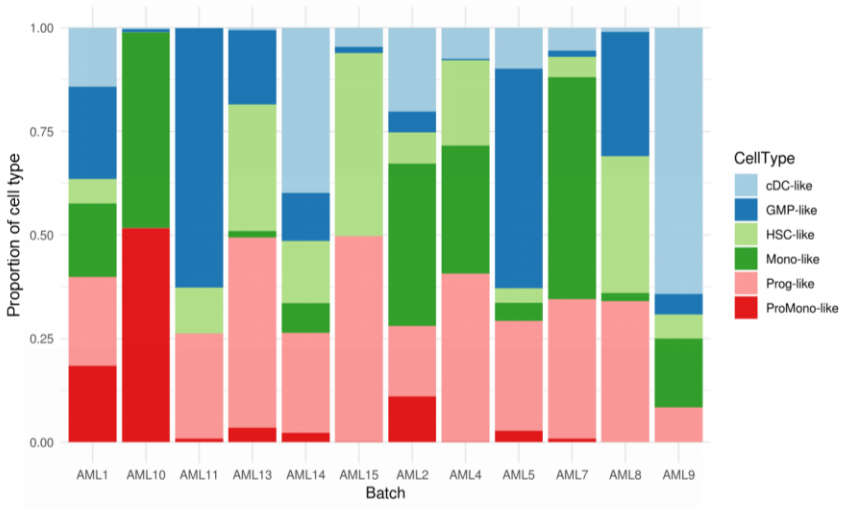

Figure 2. The proportion of each cell type in each batch (or donor) in AML scRNA-seq datasets. The cell types and their proportions are based on the information of the original publication.

Given that the metric measuring the batch mixing effect (iLISI) has intrinsic bias when batch-specific cell types exist, we correct it by considering the expected iLISI value of each cell type (annotated by the original publication) in each batch/sample. For example, the ProMono-like cell are enriched in three samples (AML1, 2, 10), therefore, the majority batch effective number of each cell (evaluated by iLISI) in this cell type shall be around three. Otherwise, if the majority batch effective numbers are much larger than three, it would have the problem of batch over-correction. We calculated the expected inverse local Shannon index (iLISI) of each cell type through the following formula:

$$E_k[iLISI] = \frac{1}{\sum_{n \in n_k} p(batch_n)^2}$$

in which, k indicates the kth cell type, and n_k indicates the batch IDs that appear in the kth cell type. We calculated the frequency of each batch (p) through measuring its proportion in the k cell types.

Therefore, for the cells in each cell type, their batch iLISI shall not be far from their expected iLISI. We could correct the batch iLISI through the following equation:

$$iLISI_i^{correct} = |iLISI_i - E_k[iLISI]|$$

which measures the absolute distance of each cell's iLISI with its expected iLISI. Therefore, if the corrected iLISI is small, it indicates that each cell is mixed as expected and has little over-correction.

We calculated the original iLISI and corrected iLISI in these 12 AML scRNA-seq datasets for comparison (Figure 3 below). Of note, a larger original iLISI value or a smaller corrected iLISI value stands for better batch mixing. Before correction, ICAnet has higher original iLISI value than SCORE and SCENIC while has lower original iLISI value than Harmony and Combat (Figure 3a). After correction, ICAnet has lower corrected iLISI value than SCORE and SCENIC (Figure 3b). The corrected iLISI value of ICAnet is comparable to that of Combat while higher to that of Harmony (Figure 3b). To examine whether batch over-correction could contribute to the better performance of Harmony and Combat (as reflected by the lower corrected iLISI values), we also analyzed the local purity of each cell type (cLISI) and found that Harmony and Combat had higher cLISI values, which indicate worse cell type local purity (Figure 3c).

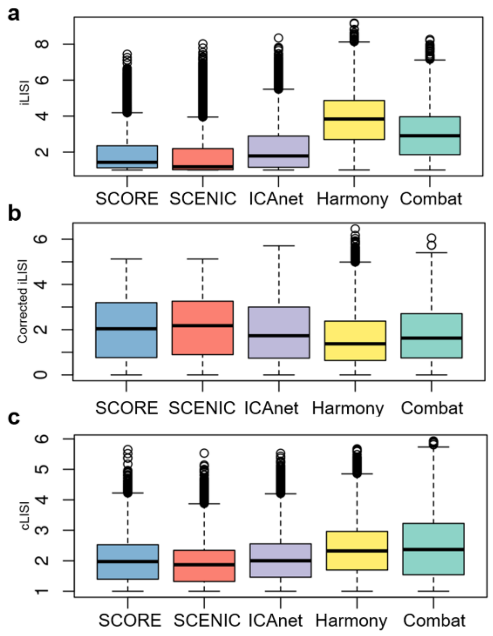

Figure 3. The performance evaluation between ICAnet and other four batch effect correction methods based on 12 AML scRNA-seq datasets.

(a) Boxplot of the original iLISI values of each method. (b) Boxplot of the corrected

iLISI value of each method. Both original (a) and corrected (b) iLISI values reflect the batch mixing around each single cell. (c) Boxplot of cLISI values of each method. cLISI reflects the cell type local purity around each cell.

Although ICAnet is not the best method regarding the two metric evaluation indexes (cLISI and iLISI, Figure 3), we have to remind that the sample integration (or batch effect correction) is a multi-object optimization process. And the two objects that need to be optimized can have intrinsic conflict. For example, optimizing the batch mixing may induce unwanted over-correction and therefore diminish the number of batch-specific cell types. On the other hand, optimizing the cell type purity may result in unsatisfied batch mixing. Therefore, we need to evaluate the overall performance of these methods by considering both cLISI and iLISI. This is the reason why we introduce $F1_{LISI}$ score (a combination of both cLISI and iLISI) in the main text. A higher $F1_{LISI}$ score reflects a better tradeoff between batch mixing and cell local purity. We found ICAnet has the highest F1 score of LISI (Table 1), although ICAnet is not the best in either cLISI or corrected iLISI. Moreover, we compared the $ARI_{cell\ type}$, ARI_{batch} and F1 score of ARI of each method. We found ICAnet also had the highest F1 score of ARI (Table 1). We noticed that Harmony and Combat had worse performance on cell clustering and cell type purity, as reflected by $ARI_{cell\ type}$ and cLISI (Table 1), supporting the notion that these two methods may have over-correction effect at least in the applications on these AML scRNA-seq datasets.

Table 1. Performance comparison among different batch effect correction methods by using F1 scores of LISI and ARI in 12 AML scRNA-seq datasets.

LISI

	cLISI	Corrected iLISI	$F1_{LISI}$ score
**ICAnet**	0.7864029	0.8027531	**0.79449389**
**SCORE**	0.7933553	0.7692886	0.781136621
**SCENIC**	0.8167645	0.7479967	0.780869504
**Harmony**	0.7240966	0.8792579	0.794169544
**Combat**	0.706731	0.8357116	0.765828556
**Raw**	0.8523066	0.6383356	0.729964099

ARI

	$ARI_{cell\ type}$	$1\text{-}ARI_{batch}$	$F1_{ARI}$ score
**ICAnet**	0.3080992	0.8270135	**0.448946079**
**SCORE**	0.2135318	0.7904373	0.336232459
**SCENIC**	0.284316	0.6023197	0.386289719
**Harmony**	0.206142	0.9710038	0.340084746
**Combat**	0.1877563	0.9816929	0.315223657
**Raw**	0.2133802	0.280701	0.242454218

Note: Each value in Table 1 was calculated as AUCDF score, the meaning and detail of which were described in the Materials and Methods section of the manuscript.

Section 3. Justification of integrating PPI and ICA in scRNA-seq datasets

In this section, we will discuss whether integrating PPI and ICA makes sense in improving scRNA-seq data analysis. We want to justify the integration from two aspects —'cell clustering' and 'module biological significance'. In a word, we need to answer the following two questions:

1. Is cell clustering improved when incorporating PPI information to ICA?

2. Is the prediction of biologically significant module improved when incorporating PPI information to ICA?

To evaluate whether incorporation of PPI information would aid the cell clustering performance using ICA, we compared ARI value before and after incorporation of PPI in three independent scRNA-seq datasets. We tested three models, two of which are without PPI (model 1 and 2) and one is with PPI (model 3). The only difference between model 1 and 2 is using distinct gene sets for calculating the clustering. Model 1 uses 'activated genes' as co-expression modules and defined module activity through AUCell for cell clustering. Model 2 uses activated genes predicted by ICA directly, and regards them as variable features to perform cell clustering. Model 3 incorporates PPI information with ICA for module prediction, that is, ICAnet. We used Louvain clustering algorithm with shortest nearest neighbor graph and tuned parameter '*resolution*' to maximize clustering performance of each method (7). From the clustering results (see Figure 4a below), we can see that incorporating PPI information could increase the performance of cell clustering (reflected by higher ARI values) in mouse brain scRNA-seq data (8). The conclusion remains the same using mouse blood cell development scRNA-seq data (9) (Figure 4b) and human cell line scRNA-seq dataset (10) (Figure 4c). These results indicate that incorporating PPI information with ICA improves cell clustering performance.

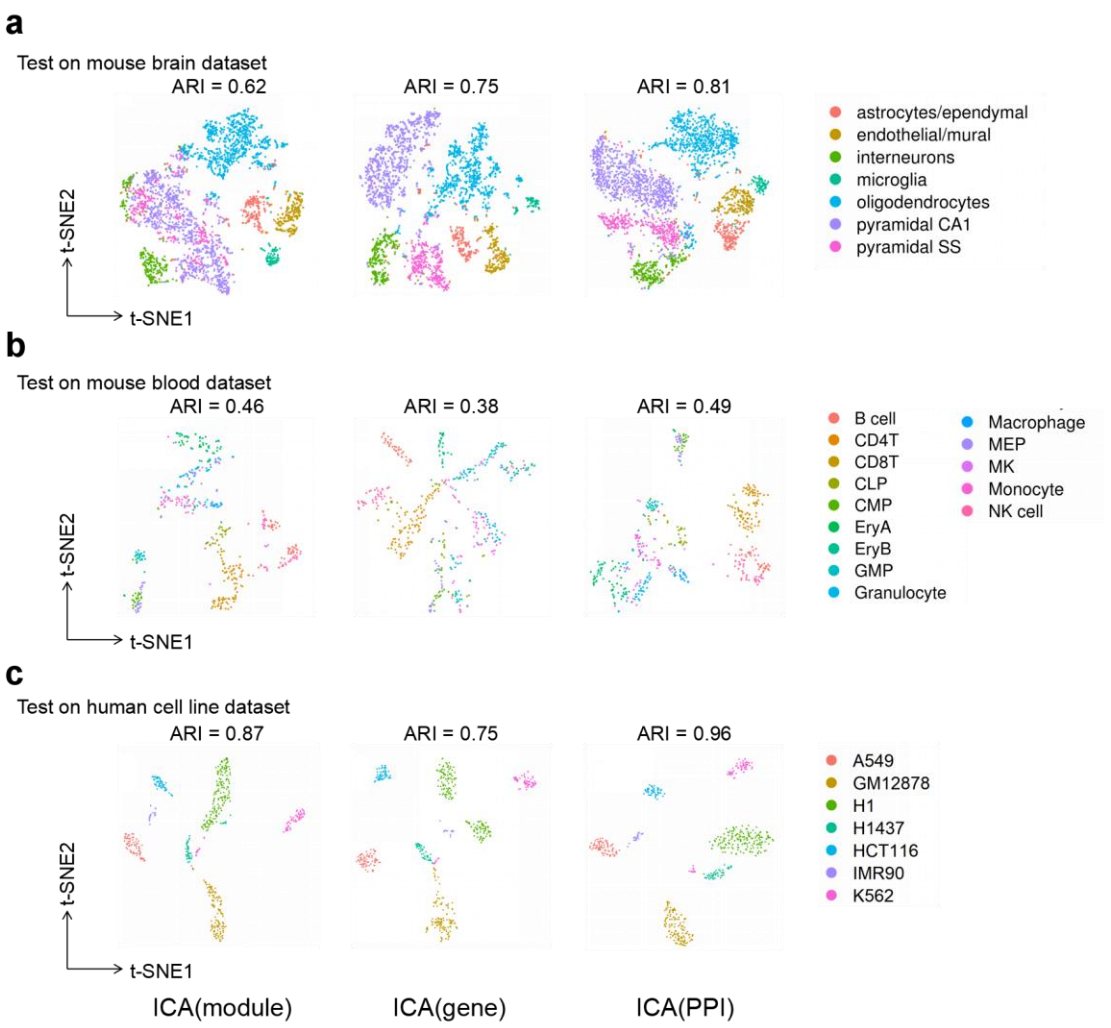

Figure 4. Clustering performance comparison with and without incorporation of PPI information to ICA.

(a-c) Clustering performance comparison among three different models applied on scRNA-seq datasets derived from mouse brain (a), mouse blood cell development (b) and human cell lines (c). Model 1 or ICA(module): Using ‘activated genes’ over each ICA-components as a module, then performing clustering; Model 2 or ICA(gene): Pooling all ‘activated genes’ as the variable genes, then using these genes to perform clustering; Model 3 or ICA(PPI): Integrating ICA-components with PPI network to infer the ‘activated modules’, then using these modules to perform clustering (known as ICAnet). The clustering performance was presented with t-SNE plot, and evaluated with adjusted rand index (ARI) values. Each dot color corresponds to a cell type, as indicated at the right of each panel.

Because PPI has topology information, we further validated the importance of network topology information to cell clustering. Two different ways to alter the topology of weighted PPI were used, which is similar to previously justification strategy of scRNA-seq data and PPI network integration (11). 1) We constructed 100 random networks by keeping the same graph structure of the real PPI, but shuffling the gene names. Thus, these random networks share most of the characteristics of the real network (degree distribution, community structure), except for the true identity of the nodes. 2) We shuffled the ICA score distribution of PPI nodes, then used the shuffled ICA scores to run ICAnet. This process could construct weighted interaction networks with different weight scheme, and it would influence the downstream detection of gene interaction community (based on walk-trapping algorithm). Using these two network perturbation strategies, we evaluated the number of discovered modules and ARI values on two scRNA-seq datasets, one is from mouse brain (8) and the other is from mouse blood cell development (9).

In the first shuffling strategy, we discovered that most of the randomized PPI produced significantly fewer modules (Figure 5a, left; Figure 6a, left; Figure 7a left) and lower ARI values (Figure 5a, right; Figure 6a, right; Figure 7a right) than true PPI in both mouse brain and blood development scRNA-seq datasets, suggesting that breaking the PPI topology influences the co-expression PPI module identification and ultimately affects the cell clustering performance. In the second shuffling strategy, we also observed very similar phenomenon (Figure 5b, Figure 6b, Figure 7b), suggesting that most modules were not generated merely from PPI topology, but from the combination of both PPI and gene co-expression. To examine whether this result could extend to other PPI types, we used MIPPIE (12) PPI network and mouse brain scRNA-seq dataset to repeat the analysis above, and the conclusion still holds (Figure 8a,b).

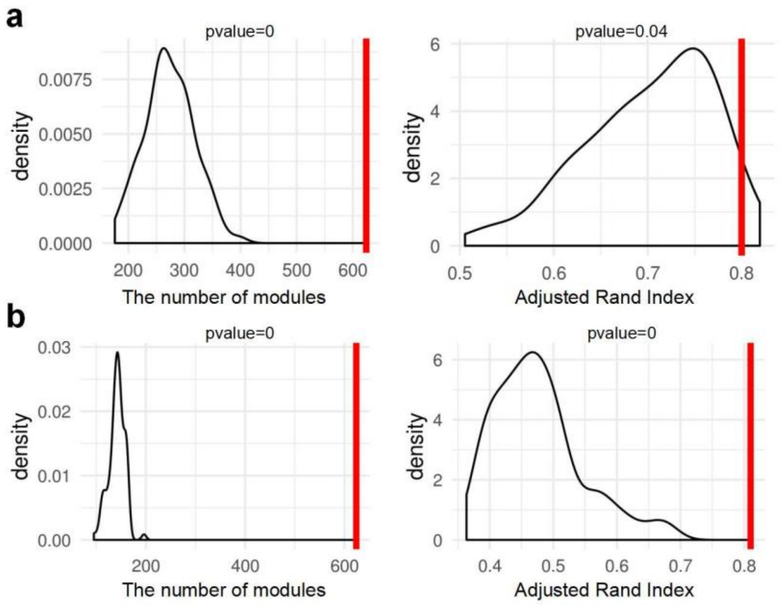

Figure 5. Permutation test on randomized PPI networks to evaluate the impact of PPI information on module discovery and clustering performance using mouse brain scRNA-seq data.

(a-b) Density plot of module numbers (left) and ARI values (right) of 100 runs of ICAnet on randomized PPI networks using two different shuffling strategies (a, keeping the same topological structure of the real PPI but shuffling the gene names; b, shuffling the ICA score distribution on nodes of PPI) in mouse brain scRNA-seq data. The red lines indicate the number of module (left) and ARI value (right) based on real PPI network (BioGRID) (13). To facilitate computing, gene expression matrix was decomposed to 10 source components through ICA to perform ICAnet. The publicly available mouse brain scRNA-seq data was used for the evaluation.

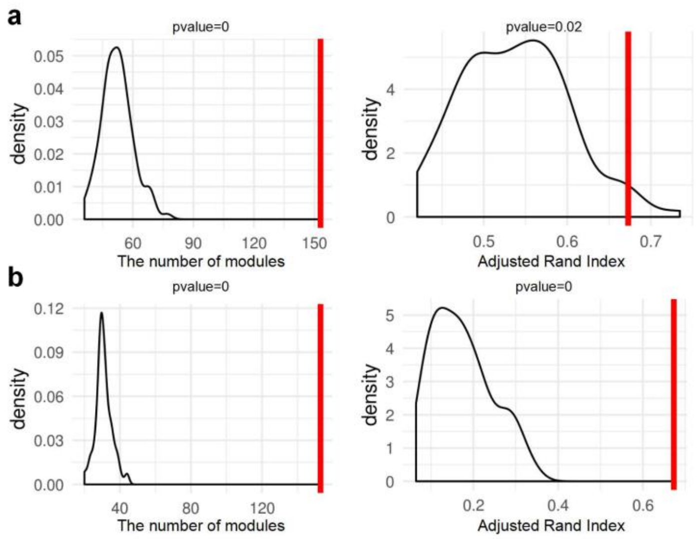

Figure 6. Permutation test on randomized PPI networks to evaluate the impact of PPI information on module discovery and clustering performance using mouse brain scRNA-seq data.

(a-b) Density plot of module numbers (left) and ARI values (right) of 100 runs of ICAnet on randomized PPI networks using two different shuffling strategies (a, keeping the same topological structure of the real PPI but shuffling the gene names; b, shuffling the ICA score distribution on nodes of PPI) in mouse brain scRNA-seq data. The red lines indicate the number of module (left) and ARI value (right) based on real PPI network. To facilitate computing, gene expression matrix was decomposed 10 source components through ICA to perform ICAnet. Meanwhile, for STRING network (14), CIS > 950 was used to filter for high quality PPI network, which resulted in 4,830 protein coding genes and 42,868 interactions. The publicly available mouse brain scRNA-seq data was used for the evaluation.

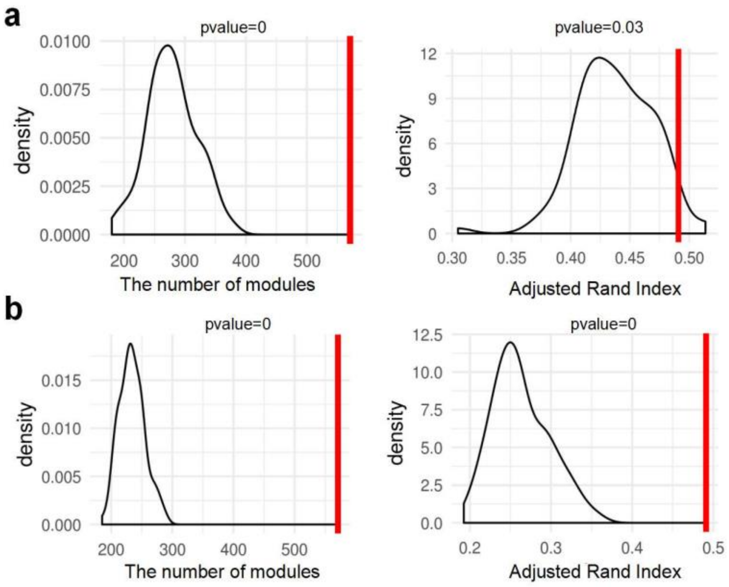

Figure 7. Permutation test on randomized PPI networks to evaluate the impact of PPI information on module discovery and clustering performance using mouse blood cell development scRNA-seq data.

(a-b) Density plot of module numbers (left) and ARI values (right) of 100 runs of ICAnet on randomized PPI networks using two different shuffling strategies (a, keeping the same topological structure of the real PPI but shuffling the gene names; b, shuffling the ICA score distribution on nodes of PPI) in mouse blood cell development scRNA-seq data. The red lines indicate the number of module (left) and ARI value (right) based on real PPI network (STRING). We found that applying BioGRID network only called 30 modules due to the relatively small number of useful PPI information (3,664 PCG genes, 17,606 interactions). Therefore we only tested STRING network on this dataset and use CIS > 600 to filter out low quality interactions. The publicly available scRNA-seq data derived from mouse blood cell spanning multiple developmental stage was used for the evaluation.

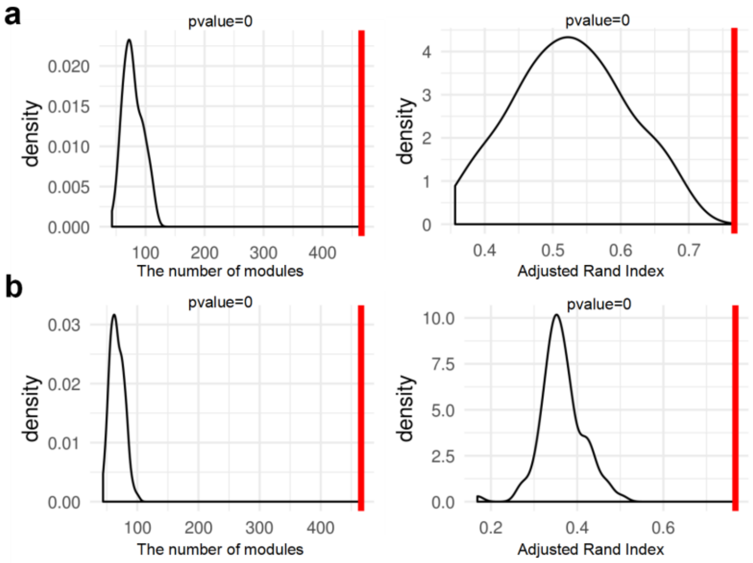

Figure 8. Permutation test on randomized PPI networks to evaluate the impact of PPI information on module discovery and clustering performance using mouse brain scRNA-seq data.

(a-b) Density plot of module numbers (left) and ARI values (right) of 100 runs of ICAnet on randomized PPI networks using two different shuffling strategies (a, keeping the same topological structure of the real PPI but shuffling the gene names; b, shuffling the ICA score distribution on nodes of PPI) in mouse brain scRNA-seq data. The red lines indicate the number of module (left) and ARI value (right) based on real PPI network (MIPPIE). The publicly available mouse brain scRNA-seq data was used for the evaluation.

Section 4. Evaluation of biological significance for predicted modules

One aspect that needs to be justified is that whether the PPI network also influences the biological significance of inferred modules. First, we need to find a way to evaluate the biological significance of each module predicted by ICAnet. Previous researches have given several ways to evaluate the biological significance of gene modules. For example, Tenschendorff et al generated a comprehensive list of breast cancer pathways (including 536 pathways) and used hypergeometric test to assess the enrichment of each computational module pathway (15). Wu et al also used a set of

gold standard protein complexes as references to evaluate the biological significance of predicted modules (16).

Here, inspired by these previous works, we used hypergeometric test method to evaluate the module biological significance. Assuming we have tissue-associated functional pathway sets (we name them as golden pathway sets, which refer to the pathways strongly associated with cell/tissue identity and/or tissue-specific functions), then if the genes in an ICAnet-inferred module were significantly enriched in a golden pathway, the module was considered as biologically significant.

Therefore, we calculate biological significance for each module with the formula as below:

$$\text{module}^{i}_{BiologicalSignificance} = \min\{AdjustPvalue_{hypergeomic}\left(module^{i}, a\right), a \in A\}$$

Simply speaking, we measured the biological significance for module i based on whether the genes involved in were significantly enriched in one of the pathways in pre-defined pathway sets (all pathways recorded in MsigDB (17) and Rectome (18) databases). ‘A’ denotes the golden pathway sets while ‘a’ denotes module genes enriched pathway(s). We measured the enrichment degree based on p value calculated through hypergeometric test, which was frequently used in GO/KEGG enrichment analysis. Correction for multiple testing was done using the Benjamini-Hochberg procedure to minimize the false discovery rate.

It is worth noting that defining the tissue-associated pathway sets (or golden pathway sets) is the key for the whole analysis. Previous researches used manual curation of literatures to obtain tissue-specific and functional relevant pathway sets one-by-one, which is very time-consuming, so we applied a more efficient way to define the golden pathway sets as shown below (Figure 9). We first downloaded independent single cell RNA-seq dataset from the same tissue of benchmark scRNA-seq datasets (mouse brain scRNA-seq and mouse blood cell development scRNA-seq), then we split these scRNA-seq datasets according to their sample/batch labels. Suppose the independent scRNA-seq dataset has n batches, we randomly selected n-1 batches of scRNA-seq dataset as the training datasets, and the remaining one batch as the test data. After

that, we selected top K highly variable genes (HVGs) according to the coefficient of variations of each gene. These HVGs were used to filter the pre-defined pathways (all pathways from MsigDB and Rectome databases). By doing that, we could identify tissue-associated functional pathways. The pathway filtering step was then performed through testing whether these pathways are significantly enriched with HVGs. Next, we used test dataset and independent bulk RNA-seq dataset to evaluate the final pathways to further confirm that these pathways are specific to the tissues in high confidence. Finally, we used the final pathways to measure the biological significance of ICAnet-inferred modules acquired from benchmark scRNA-seq datasets.

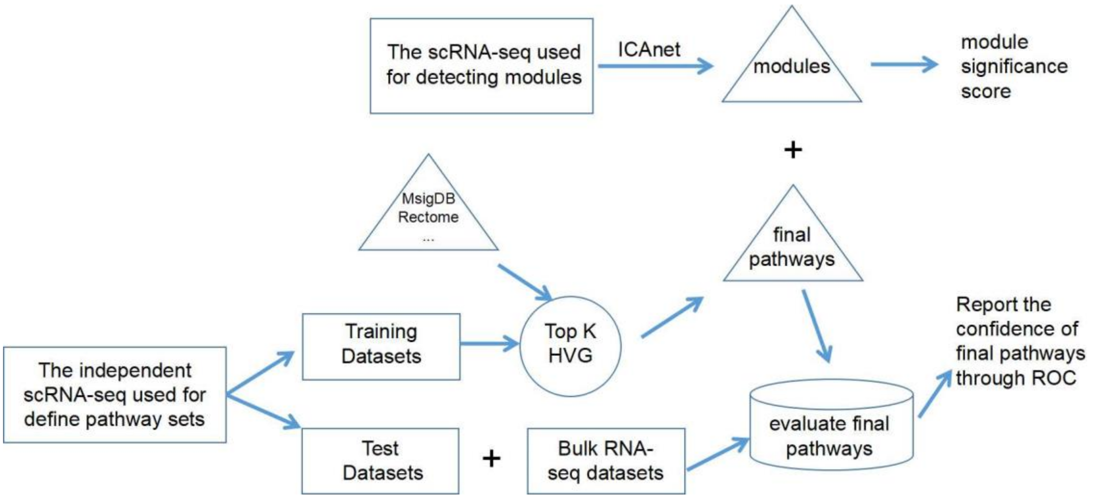

Figure 9. The flowchart to perform module biological significance test.

We evaluated module biological significance through above analysis pipeline. Firstly, we separated downloaded single cell RNA-seq dataset into two parts, the training dataset and test dataset. Then, through calculating Top K HVGs of training dataset, we filtered pre-defined pathways (from the comprehensive pathway database like MsigDB, Rectome) through testing whether these pathways were significantly enriched with the Top K HVGs, and these pathways were considered as highly correlated with the functions of given tissue. To further prove these pathways were strongly associated with the test tissues, we used previously defined test datasets and bulk RNA-seq dataset to evaluate the enrichments of final pathways, and indicated the overall enrichments performance through AUROC value. Lastly, we used the final pathways

(golden pathways) to evaluate the biological significance of ICAnet-inferred modules.

We first performed above analysis on mouse brain systems by downloading independent scRNA-seq dataset (19) (GSE129788) from GEO database, which includes eight young mice (2-3 month) brain. To speed up the computation, we selected four samples of them, and used “YX1L”, “YX2L”, “YX3R” as training datasets, and used “YX4R” as test dataset. We selected top 8000 high variable genes (HVG) from each sample in training datasets, and used the overlapped gene sets among these set as the final HVG. The bulk RNA-seq dataset was downloaded from GEO:GSE148075 (20), and this dataset included 86 bulk RNA-seq samples from three mouse brain regions (hypothalamus, prefrontal cortex and hippocampus). The pre-defined pathways were download from two pathway database, MsigDB and Reactome, both were download from http://download.baderlab.org/EM_Genesets/current_release/Mouse/symbol/Pathways/.

To evaluate final tissue-specific pathways, we selected the top 3000 (more stringent than top 8000) HVG for each test dataset, and calculated the enrichment p value in HVG for all pathways through hypergeometric test. After log10 transformation of the p value, we used ROC to visualize how well the final pathway was enriched, as indicated by -log10(pvalue) score. The result showed that final pathways were enriched with HVG in independent datasets (AUC on scRNA-seq: 0.94, AUC on bulk RNA-seq: 0.86, see Figure 10 below). To further validate its confidence, we performed permutation tests through randomly sampling the same number of pathway sets from initial pathway sets as that of the final pathway sets and calculated their AUC. We repeated this step for 100 times so that we could generate a null distribution of AUC value which indicate pathway sets enrichment on independent gene sets (HVGs). The tests were significant on both datasets (permutation test p value = 0).

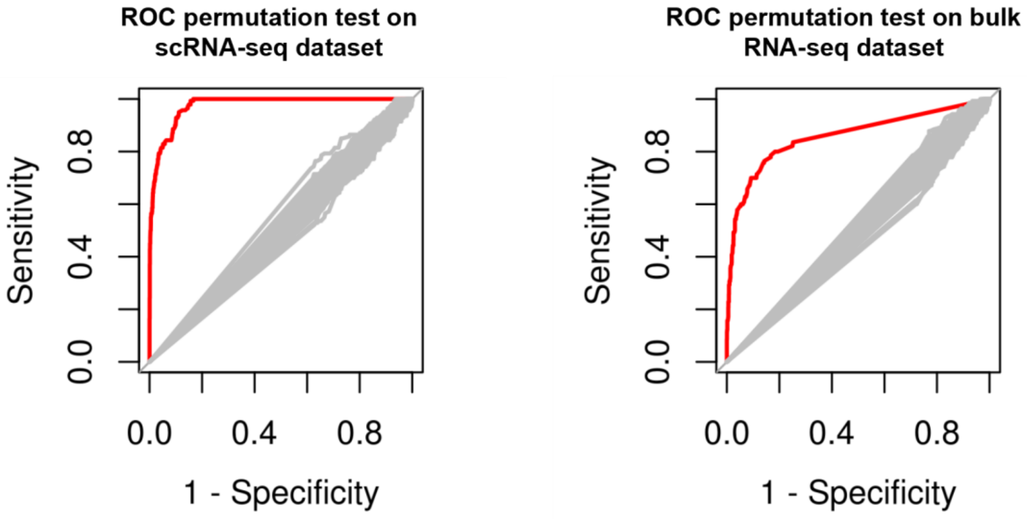

Figure 10. Confidence validations of the golden pathway sets in independent scRNA-seq and bulk RNA-seq dataset

We validated the confidence of our final (golden) pathway sets on independent scRNA-seq (left panel) and bulk RNA-seq gene sets (right panel) through ROC analysis. The high AUC (0.94, left panel, red curve; 0.86, right panel, red curve) indicated the enrichment of final pathway sets on independent datasets. We also sampled random pathway sets from initial pathway sets with the same number of pathways and test their enrichment. The result showed that the randomly sampled pathway sets is poorly enriched in independent dataset (AUC around 0.5, grey curves in Figure 10).

To further validate that the PPI topology is important for the biological significance of resulted module, we used the same method mentioned in the response to Q2 to randomize PPI structures or weights of PPI network, then make comparison of biological significance between modules learned from real PPI and those from randomized PPI. We found that in both randomized manners, the modules learned from real PPI network presented much better biological significance (smaller FDR) compared with permutated network in mouse brain (Figure 11) scRNA-seq data.

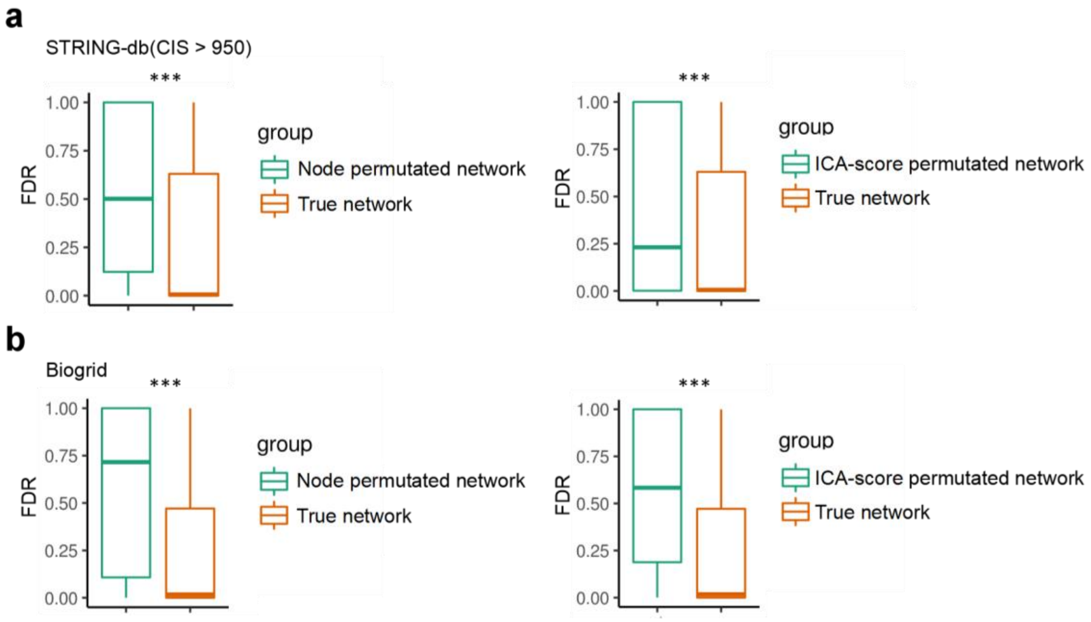

Figure 11. Comparison of module biological significance derived from real network and randomized networks in mouse brain scRNA-seq data.

a. Module biological significance comparison between the real gene network and permutated network by randomizing the PPI structure (left panel) or randomizing the weights of PPI (right panel). The mouse brain scRNA-seq data (GSE60361) and STRING PPI networks were used for the analyses. ***, Wilcoxon test p value < 0.01.

b. Module biological significance comparison between the real gene network and permutated network by randomizing the PPI structure (left panel) or randomizing the weights of PPI (right panel). The mouse brain scRNA-seq data (GSE60361) and BioGRID PPI networks were used for the analyses. ***, Wilcoxon test p value < 0.01.

To validate the result was not caused by dataset specific artifacts, we moved on to scRNA-seq dataset of mouse blood systems (9). Unfortunately, we could not find an independent bulk RNA-seq dataset with sufficient sample size. Instead, we collected 121 pathways associated to blood cell development through manual curation of literatures and databases as the golden pathways to go on with the following analysis. Again, the results validated that analysis based on the real gene networks discovered more biologically significant modules than those based on either node permutated (Figure 12 left) or ICA-score permutated gene network (Figure 12, right). These results

support the notion that topology of PPI could greatly affect the biological significance of ICAnet identified modules.

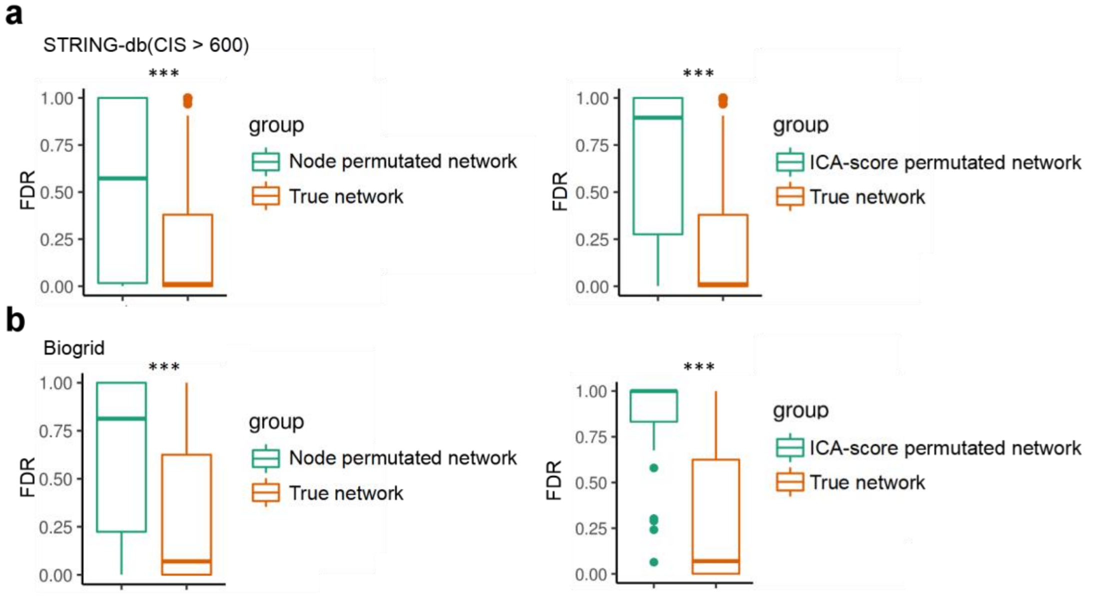

Figure 12. Comparison of module biological significance derived from real network and randomized network in mouse blood development scRNA-seq dataset.

a. False discovery rate (FDR) comparison between the real gene network and permutated networks by randomizing the PPI structure (left panel) and randomizing the weights of PPI (right panel). The mouse blood cell development scRNA-seq datasets (GSE116530) and the manually curated pathways collected from literatures (as the golden pathways) were used in the analyses. STRING PPI is used for analysis. CIS score threshold 600 is chosen. ***, p value < 0.01 based on Wilcoxon rank sum test.

b. False discovery rate (FDR) comparison between the real gene network and permutated network by randomizing the PPI structure (left panel) and randomizing the weights of PPI (right panel). The mouse blood cell development scRNA-seq datasets (GSE116530) and the manually curated pathways collected from literatures (as the golden pathways) were used in the analyses. BioGrid PPI is used for analysis. ***, Wilcoxon rank sum test p value < 0.01.

In summary, we compared the clustering performance by using three models to justify the incorporation of PPI information to ICA in improving the cell clustering. We also used permutation test to validate the importance of weighted PPI topology for both cell clustering and module biological significance. The results showed that breaking the raw topology of interactions would limit not only the cell clustering performance, but also the number and biological significance of detectable modules.

Section 5. Evaluation of statistical significance for predicted modules

Another crucial point is about how to evaluate the statistical significance of ICAnet-predicted modules. As the modules detected merely based on the topology structure (such as more edges) should have poor co-expression than modules detected based on both PPI and co-expression, we can use module co-expression information to discriminate these two module types. To do this, we used matrix conditional number to assess the co-expression of a set of genes. Supposing we have a module inferred by ICAnet, we extracted the expression profile of corresponding genes from normalized gene expression profile. After Z-score transformation for each gene, we measured the degree of 'co-expression' through calculating the matrix conditional number, which was defined as the maximum singular values of a matrix divided by the minimum singular values of the same matrix. To assess the statistical significance, we generated the null distributions through randomly selecting the same number of genes from ICA generated 'activated gene sets', then we calculated their matrix conditional number, and plotted the empirical distributions of matrix conditional number of the randomized modules of the same size. If the 'activated module' is derived merely from the topology structure of the PPI networks, the genes in the module shall have relatively poor co-expression patterns, close to the null distribution generated from background (ICA generated activated gene sets). Otherwise, if the 'activated module' is detected based on both PPI and co-expression information, the module co-expression should be statistically away from null distribution of the background. Therefore, each module has a p value to indicate its statistical significance.

To evaluate the above strategy, we want to analyze whether the number or ratio of significant modules generated from real network is significantly higher than those from random networks. We firstly tested the statistical significance of each module inferred from blood cell development scRNA-seq dataset and PPI network from STRING-db. Considering computing all ICA components is extremely time-consuming, we only selected the top 10 ICA-components for analysis. The result showed that there were 14.8% of modules (55 modules) passed the significance threshold (Figure 13, left panel, p value < 0.05). For comparison, we randomized PPI for 100 times, and measured the statistical significance of these modules. We found that the number or ratio of significant modules was significantly smaller than the original results based on true networks (Figure 13, right panel, with p value = 0). These above results suggest that our statistical test method could tell the difference between real and random network.

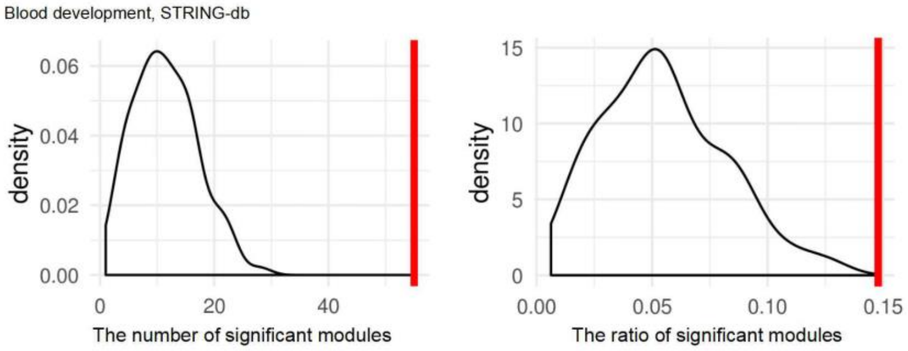

Figure 13. Evaluation of the statistical test method.

The density plots indicate the null distribution of the number (left) and ratio (right) of significant modules derived from mouse blood cell development scRNA-seq data (GSE116530). The red lines indicate the number (left) or ratio (right) of significant modules calculated from true PPI networks. STRING PPI database was used for the analysis.

We hypothesized that using this method to control module statistical significance could increase the biological pathway enrichment. To validate this hypothesis, we evaluated the method through measuring their prediction precision and recall rate of

relevant biological pathways (16). We assume that P and B are the sets of genes predicted by a computational method (such as ICAnet, SCORE) and real ones (golden pathways) in the benchmark, respectively. Ncp is the number of correct predictions which were enriched at least with a biological pathway and Ncb is the number of recalled biological pathways which were enriched with a predicted module. Precision and recall rate are defined as follows:

$$N_{cp} = \left|\{p|p \in P, \exists b \in B, FDR_{hypergeometric}(p,b) < 0.05\}\right|$$

$$N_{cp} = \left|\{b|b \in B, \exists p \in P, FDR_{hypergeometric}(p,b) < 0.05\}\right|$$

$$\text{precision} = \frac{N_{cp}}{|P|}$$

$$\text{recall} = \frac{N_{cb}}{|B|}$$

We ran ICAnet with two different ICA implement methods (JADE and fastICA) and ran SCORE with three 'correlation metric' (cor, phi, phs) in mouse brain scRNA-seq datasets for comparison of prediction precision and recall rate. Further, we also performed module quality (statistical significance) control (MQC) through filtering out ICAnet modules which had a tested p value > 0.05. The results showed that after MQC, the precision was increased compared with the method without MQC, while the recall rate was slightly decreased (Figure 15), suggesting that although MQC could improve the enrichment of the biologically significant modules, it may miss some of the pathways whose modules didn't pass the statistical significance threshold (p value < 0.05). We also found that using ICA combined with PPI strategy to infer modules showed relatively higher precision compared with correlation-based methods (Figure 14,15, left). To further support the conclusion, we used the data from mouse blood cell development and pathway sets collected from literature search to repeat the analysis, which resulted in similar results (Figure 16). To validate the prediction precision improvement of MQC is not caused by random effects, we randomly selected the same number of modules and repeated for 100 times in both mouse brain dataset and mouse blood development dataset, and calculated the prediction precision. The result showed

that the prediction precision improved significantly for MQC modules compared to the randomly selected ones (Figure 17).

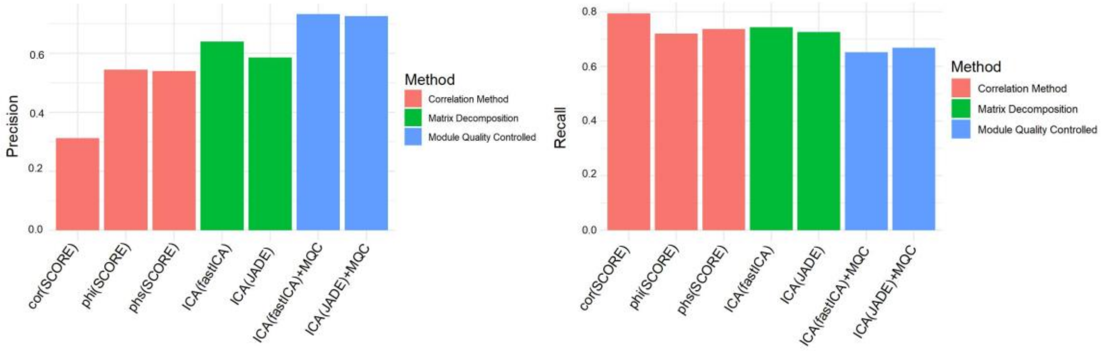

Figure 14. Comparisons of the ability to infer biologically significant modules of different module-prediction methods in mouse brain scRNA-seq data.

Seven module identification methods with different strategies or parameters were compared based on the same mouse brain scRNA-seq dataset. Prediction precision (left panel) and recall rate (right panel) were used for the performance evaluation. cor: Pearson correlation; phi, phs: two different proportionality metrics; MQC: module quality controlled by filtering out the modules with statistical significance > 0.05. STRING PPI database was used for the analysis.

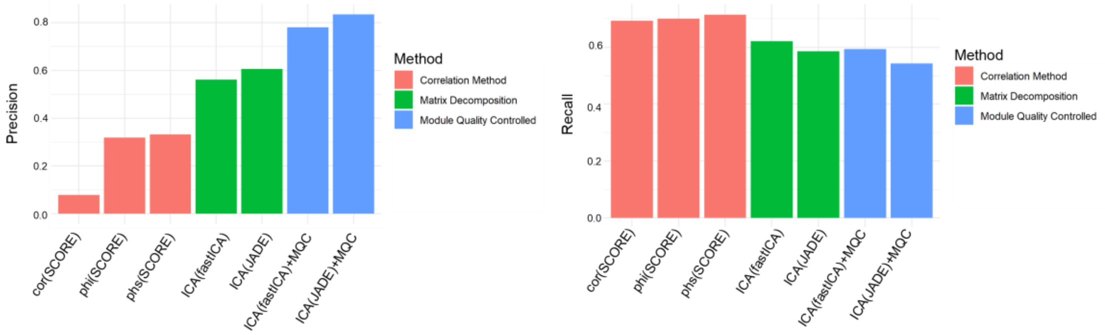

Figure 15. Comparisons of the ability to infer biologically significant modules of different module-prediction methods in mouse brain scRNA-seq data.

Seven module identification methods with different strategies or parameters were compared based on the same mouse brain scRNA-seq dataset. Prediction precision (left panel) and recall rate (right panel) were used for the performance evaluation. cor:

Pearson correlation; phi and phs: two different proportionality metrics; MQC: module quality controlled by filtering out modules with statistical significance > 0.05. BioGrid PPI database was used for the analysis.

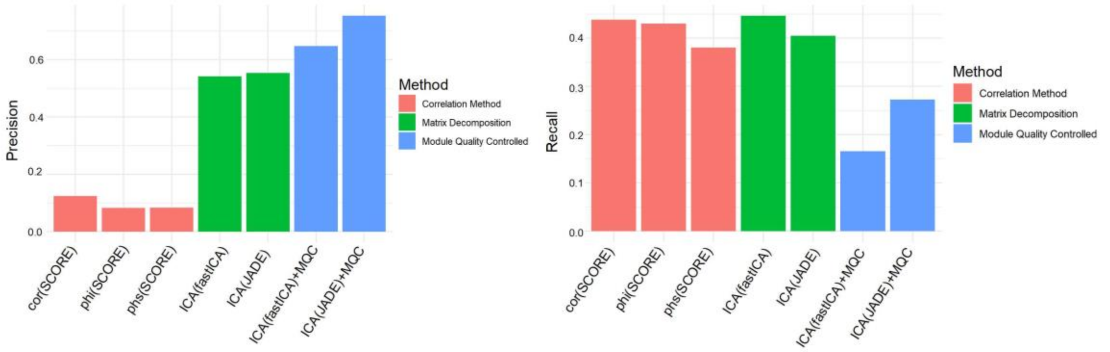

Figure 16. Comparisons of the ability to infer biological significant modules of different module-prediction methods in mouse blood cell development scRNA-seq data.

Seven module identification methods with different strategies or parameters were compared based on the mouse blood cell development dataset. Prediction precision (left panel) and recall rate (right panel) were used for the performance evaluation. cor: Pearson correlation; phi, phs, rho: three different proportionality metrics; MQC: module quality controlled by filtering out modules with statistical significance > 0.05. STRING PPI database was used for the analysis.

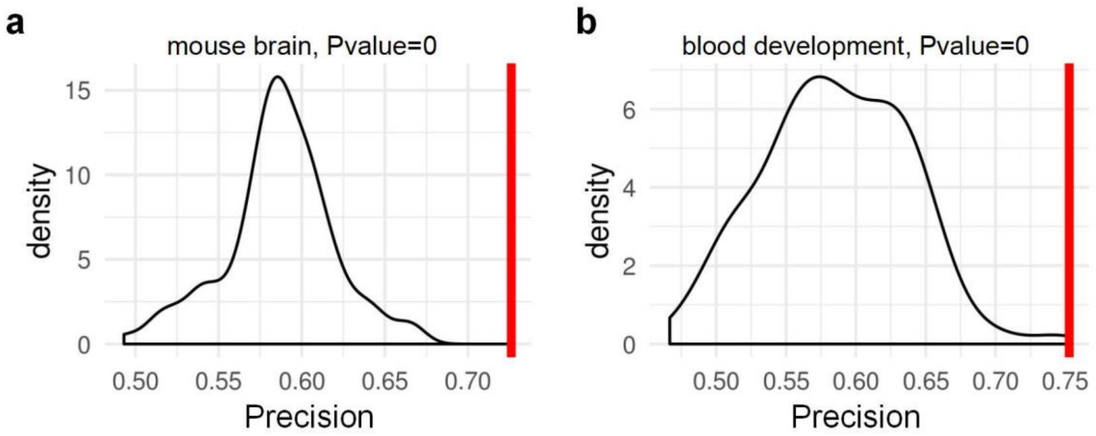

Figure 17. Validation of the MQC improvement in mouse brain dataset and blood development scRNA-seq datasets.

The density plots of null distribution of precision values in mouse brain (a) and blood development (b) scRNA-seq datasets. The red line indicates the precision values acquired based on MQC-filtered modules.

These results indicated that controlling the module statistical significance could improve the enrichments of biologically significant modules, although it might increase the number of ‘false negative’ modules (as reflect by the decrease of recall value in Figure 14,15,16 right panel). Thus statistical significance of predicted modules would help with the biological interpretation while using all modules may benefit cell clustering via preserving all biological information. Therefore, in the application of ICAnet, we recommend to follow the strategy of using all ICAnet-predicted modules for cell clustering and using the statistical significance (p-value) of each module to select the representative modules for biological interpretation.

Section 6. Incorporating TF-regulons into ICAnet (ICAnetTF)

Processing TF-motif database to generate TF-gene networks: We downloaded the TF-binding information based on the annotation databases provided by RcisTarget (21), which includes two types of dataset: 1. Gene-motif ranking: this database provides the ranking score of all the genes for each motif. It gives rich information of possible genes’ motif and quantifies the probability of motif existence through a score. 2. motif-to-TF annotation: it provides motif annotations of transcription factors. We used information of these two datasets to generate TF-gene regulation matrix. The gene-motif ranking dataset provided by RcisTarget containing the genes, the potential regulators and their ‘association measures’, which represents the ranking score of all the genes for each TF-binding motif. We tried several ways to determine the score threshold so that we could transform the annotation dataset into binary motif-gene networks, and finally we decided to use a similar way provide by SCENIC: (i) Taking the genes with a ranking score larger than i times of the standard deviation (of ranking score) from the mean for each motif; (ii) Taking the TFs with the ranking score larger than i times of the standard

deviation from the mean for each gene. For the mouse dataset, we set i as 1.2, and for human dataset, we set i as 1. Then, according to the TF-motif annotation, we assigned target genes for each transcriptional factor. This processing workflow could control the quality of TF-gene network.

TF-regulons enrichment test on ICA-components through Monte Carlo: To integrate TF-regulons with ICA-components, we used Monte Carlo method to perform TF-target enrichment test on each component. The regulatory activity of each TF was measured through calculating the sum of ICA score of its target genes (the attributes value on source components). Further, we randomly selected genes with the same number as the TF-targets, and also calculated the sum of their ICA scores. We repeated this step for *number of Monte Carlo* (nMC) times to get the null distribution (In this research, we set the nMC to 100). A TF whose ICA score sum of its target genes is significantly higher (or lower) than that of the randomly sampling genes (evaluated by inspecting their empirical p values) was considered as the regulatory TF involved in corresponding expression regulation. The resulted TF-regulon would be identified as a module, and was used for downstream clustering analysis.

Section 7. Integrating PPI from different species

It is necessary to justify if ICAnet could extend its good performance to other type of biological systems. Therefore, we downloaded a previously un-used scRNA-seq dataset from *Drosophila melanogaster* (serving as an example) to verify whether ICAnet can incorporate PPI network from different species (https://data.mendeley.com/datasets/wtm6sygnmg/3). We also downloaded corresponding PPI network (*Drosophila melanogaster*) through the function getPPI_String provided by RSCORE. The scRNA-seq dataset is a comprehensive cell atlas of the adult *Drosophila* ovary that contains transcriptional profiles for every major cell type in the ovary. As this dataset contains three different batches, we used this dataset to validate whether ICAnet could largely remove the batch effect, and re-

construct the majority of cell states that the original authors had found. The results are shown in the Figure 18 and 19 below.

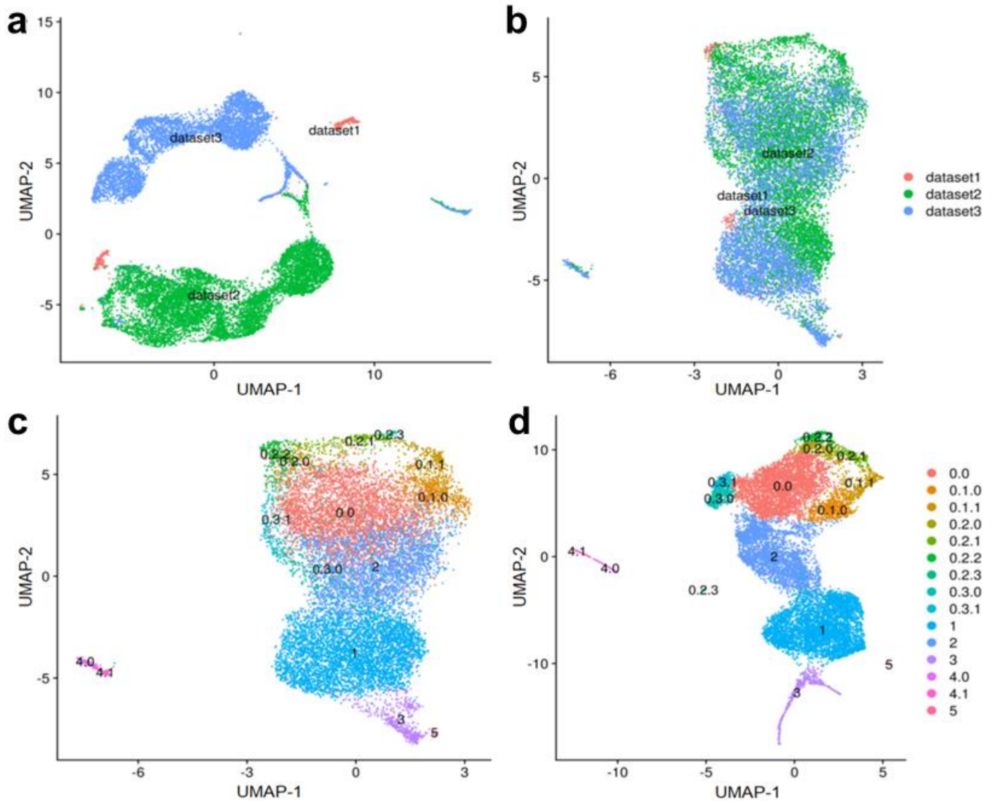

Figure 18. ICAnet could largely remove the batch effect in *Drosophila melanogaster* scRNA-seq datasets with *Drosophila*-specific PPI.

(a) UMAP visualization of the original scRNA-seq dataset (without ICAnet). Each dot represents a cell, and the color represents the three batches (dataset 1-3). (b) UMAP visualization of the ICAnet-processed dataset, and each dot-color represents a batch. (c) UMAP visualization of the ICAnet-processed dataset. Each dot-color represents a cell cluster annotated by the original authors, with the cell clusters denoted by the numbers inside. (d) UMAP visualization given by original authors, wherein the batch effect is corrected by Seurat V3(CCA). Each number represents a cell cluster.

As from the performance comparison shown in Figure 18 above, we can see that ICAnet could largely remove the batch effect (Figure 18a,b), and reconstruct the

majority of cell states in *Drosophila melanogaster* scRNA-seq dataset with *Drosophila*-specific PPI (Figure 18c,d). Also, the relative cell state coordinates are close to those in the original results which is presented through Seurat CCA correction (Figure 18c,d). Meanwhile, we also noticed that some of the cell states may not so clear (e.g. 0.3.0), possibly due to the limited number of protein-protein interactions from *Drosophila* that restricts the cluster granularity in the cell similarity graphs. Therefore, when applying ICAnet on different species, it is more appreciate if the number of PPIs is sufficiently enough. Considering that there has extensive computational prediction/experimental validation of PPIs in model organisms, we recomand to use ICAnet on scRNA-seq datasets generated from model organisms (e.g., human, mouse, and fruit fly).

Meanwhile, we also tested whether ICAnet could use PPI network comes from different species when analyzing the scRNA-seq data of *Drosophila melanogaster*. We applied human PPI network and subsitituted the associated genes with their orthologous *Drosophila melanogaster* genes, and then performed ICAnet. It showed that result based on *Drosophila melanogaster* scRNA-seq data and human PPI (Figure 19) is not comparable with the result based on *Drosophila* scRNA-seq data and *Drosophila* PPI (Figure 18). These results suggest that it is more proper and of more sense to use species-specific PPI information for ICAnet. Such recommendation was also noted in the GitHub tutorial.

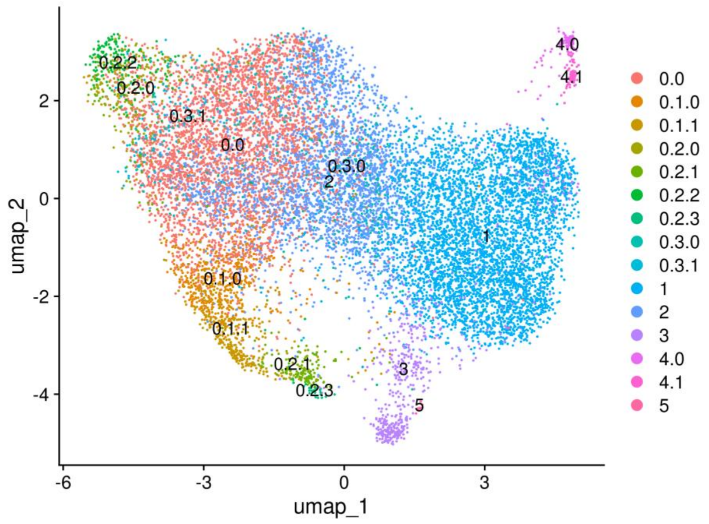

Figure 19. Using ICAnet on *Drosophila melanogaster* scRNA-seq dataset with human PPI network. The number in each color refers to a cell cluster.

Section 8. Dimensionality reduction methods used in ICAnet

Dimensionality reduction of module activity matrix: To reduce the dimension of module activity matrix for clustering and data visualization, ICAnet provided three alternative methods for Dimensionality reduction:

1) PCA: Followed by original single cell analysis workflow, ICAnet can directly use PCA to select a number of principle components for Dimensionality reduction.

2) SVD: ICAnet first centralizes each column of module activity matrix, then uses L2 normalization so that each cell module activity distribution is comparable. Next, ICAnet applies Singular Value Decomposition (SVD) on normalized module activity matrix, and SVD can decompose the matrix into three matrix, $M = U\sum V^T$, in which M represents module activity matrix, U represents left singular vector matrix, $\sum$ represents diagonal singular value matrix, and V represents right singular vector matrix. Each singular value represents the data variation in corresponding direction, so that we can add a transformation function to control the signal smoothness on spectrum.

$$\sum_{ii}^{*} = (\sum_{ii})^{p}$$

This power function can filter out noise signal of the data, in which the signal of noise direction (the corresponding singular value is relatively small) can be relatively reduced. This process is intuitively similar to low pass filtering in Fourier transformation. Finally, the cell embedding vector can be derived as follow:

$$V^{*} = V\sum^{*}$$

3) In previous research, AUCell (21) provides a function named as AUCell_exploreThresholds to select optimal cut-off to transform continual AUC activities into binarized values for each module (if module activity is higher than threshold in the given cell = 1, otherwise =0). The binary activity matrix tends to highlight higher order similarities across cells, therefore can be used to remove technique noise and batch effects.

Section 9. Using ICAnet to identify cell differentiation trajectory on mouse whole-testis single cell RNA-seq dataset

To demonstrate that ICAnet can reveal developmental trajectory other than blood cells, we applied it to analyze our home-brew 10X Genomics scRNA-seq data from mouse whole testis (see **Section 9** for details), which consists sperm-related cells at all developmental stages. This dataset has two biological replicates and each contains ~2000 cells after data quality control (see **Section 9**). We analyzed these data with ICAnet and Seurat (the raw single cell RNA expression analysis workflow), and found that ICAnet can largely denoise the dataset and group the cells into functional interpretable clusters, as evaluated by expression of classical marker genes (Figure 20 a,b,c). We further compared our cell type annotation of each cluster with previous FACS sorted single cell expression data (22), and found they agreed well with each other regarding reference gene expression (Figure 20d). We next performed pseudotime analysis based on the principle components calculated regarding module activity (23), and found that ICAnet could reduce the noise on pseudotime calculation, as exemplified by *Piwil1*, whose real expression pattern (mid-stage specific expression (24)) was revealed in our dataset by ICAnet but not Seurat (Figure 20e,g). We also found that using SVD-based embedding could enhance the biological signal to help identifying cell-cell transition (see **Section 7** for details) and the results showed that transition relationship predicted by ICAnet agreed well with DDRtree based trajectory analysis, which has been widely used in trajectory tracking (23) (Figure 20f,h), correctly captured the progression from spermatogonia to elongating spermatids (24). These analyses support that ICAnet can recover cell-cell transition relationship in a tissue with mixed cells of different developmental stages, in addition to its ability to infer cell state.

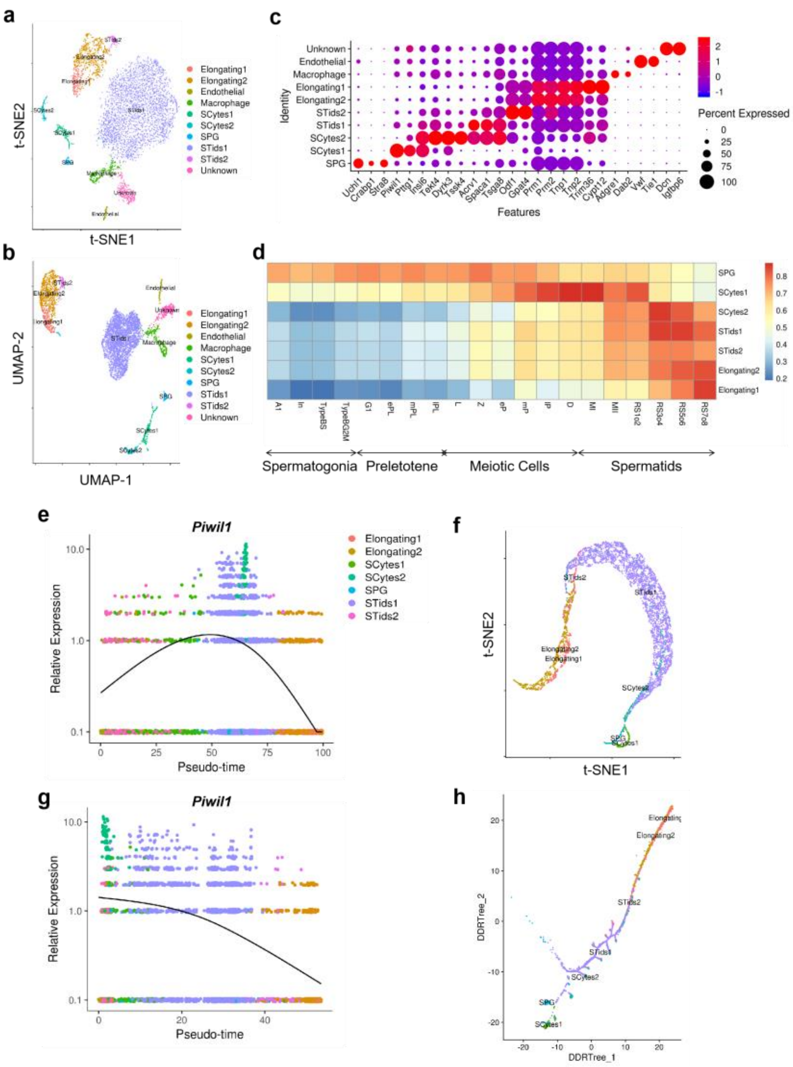

Figure 20. ICAnet can identify cell trajectory structure through SVD embeddings in mouse whole-testis single cell RNA-seq dataset.

a-b. t-SNE (a) and UMAP (b) visualization of ICAnet-inferred cell clusters derived from whole testis scRNA-seq data. Cells are colored by ICAnet defined clusters. Each cluster is annotated with a cell type based on marker gene expression. Each dot represents a cell. SPG, spermatogonia; SCytes, meiotic spermatocytes; STids, round spermatids; Elongating, elongating spermatids.

c. Dotplot displaying the expression levels of representative marker genes of each cluster. Spot size denotes the percentage of cells expressing the marker gene within each cluster and color intensity denotes the expression levels of the gene.

d. Heatmap displaying the cluster expression similarity with the reference scRNA-seq dataset. Each cluster/cell type expression was represented by the average expression

values of the cells belong to the corresponding cluster/cell type. The similarity score (color key) is Spearman correlation coefficient.

e. Log_2CP10K expression changes of *Piwil1* along the pseudo-time order. The pseudo-time is calculated through Monocle with the PCA cell embedding vectors of ICAnet as input.

f. Visualizing cell type differentiation trajectory through ICAnet. The cell embedding vectors required for t-SNE are calculated through SVD-based embedding (see **Section 7**).

g. Log2CP10K expression change of *Piwil1* (Y axis) along the pseudo-time order. The pseudo-time is calculated through Monocle with the PCA cell embedding vectors of the raw gene express matrix. Each dot represents a cell, and the dots are colored according to the cell types.

h. Cell type differential trajectory visualization through DDRtree embedding analysis. Each dot represents a cell colored according to corresponding cell types.

Section 10. Datasets information used in the present study

The information summary is listed in Supplementary Table S1. The detailed information is as below:

Cell line data (10X Genomics)

The cell ranger output files of the cell line experiment were obtained from subfolders containing the raw sequencing data of the ‘293T cells’, ‘Jurkat cells’ and ‘50%:50% Jurkat:293T cell mixture generated by 10x Genomics. The population of each cell type is monoclonal, so we used these datasets to demonstrate that ICAnet can perform batch effect correction perfectly. We used the Read10X function of Seurat package to extract the UMI count table from the Cell Ranger output files, and we defined cell types using labels from the original study. The gene expression data contains 16,602 genes acquired using the 10X Genomics scRNA-seq platform. All datasets can be downloaded from https://support.10xgenomics.com/single-cell-gene-expression/datasets/1.1.0/.

Mouse hematopoietic stem and progenitor cells

The datasets of mouse hematopoietic cells used for this study were from two sources. One is generated by Nestorowa et al (25), who used SMART-seq2 protocol to construct sequencing library of hematopoietic stem and progenitor cells derived from 12-week-old female mice (GSE81682, 1920 cells). The other is generated by Paul et al. (26), who used MARS-seq protocol for library construction of myeloid progenitors from 6 to 8-week-old female mice (GSE72857, 10,368 cells).

Mouse cortex and hippocampus dataset (Zeisel et al.)

Single cell RNA-seq data of 3005 brain cells from juvenile mice (21-31 d old) has been extensively used as benchmark dataset by different methods such as SC3 and SCENIC (3,21). It contains seven major cell types in hippocampus and somatosensory cortex. The expression matrix was download from GEO (GSE60361), which contains the UMI counts for 19,972 genes across 3,005 cells that passed the quality control. To compare ICAnet with other clustering algorithms, we followed the processing workflow given in SCENIC. We filtered out low or no expression genes (the total counts in all cells is less than 90 or counts were detected in less than 30 cells), which finally resulted in 13,063 useful genes in the expression matrix.

Mouse oligodendrocytes (Marques et al.)

The oligodendrocytes dataset generated by Marques et al. contains scRNA-seq data derived from 5,069 oligodendrocyte lineage cells (27). We performed module recovery analysis and clustering analysis on this dataset. The expression matrix was downloaded from GEO (GSE75330), which provides the expression values in UMI counts for 23,556 genes in 5,069 cells. The same analysis workflow as reported by Zeisel et al was used for related analysis.

Human oligodendrocytes (Jakel et al.)

The human oligodendrocyte dataset (generated by Jakel et al. using the 10X Genomics platform) contains single-nucleus RNA sequencing data (snRNA-seq) of 17,799 cells from the white matter of post-mortem tissue of five individuals without neurological disease (as the control) and four individuals with progressive multiple sclerosis (28). This dataset was used to examine whether oligodendrocyte associated network in mouse data can be re-discovered in human oligodendrocyte dataset using ICAnet. We downloaded this dataset from GEO (GSE118257), and only used control samples for analysis. Genes expressed in more than 1% of all cells were kept for further analysis. After that, we performed standard Seurat clustering, and located oligodendrocyte progenitor cells in tSNE space based on tSNE map and previous cell annotation given by the original authors.

scRNA-seq datasets used for benchmark study

In addition to mouse cortex and hippocampus dataset by Zeisel et al., we used five more scRNA-seq datasets for benchmark study. The information of these five datasets are as follow:

Mouse embryonic dataset (Biase et al.): This dataset used deep single-cell RNA-seq of ten 2-cell and five 4-cell mouse embryos . We selected three cell types (2-cell, 4-cell and zygote) for clustering benchmark.

Mouse embryonic dataset (Goolam et al.): Goolam et al. isolated individual cells from successive stages of mouse embryos and assayed transcriptomes of 124 cells from 28 embryos using Smart-Seq2 single-cell RNA-sequencing protocol (29). This dataset contains five cell types including 16-cell, 2-cell, 4-cell, 8-cell and blast cell.

Mouse cerebral cortex dataset (Pollen et al.): Pollen et al. captured and assayed transcriptome of 301 single cells from four tissues including blood, dermal, neural and pluripotent by SMARTer protocol (30).

The above mentioned three datasets and their cell type annotation were downloaded from https://hemberg-lab.github.io/scRNA.seq.datasets/.

Liver cancer dataset (Ma et al.): Ma et al. generated the single-cell transcriptomic landscape of liver cancer biospecimens from 19 patients through droplet based sequencing protocol (31). We downloaded cell type annotation and gene expression data from GEO (GSE125449).

Head and neck cancer dataset (Puram et al.): Puram et al. profiled transcriptomes of ~6,000 single cells from 18 patients with head and neck squamous cell carcinoma (HNSCC) using inDROP protocol (32). We downloaded related cell type annotation and gene expression data from GEO (GSE103322).

Human pancreas datasets (Muraro et al., Baron et al., and Segerstolpe et al)

Muraro et al. assayed the human pancreas islets transcriptomes of 2126 single cells from four deceased organ donors by using CEL-seq (33). Baron et al. implemented inDrop to determine the transcriptomes of over 8,569 individual pancreatic cells from four human donors (34). Segerstolpe et al sequenced pancreatic tissue and cultured islets obtained from six healthy and four T2D donors of various BMI and age through Smart-seq2 (35). We downloaded these three datasets from GEO (GSE84133, GSE85241) and ArrayExp (E-MTAB-5061 and E-MTAB-5060). Genes who expressed in less than 1% of all cells were filtered out. We ran 'randomly' (5) to denoise the data of each single cell. We further took the consensus genes of three denoised dataset as final feature genes, and concatenated three expression matrix as final gene expression matrix.

Mouse hematopoiesis time course dataset (Sala et al.)

This dataset represents the single cell transcriptional profiles from mouse embryos collected at nine sequential time points ranging from 6.5 to 8.5 days post fertilization (36). We kept samples from eight time points (E6.75-E8.5), and eight cell types annotated by original authors. We filtered out genes whose expression counts were smaller than 30. We also normalized the dataset with the size factor calculated by Sala et al. The dataset can be downloaded from the link:

https://github.com/MarioniLab/EmbryoTimecourse2018

Human acute myeloid leukemia dataset (Peter et al.)

The AML dataset by Peter et al. contains multi-omic single cell sequencing data of 38,410 cells from 40 bone marrow aspirates, including 16 AML patients and five healthy donors (6). They applied a machine learning classifier, which combines genotype and expression data, to distinguish the spectrum of malignant cell types. To validate that ICAnet can correct tumor batch effects, we merely selected scRNA-seq data of 16 AML patients (Day 0). These datasets were downloaded from GEO (GSE116256). Based on their machine learning classifier results, we sampled the malignant cells and merged all batches together to create a large gene expression matrix of 27,899 genes and 11,641 cells. We filtered out the genes expressed in less than 1% of all cells. Further, we filtered out genes related to ribosome and mitochondrion to prevent their disturbance on clustering results. The human ribosome related gene list was downloaded from website http://ribosome.med.miyazaki-u.ac.jp/.

Bulk RNA-seq dataset used for AML analysis (Tobias et al.)

To validate that modules learned from scRNA-seq by ICAnet can be applied on bulk RNA-seq dataset to predict survival, we used the gene expression profiles of 562 patients treated in the German AMLCG 1999 trial as training dataset (37). We downloaded the expression and survival information from GEO (GSE37642) and used log-transformation to transform the dataset. Further, we kept genes shared between this dataset and the AML expression dataset from TCGA (The Cancer Genome Atlas) (38). Finally, we ran GSVA (39) to calculate module activity.

Mouse whole testis scRNA-seq

All the animal procedures were carried out in accordance with the guide for the Care and Use of Laboratory Animals and institutional guidelines in Nanjing Medical University. All mice were maintained in a specific environment with 12/12-h light/dark cycles, a suitable temperature about 22°C and a comfortable humidity, and allowed

free access to clean water and food. The animals used in this study were C57BL/6J mice. We digested male mice germ cells by the method described by Bellvé with minor modifications. Briefly, mice testes were harvested after euthanasia, washed twice in PBS and incubated in 10 ml DMEM (Gibco) containing collagenase (1 mg/ml) at 37 °C for 15 min with gentle shaking. The dispersed seminiferous tubules were washed twice with DMEM and collected by centrifugation at 300 g for 5 min at 4°C. Tubules were further digested in Trypsin (0.25%, Gibco) containing DNase (1 mg/ml, Qiagen) at 37 °C for 5 min. After stopping the enzyme digestion with 10% Fetal Bovine Serum (Gibco) and then collecting cells by centrifugation at 300 g for 5 min at 4°C, washed once with 10 ml DMEM, and then filtered through a 40 μm Nylon Cell Strainer to gain a single cell suspensions. Cell concentration was counted twice using Cellometer Mimi (Nexcelom Bioscience). Cell suspension was mixed with Trypan Blue (Life Technology) with the volume ratio of 1:1. Then cells' viability was counted under an introverted microscope. Subsequently, samples with cell viability higher than 80% were loaded into Single Cell A Chip (10X Genomics, Chromium) with a certain concentration that 5000 cells were expected to be recovered. After that, single cell Gel Bead-In-EMusions (GEMs) were generated in Chromium Single Cell Controller (10X Genomics). Library construction were performed followed the user guide of Single Cell 3' Reagent Kits v2. Quality of cDNA and sequencing libraries were tested using Qubit 3.0 fluorometer and Agilent bioanalyzer 2100. Finally, libraries were sequenced in Illumina Novaseq. Raw sequencing data were converted to fastq format using cellranger mkfastq (10x Genomics, v3.0.0). scRNA-seq reads were aligned to GRCh37 reference genome and quantified using cellranger count (10x Genomics, v.3.0.0). We filtered out low quality cells based on the plots of the distribution of genes detected and UMIs for both samples. Based on these plots, we filtered out the cells with ≤ 500 detected genes or those with ≥ 10% of transcripts corresponding to mitochondria-encoded genes. We also filtered out genes that were detected in ≤ 1% of all samples. Further, we used 'SCTransform' normalization (4) to normalize the gene expression to remove unwanted effects induced by mitochondrial mapping percentage.

2. Supplementary Figures

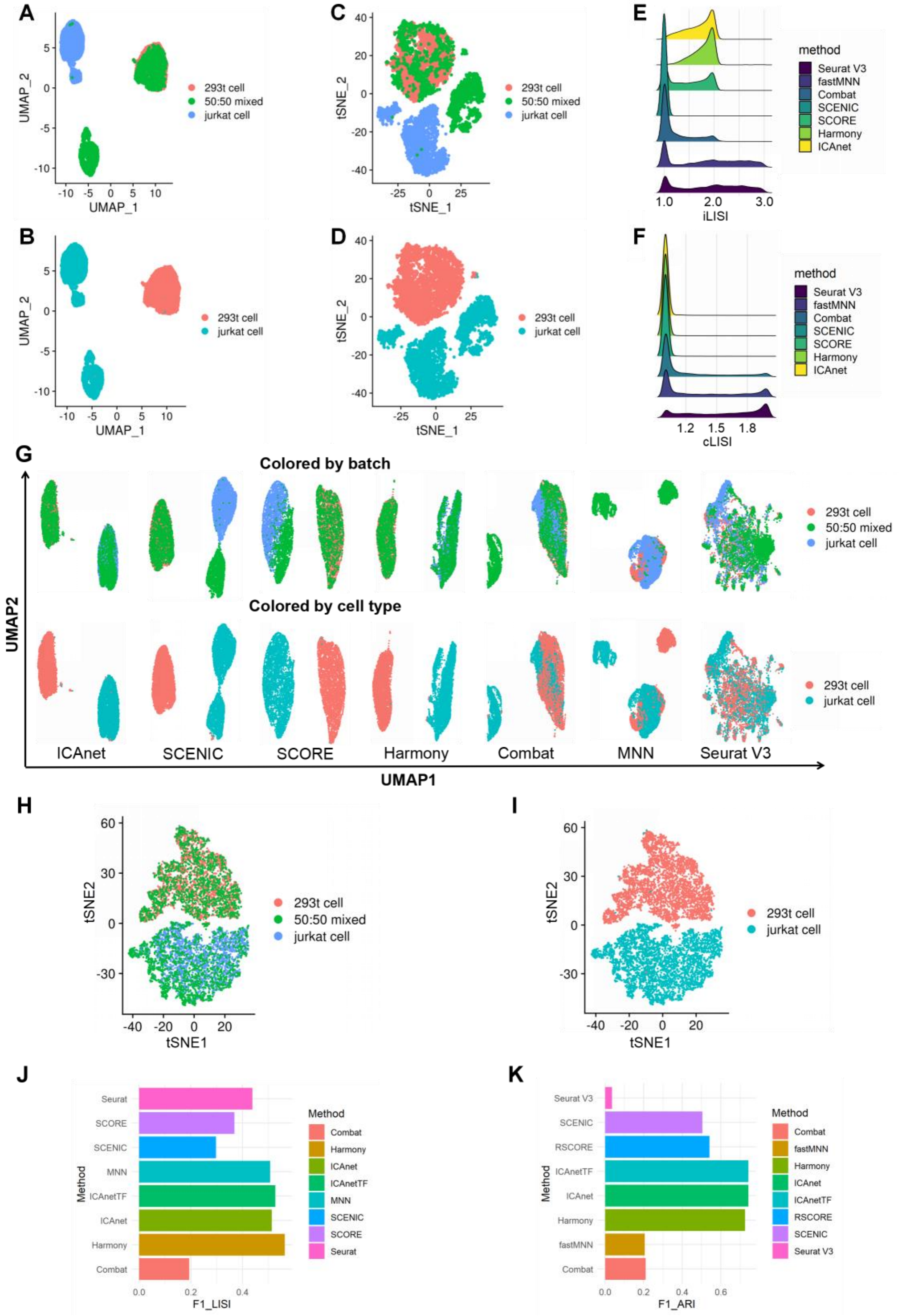

Figure S1. Performance evaluation of batch-effect correction methods on cell line datasets.

A-D. UMAP (A,B) and t-SNE (C,D) plots of three single cell datasets (**DS1** in

Supplementary Table S1) before batch effect correction. Colored by batch label (panel A and C, pure 293T, pure Jurkat and 50:50 mixed) or cell type label (panel B and D, 293T and Jurkat) annotated by original authors.
E-F. Score distribution of iLISI (E) and cLISI (F) for seven methods using cell line datasets mentioned above.
G. UMAP visualization of seven batch-effect correction approaches regarding batch label and cell type label defined by original authors.
H-I. t-SNE visualization of ICAnetTF regarding batch label (H) and cell type label (I) defined by original authors.
J-K. Barplots of LISI's F1 score (J) and ARI's F1 score (K) for eight methods.

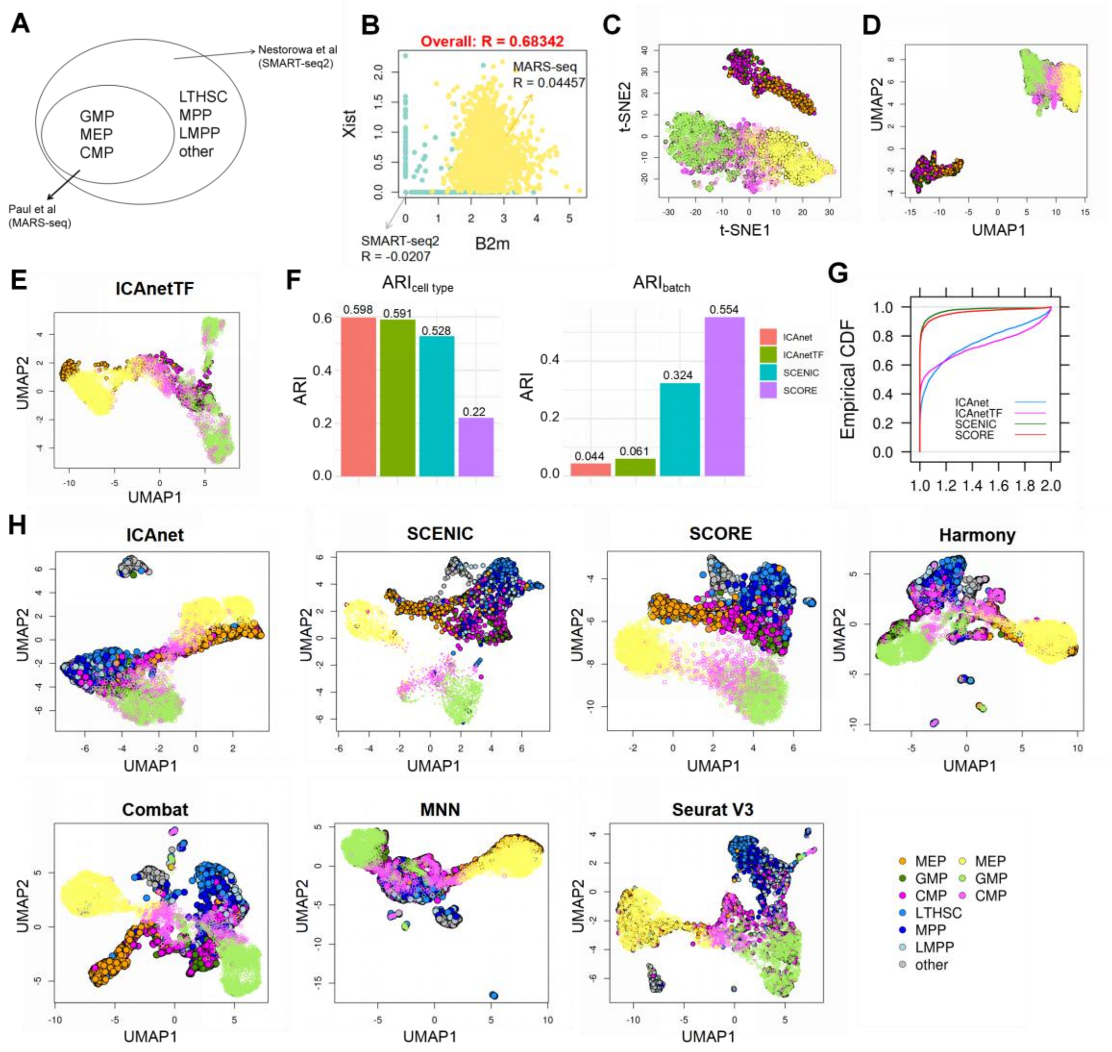

Figure S2. Integration performance evaluation of different integration methods using mouse hematopoietic scRNA-seq datasets.

A. Venn diagram shows the shared and specific cell types for the two scRNA-seq datasets (**DS2** in **Supplementary Table S1**).

B. Dot plot of expression value (log-normalization) of two genes in two scRNA-seq datasets. Aquamarine and yellow dots denote cells from SMART-Seq2 and MARS-Seq, respectively. R: Pearson correlation coefficient.

C-D. t-SNE (c) and UMAP (d) plots of the raw processing pipeline without batch effect correction. The light and dark colored dots represent cells from MARS-Seq (Paul et al) and SMART-Seq2 (Nestorowa et al), respectively.

E. t-SNE projection plots of the TF-regulon matrix generated by ICAnet.

F. Assessment of cell type accuracy (left), batch correction efficiency (middle) and combined performance (right) reflected by $ARI_{cell\ type}$, ARI_{batch}, respectively. A larger $ARI_{Cell\ type}$ value means better performance, while a smaller value ARI_{batch} denotes better batch effect correction.

G. Assessment of batch mixing through iLISI.

H. UMAP plots of seven batch effect correction methods. The light and dark colored dots represent cells from MARS-Seq (Paul et al) and SMART-Seq2 (Nestorowa et al), respectively. Cell types in the two datasets were indicated at bottom right.

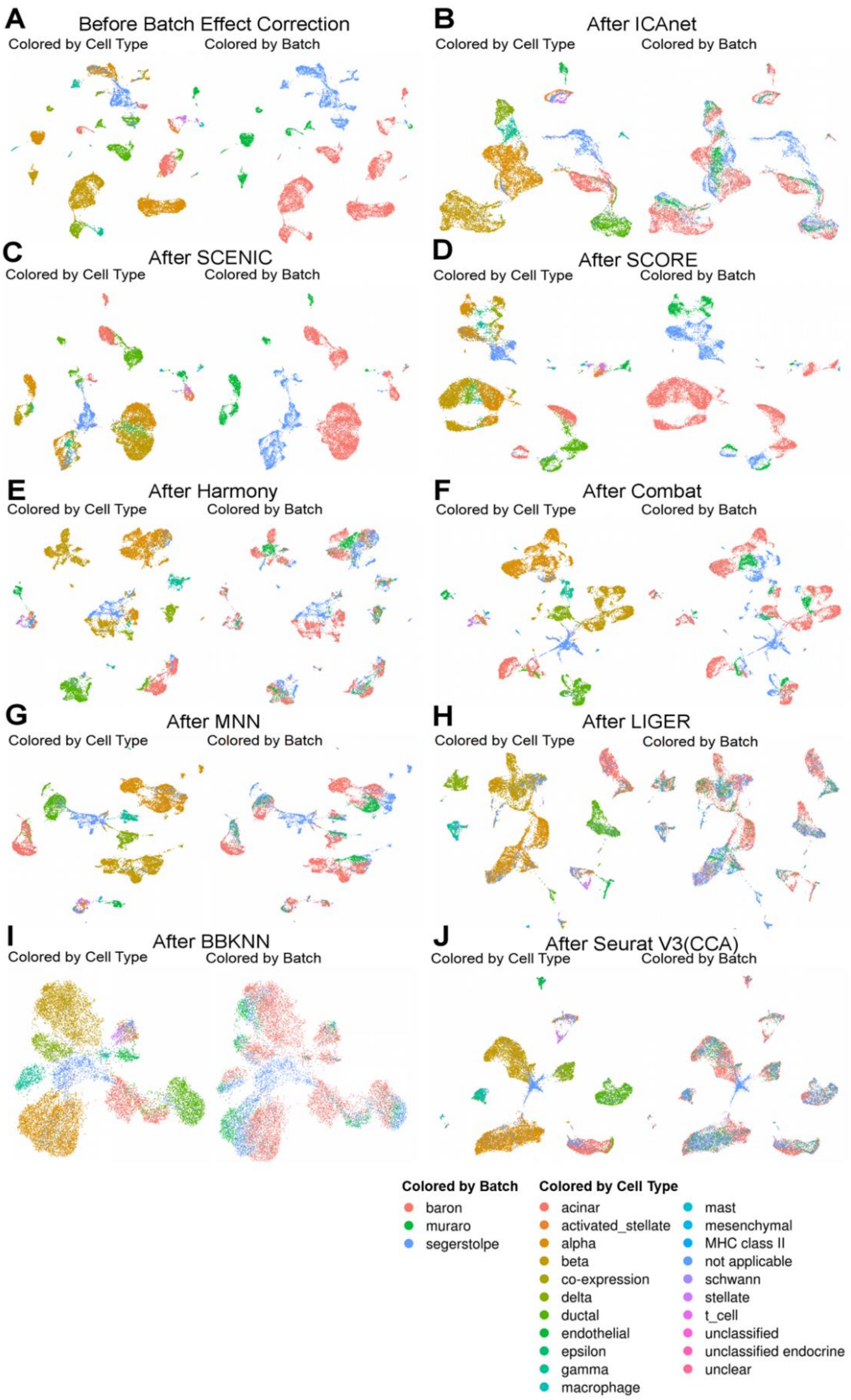

Figure S3. **UMAP plots for cell clustering results of raw workflow (A) and nine integration methods (B-J) for scRNA-seq datasets of pancreas islet.** Both cell type and batch information by the original authors were shown by different colors. The dataset **DS5** in **Supplementary Table S1** was used in this analysis.

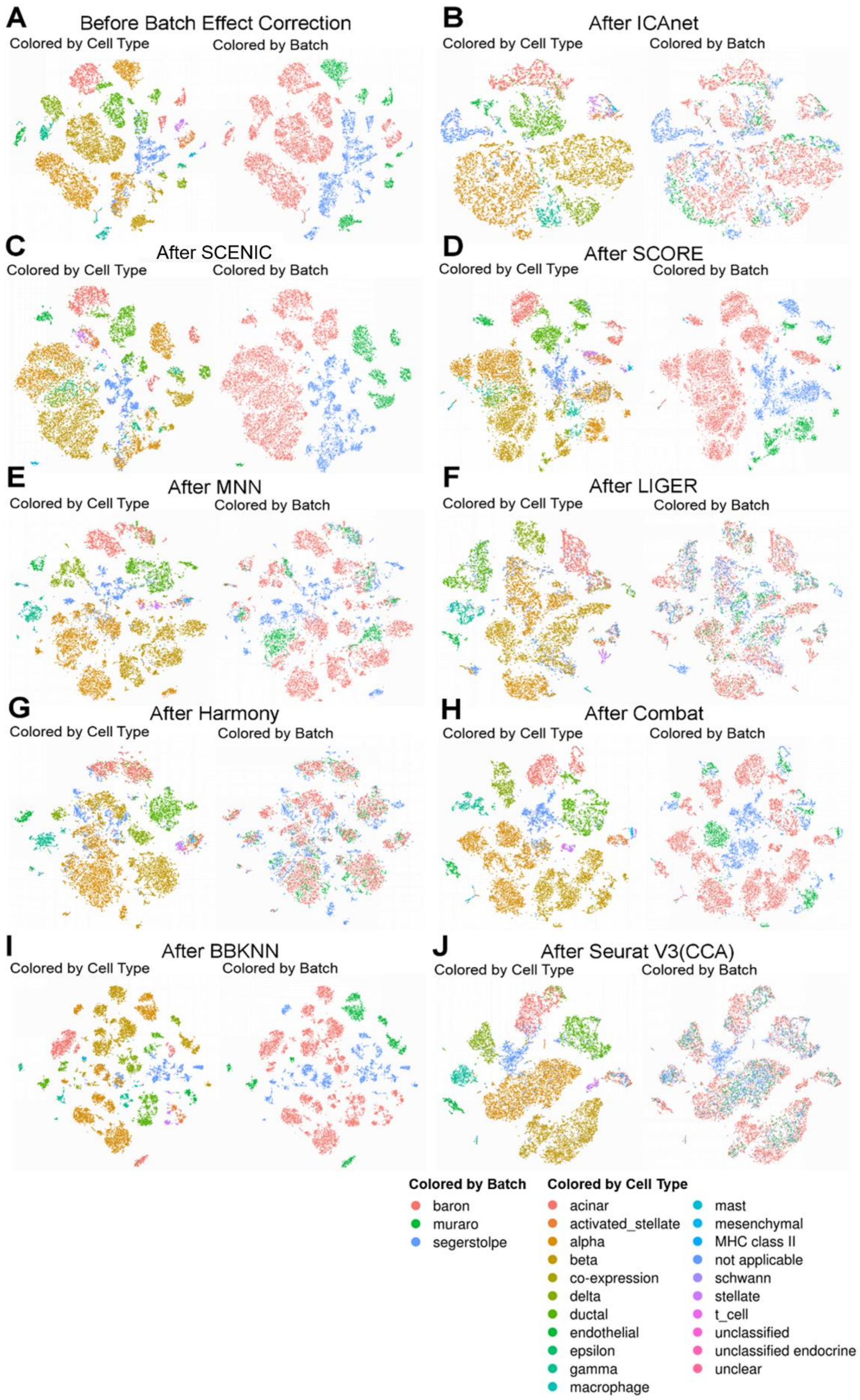

Figure S4. t-SNE plots of raw workflow (A) and nine integration methods (B-J) for scRNA-seq datasets of pancreas islet. Both cell type and batch information by the original authors were shown by different colors. The dataset **DS5** in **Supplementary Table S1** was used in this analysis.

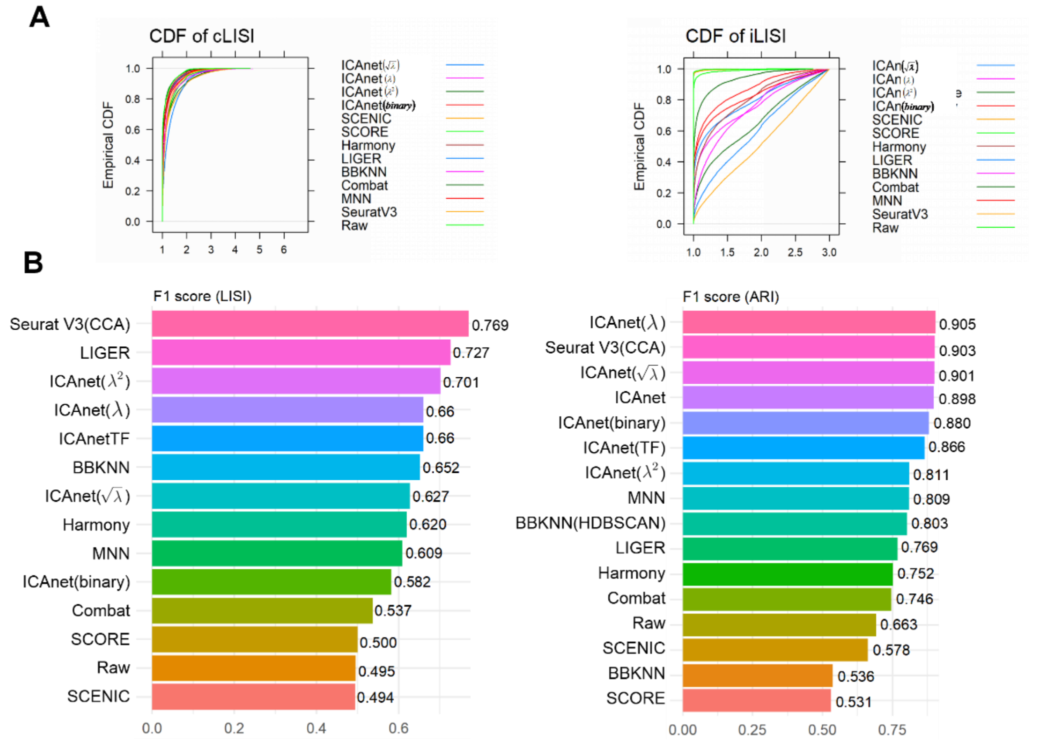

Figure S5. Benchmarking the performance of ten methods using pancreas islet scRNA-seq dataset.

A. Cumulative distribution function (CDF) curves of cLISI (left) and iLISI (right) by ten methods for pancreas scRNA-seq datasets (DS5 in **Supplementary Table S1**). The value of cLISI and iLISI was calculated in UMAP space.

B. F1 score of LISI (left) and ARI (right) by ten methods for pancreas scRNA-seq datasets.

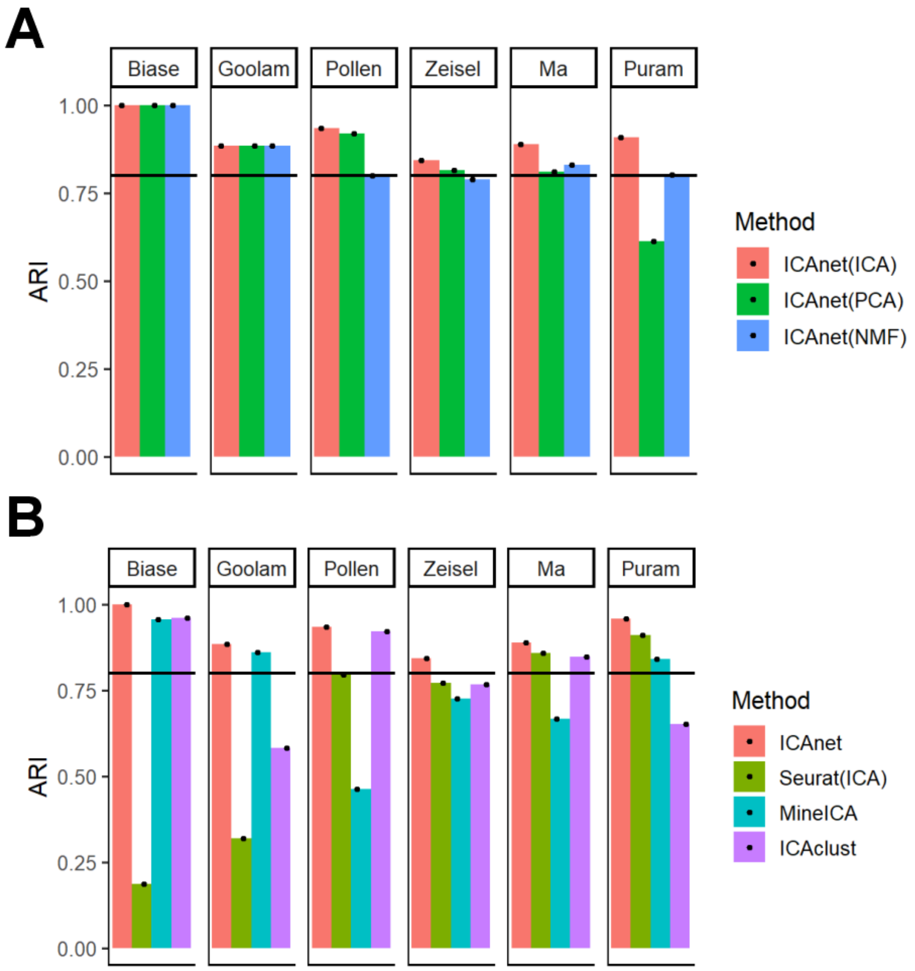

Figure S6. Clustering performance comparison between ICAnet and other matrix decomposition or ICA-based methods.

A. Each dataset is labelled by the name of first author in the original publication. Three matrix decomposition algorithms (including ICA, PCA (SVD), and NMF) were used for the comparison. ARI value is shown in the Y axis. PCA and SVD are similar strategies, so we used PCA(SVD) as one condition.

B. Each dataset is labelled by the name of first author of the original publication. ICA-based tools including ICAnet, Seurat (ICA), MineICA and ICAclust were used for the comparison. ARI value representing the clustering performance is shown in the Y axis.

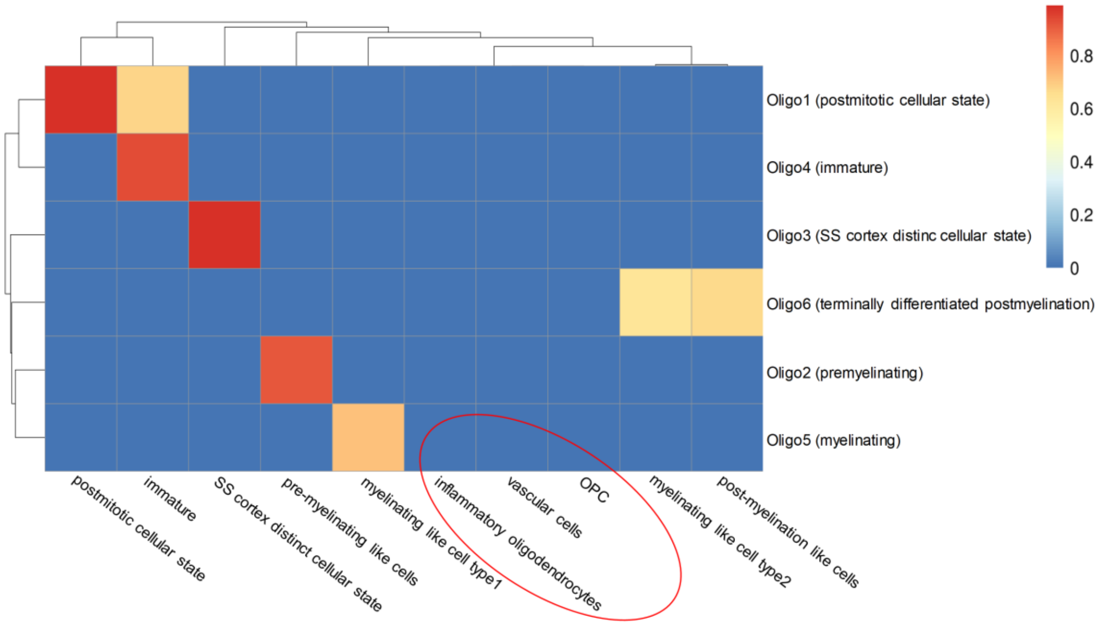

Figure S7. Label association between our cell type annotation and author-annotated cell type annotation.

Label association between our annotation and the original cell type annotation given by Zeisel et al. Each label correlation is measured through PCC (Pearson correlation coefficients). PCC values that less than 0.6 were set to 0. Cell types marked by a red circle denote our newly-inferred cell type (or states). The mouse brain scRNA-seq dataset (**DS4** in **Supplementary Table S1**) was used in this analysis.

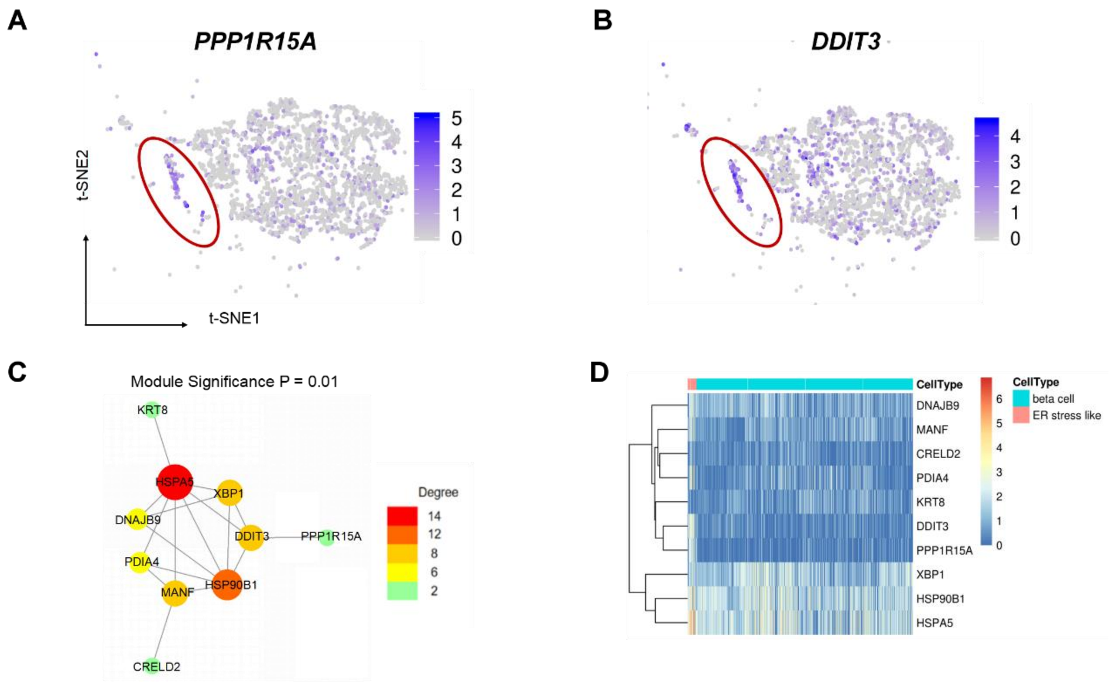

Figure S8. ICAnet identifies a rare cell type featured with ER stress associated module in pancreatic islet dataset.

A-B. t-SNE plots showing a small proportion of beta cells (red circle) highly expressing two ER stress marker genes (*PPP1R15A* and *DDIT3*). The color bar denotes the expression levels (log_2CP10K). Pancreas islet dataset (**DS5** in **Supplementary Table S1**) was used in this analysis.

C. A representative subnetwork (module) specific to ER stress beta cell. Each node represents a gene, and the node size and color density represent node degree.

D. Heatmap showing expression level of 10 genes in ER-stress related module (panel C) in both normal and ER stress beta cells.

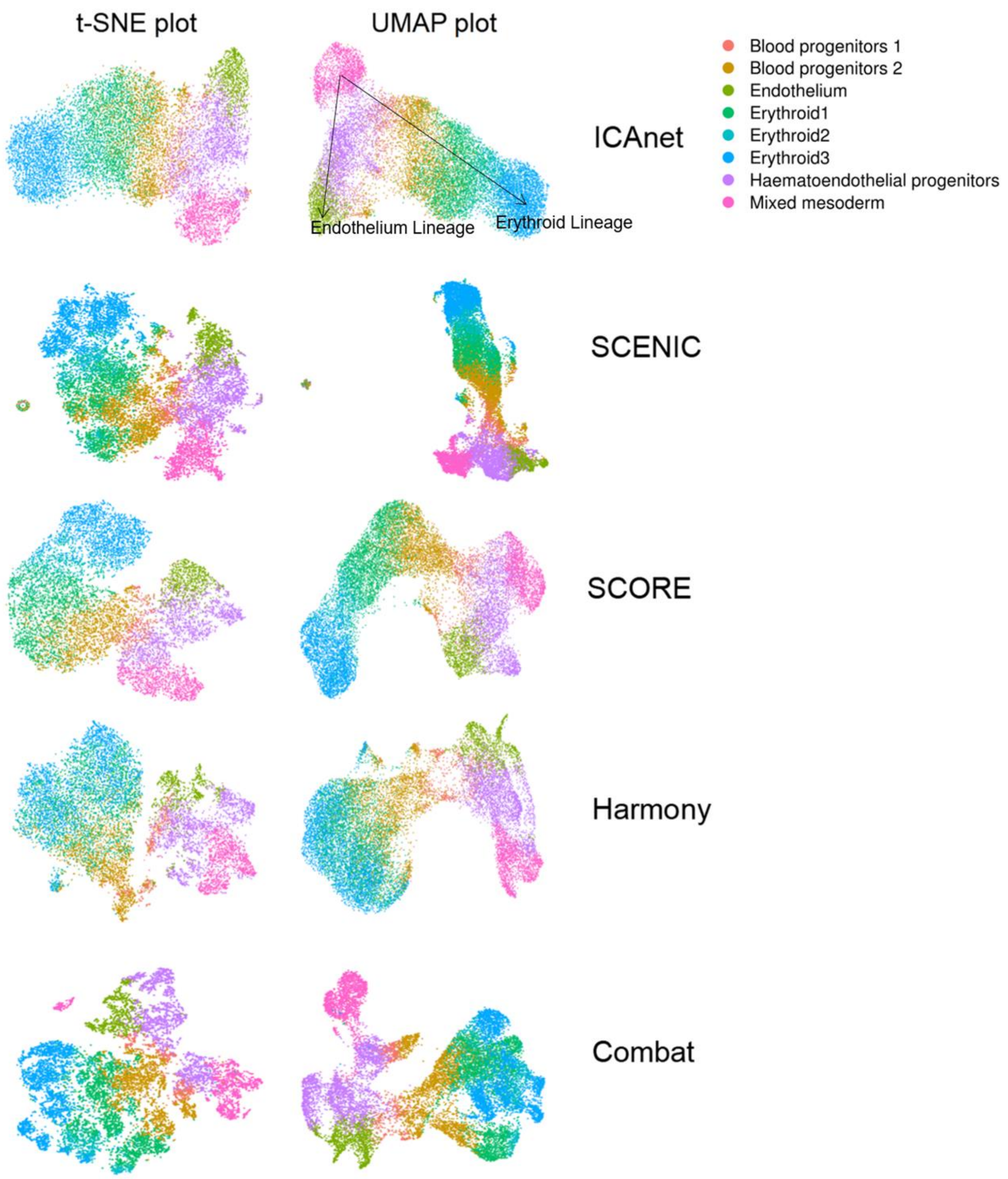

Figure S9. t-SNE and UMAP plots of five integration methods on mouse hematopoiesis development single cell dataset. Each dot is colored according to the cell type annotated by original authors. Mouse hematopoiesis development scRNA-seq dataset (**DS6** in **Supplementary Table S1**) was used in this analysis.

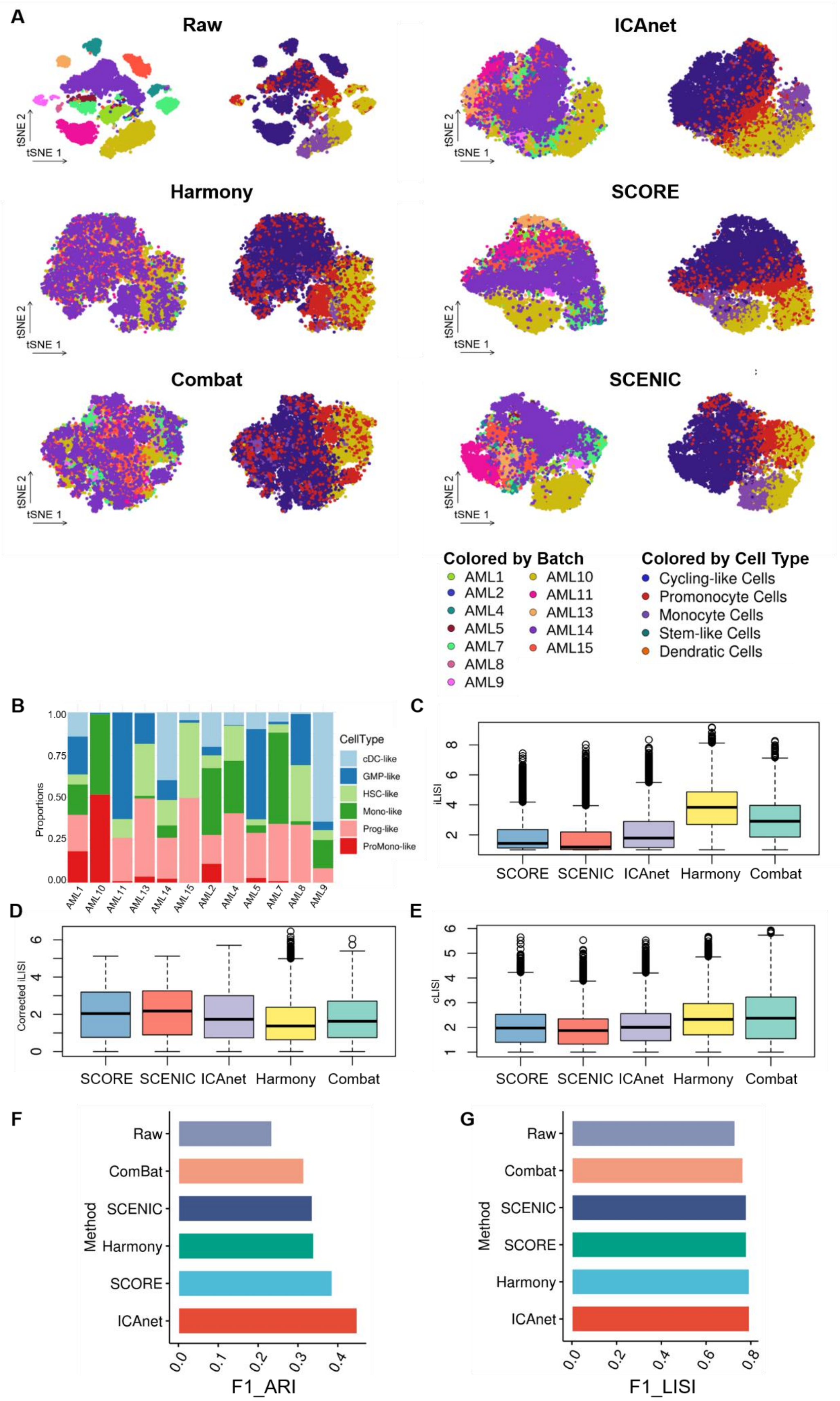

Figure S10. Performance evaluation of raw workflow and five data integration methods on AML scRNA-seq datasets.

A. For each method, the left t-SNE plot is colored according to the batch ID, and the right one is colored according to the cell type ID annotated by the original publication. AML scRNA-seq dataset (DS7 in **Supplementary Table S1**) was used in this analysis.
B. The proportion of cell type in each patient (donor) based on information from the original publication.
C. The raw iLISI value calculated to measure the local batch mixing around each cell.
D. The corrected iLISI value for each method. See Section 2 of Supplementary Note for more details.
E. The cLISI value calculated to measure the local cell type purity around each cell.
F-G. Barplots of ARI's F1 score (F) and LISI's F1 score (G) for six methods.

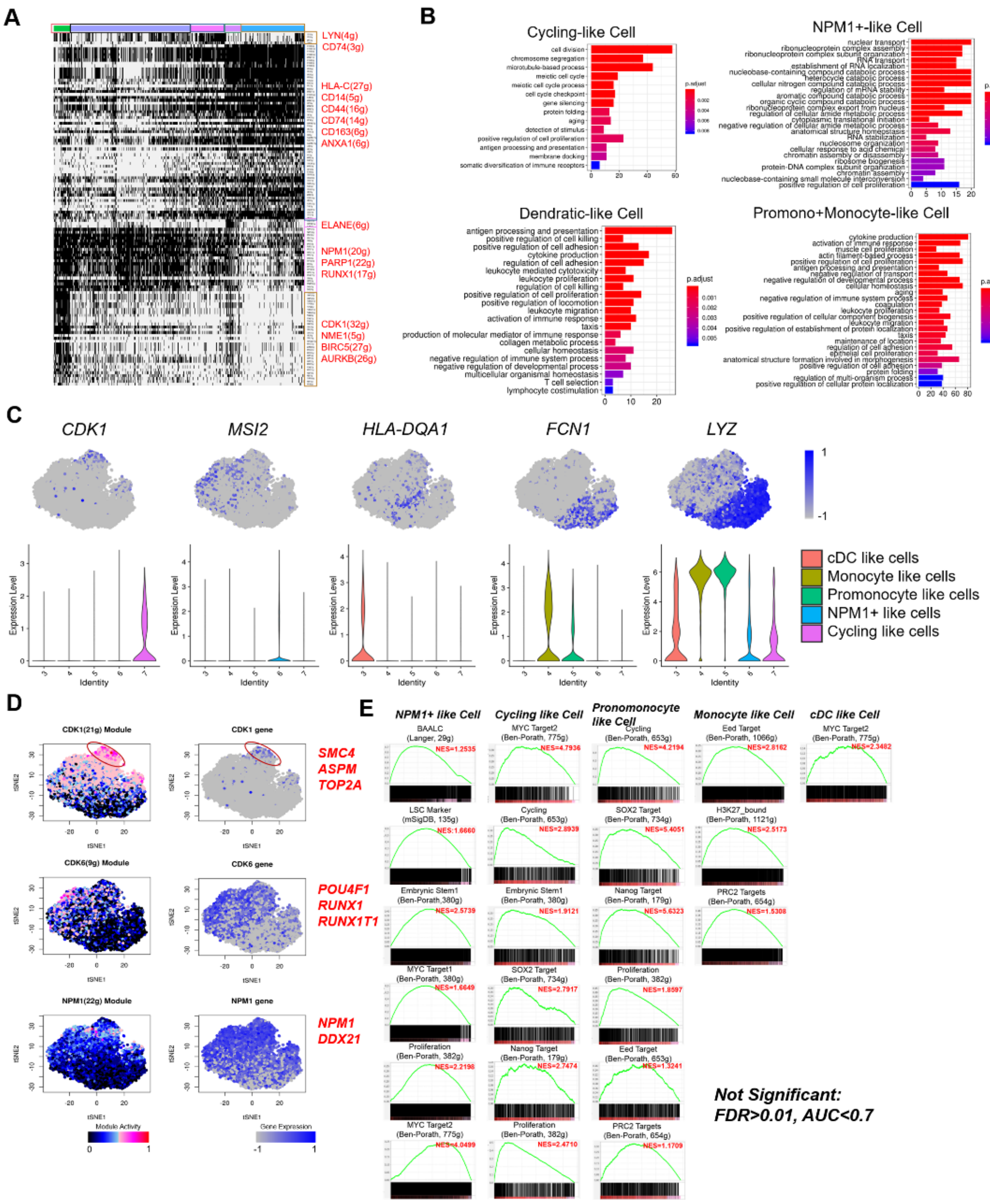

Figure S11. ICAnet reveals AML malignant cell heterogeneity.

A. Binary regulon activity matrix of modules derived from AML single cell RNA-seq datasets. Modules with hub genes associated with AML risk were indicated on the right by the hub gene.

B. Enriched GO terms (by clusterProfile) for genes in the cell type associated modules. Bar length denotes the number of genes and the color key denotes the adjusted p-values.

C. tSNE plots (up) displaying the expression level of representative marker genes across all the AML cells. Each dot represents a cell, and the degree of color reflects the expression level of indicated genes. Violin plots at the bottom showed the normalized expression levels of representative marker genes in each cell type.

D. t-SNE visualization of important modules associated with cell type specific functions in AML. The left column is the t-SNE plots displaying the module activity of important

modules across all AML cells. For each of cell, the lighter of the dot, the higher of the module activity in corresponding cell. The right column is the t-SNE plots displaying the gene expression value of hub genes of corresponding modules. The darker of the blue dot, the higher of the gene expression value in corresponding cell.

E. Gene set enrichment analysis (GSEA) on five cell states with 14 curated published gene sets. In the GSEA, the ranking (X axis) is based on the gene set activity acquired through AUCell. Only GSEA plots with genes significantly enriched in specific cell states are shown (FDR < 0.01, AUC < 0.7). BAALC: AML risk gene BAALC associated gene set.

3. Supplementary Tables

Supplementary Table S1. Detailed information of all public available scRNA-seq datasets used in this study.

Dataset		Label (Cell or author information)	Description	Number of batches	Number of cells	Total cell number	Accession number	scRNA-seq technology	Potential sources of batch effect	Purpose
DS1	DS1-1	293T	Cell line	1	2758	8783	NA	10X	Same technology, Different mixture source	Test if ICAnet could correct batch effect of different scRNA-seq dataset from different cell types
	DS1-2	Jurkat	Cell line	1	3175		NA	10X		
	DS1-3	50%:50% Jurkat and 293T cell mixture	Cell line	1	2850		NA	10X		
DS2	DS2-1	Paul et al.	Mouse haematopoietic and progenitor cells	1	1920	4649	GSE72857	SMART-seq2	Different technology, Same tissue/cell	Test if ICAnet could correct batch effects of scRNA-seq datasets from different library types
	DS2-2	Nestorowa et al.	Mouse haematopoietic and progenitor cells	1	2729		GSE81682	MARS-seq		
DS3	DS3-1	Biase et al.	Mouse embryonic cells	1	56	56	GSE57249	SMART-seq2	NA (Not applicable)	Test the clustering performance of ICAnet
	DS3-2	Goolam et al.	Mouse embryonic cells	1	124	124	E-MATB-3321	SMART-seq2		
	DS3-3	Pollen et al.	Mouse cerebral cortex cells	1	301	301	SRP041736	SMARTer		
	DS3-4	Ma et al.	Liver cancer cells	1	5115	5115	GSE125449	droplet		
	DS3-5	Puram et al.	Head and neck cancer cells	1	5902	5902	GSE103322	inDrop		
DS4	DS4-1	Zeisel et al.	Mouse cortex and hippocampus dataset	1	3005	3005	GSE60361	STRT/C1	NA (Not applicable)	This dataset is used to test i) the clustering performance of ICAnet; ii) whether ICAnet has robustness to the sparse dataset; iii) whether ICAnet has robustness to the changed number of cells
DS5	DS5-1	Muraro et al.	Human pancreas islets cells	4	2126	14209	GSE84133	CEL-seq	Different technology, Same tissue	Test if ICAnet could correct batch effect of scRNA-seq datasets from multiple batches (≥3)
	DS5-2	Baron et al.	Human pancreas islets cells	1	8569		GSE85241	inDrop		
	DS5-3	Segerstolpe et al.	Human pancreas islets cells	1	3514		E-MTAB-5061	Smart-seq2		
DS6	DS6-1	Pijuan-Sala et al.	Mouse hematopoiesis time-course dataset	31	15022	15022	NA	10X	Same Technology, Different tissue/cell	Test if ICAnet could correct batch effect of time-course scRNA-seq datasets
DS7	DS7-1	Peter et al.	Human acute myeloid leukemia dataset	12	11641	11641	GSE116256	Seq-Well	Same Technology, Different tissue/cell	Test if ICAnet could correct batch effect of tumor scRNA-seq dataset from different patients

Table S2. Cell type and its featured hub genes and related GO terms in mouse brain scRNA-seq dataset.

Cell type	Hub Genes	GO term
oligodendrocytes progenitor cells	*Ptprc*	glutamate metabolic process, oligodendrocyte differentiation
post-mitotic cellular state	*Plxnb3, Acsl3*	developmental cell growth, small GTPase mediated signal transduction
immature	*Arpc5*	regulation of cell, morphogensis involved in differentiation
pre-myelinating like cells	*Sox10, Vapa*	regulation of cellular component size, gliogenesis, oligodendrocyte differentiation
myelinating like cell type1	*S100b, Anxa5*	xenobiotic catabolic process, detoxification
myelinating like cell type2	*Rbm25*	neuron transmitter secretion, cerebellar cortex morphogensis
post-myelinating like cell	*Slc8a2, Synj1*	dendritic spine, organization, regulated exocytosis
inflammatory oligodendrocyte	*Tyrobp, Cxcl1*	inflammatory response, leukocyte activation, myeloid leukocyte activation
SS cortex distinct cells	*Magt1, Serpinb1a*	ribosome biogenesis, rRNA processing
vascular cells	*Myh11*	blood vessel morphogenesis, cell-matrix adhesion, response to growth factor

Table S3. The multivariable Cox regression analysis with six prognosis variables.

Method	Coefficient (coef)	Standard Error (coef)	Z score	P value
**Module**	3.2147	0.5039	2.32	0.02
**HSC.R**	0.7813	0.1581	-1.56	0.119
**LSC.R**	1.1225	0.1891	0.61	0.541
**LSC17**	0.9798	0.4574	-0.04	0.964
**Marcucci**	1.3722	0.459	0.69	0.491
**Li**	1.1802	0.0718	2.31	0.021

Likelihood ratio test = 17.98 on 6 df (degree of freedom), p = 0.006, n = 151, number of events = 94.

References:

1. Teschendorff, A.E., Zhuang, J. and Widschwendter, M. (2011) Independent surrogate variable analysis to deconvolve confounding factors in large-scale microarray profiling studies. *Bioinformatics*, **27**, 1496-1505.
2. Plerou, V., Gopikrishnan, P., Rosenow, B., Amaral, L.A.N., Guhr, T. and Stanley, H.E. (2002) Random matrix approach to cross correlations in financial data. *Phys Rev E*, **65**.
3. Kiselev, V.Y., Kirschner, K., Schaub, M.T., Andrews, T., Yiu, A., Chandra, T., Natarajan, K.N., Reik, W., Barahona, M., Green, A.R. *et al.* (2017) SC3: consensus clustering of single-cell RNA-seq data. *Nat Methods*, **14**, 483-+.
4. Hafemeister, C. and Satija, R. (2019) Normalization and variance stabilization of single-cell RNA-seq data using regularized negative binomial regression. *Genome Biology*, **20**.
5. Aparicio, L., Bordyuh, M., Blumberg, A.J. and Rabadan, R. (2020) A Random Matrix Theory Approach to Denoise Single-Cell Data. *Patterns*, **1**, 100035.
6. van Galen, P., Hovestadt, V., Wadsworth, M.H., Hughes, T.K., Griffin, G.K., Battaglia, S., Verga, J.A., Stephansky, J., Pastika, T.J., Story, J.L. *et al.* (2019) Single-Cell RNA-Seq Reveals AML Hierarchies Relevant to Disease Progression and Immunity. *Cell*, **176**, 1265-+.
7. Butler, A., Hoffman, P., Smibert, P., Papalexi, E. and Satija, R. (2018) Integrating single-cell transcriptomic data across different conditions, technologies, and species. *Nat Biotechnol*, **36**, 411-+.
8. Zeisel, A., Munoz-Manchado, A.B., Codeluppi, S., Lonnerberg, P., La Manno, G., Jureus, A., Marques, S., Munguba, H., He, L.Q., Betsholtz, C. *et al.* (2015) Cell types in the mouse cortex and hippocampus revealed by single-cell RNA-seq. *Science*, **347**, 1138-1142.
9. Dong, F., Hao, S., Zhang, S., Zhu, C., Cheng, H., Yang, Z., Hamey, F.K., Wang, X., Gao, A., Wang, F. *et al.* (2020) Differentiation of transplanted haematopoietic stem cells tracked by single-cell transcriptomic analysis. *Nature Cell Biology*, **22**, 630-639.
10. Li, H., Courtois, E.T., Sengupta, D., Tan, Y., Chen, K.H., Goh, J.J.L., Kong, S.L., Chua, C., Hon, L.K., Tan, W.S. *et al.* (2017) Reference component analysis of single-cell transcriptomes elucidates cellular heterogeneity in human colorectal tumors. *Nature Genetics*, **49**, 708-718.
11. Teschendorff, A.E. and Enver, T. (2017) Single-cell entropy for accurate estimation of differentiation potency from a cell's transcriptome. *Nature communications*, **8**, 15599-15599.
12. Alanis-Lobato, G., Möllmann, J.S., Schaefer, M.H. and Andrade-Navarro, M.A. (2020) MIPPIE: the mouse integrated protein–protein interaction reference. *Database*, **2020**.
13. Oughtred, R., Stark, C., Breitkreutz, B.-J., Rust, J., Boucher, L., Chang, C., Kolas, N., O'Donnell, L., Leung, G., McAdam, R. *et al.* (2019) The BioGRID interaction database: 2019 update. *Nucleic Acids Research*, **47**, D529-D541.
14. Szklarczyk, D., Franceschini, A., Wyder, S., Forslund, K., Heller, D., Huerta-Cepas, J., Simonovic, M., Roth, A., Santos, A., Tsafou, K.P. *et al.* (2015) STRING v10: protein-protein interaction networks, integrated over the tree of life. *Nucleic acids research*, **43**, D447-D452.
15. Teschendorff, A.E., Journee, M., Absil, P.A., Sepulchre, R. and Caldas, C. (2007) Elucidating the altered transcriptional programs in breast cancer using independent component analysis. *Plos Comput Biol*, **3**, 1539-1554.

16. Wu, M., Li, X., Kwoh, C.-K. and Ng, S.-K. (2009) A core-attachment based method to detect protein complexes in PPI networks. *Bmc Bioinformatics*, **10**, 169.
17. Liberzon, A., Subramanian, A., Pinchback, R., Thorvaldsdóttir, H., Tamayo, P. and Mesirov, J.P. (2011) Molecular signatures database (MSigDB) 3.0. *Bioinformatics*, **27**, 1739-1740.
18. Croft, D., O'Kelly, G., Wu, G., Haw, R., Gillespie, M., Matthews, L., Caudy, M., Garapati, P., Gopinath, G., Jassal, B. *et al.* (2011) Reactome: a database of reactions, pathways and biological processes. *Nucleic Acids Research*, **39**, D691-D697.
19. Ximerakis, M., Lipnick, S.L., Innes, B.T., Simmons, S.K., Adiconis, X., Dionne, D., Mayweather, B.A., Nguyen, L., Niziolek, Z., Ozek, C. *et al.* (2019) Single-cell transcriptomic profiling of the aging mouse brain. *Nature Neuroscience*, **22**, 1696-1708.
20. Lopes, P.C. and König, B. (2020) Wild mice with different social network sizes vary in brain gene expression. *Bmc Genomics*, **21**, 506.
21. Aibar, S., Gonzalez-Blas, C.B., Moerman, T., Van, A.H.T., Imrichova, H., Hulselmans, G., Rambow, F., Marine, J.C., Geurts, P., Aerts, J. *et al.* (2017) SCENIC: single-cell regulatory network inference and clustering. *Nat Methods*, **14**, 1083-+.
22. Chen, Y., Zheng, Y.X., Gao, Y., Lin, Z., Yang, S.M., Wang, T.T., Wang, Q., Xie, N.N., Hua, R., Liu, M.X. *et al.* (2018) Single-cell RNA-seq uncovers dynamic processes and critical regulators in mouse spermatogenesis. *Cell Research*, **28**, 879-896.
23. Qiu, X.J., Mao, Q., Tang, Y., Wang, L., Chawla, R., Pliner, H.A. and Trapnell, C. (2017) Reversed graph embedding resolves complex single-cell trajectories. *Nat Methods*, **14**, 979-+.
24. Green, C.D., Ma, Q.Y., Manske, G.L., Shami, A.N., Zheng, X.N., Marini, S., Moritz, L., Sultan, C., Gurczynski, S.J., Moore, B.B. *et al.* (2018) A Comprehensive Roadmap of Murine Spermatogenesis Defined by Single-Cell RNA-Seq. *Dev Cell*, **46**, 651-+.
25. Nestorowa, S., Hamey, F.K., Sala, B.P., Diamanti, E., Shepherd, M., Laurenti, E., Wilson, N.K., Kent, D.G. and Gottgens, B. (2016) A single-cell resolution map of mouse hematopoietic stem and progenitor cell differentiation. *Blood*, **128**, E20-E31.
26. Paul, F., Arkin, Y., Giladi, A., Jaitin, D.A., Kenigsberg, E., Keren-Shaul, H., Winter, D., Lara-Astiaso, D., Gury, M., Weiner, A. *et al.* (2015) Transcriptional Heterogeneity and Lineage Commitment in Myeloid Progenitors. *Cell*, **163**, 1663-1677.
27. Marques, S., Zeisel, A., Codeluppi, S., van Bruggen, D., Mendanha Falcão, A., Xiao, L., Li, H., Häring, M., Hochgerner, H., Romanov, R.A. *et al.* (2016) Oligodendrocyte heterogeneity in the mouse juvenile and adult central nervous system. *Science (New York, N.Y.)*, **352**, 1326-1329.
28. Jakel, S., Agirre, E., Falcao, A.M., Van Bruggen, D., Lee, K.W., Knuesel, I., Malhotra, D., Ffrench-Constant, C., Williams, A. and Castelo-Branco, G. (2019) Altered human oligodendrocyte heterogeneity in multiple sclerosis. *Nature*, **566**, 543-+.
29. Goolam, M., Scialdone, A., Graham, S.J.L., Macaulay, I.C., Jedrusik, A., Hupalowska, A., Voet, T., Marioni, J.C. and Zernicka-Goetz, M. (2016) Heterogeneity in Oct4 and Sox2 Targets Biases Cell Fate in 4-Cell Mouse Embryos. *Cell*, **165**, 61-74.
30. Pollen, A.A., Nowakowski, T.J., Shuga, J., Wang, X.H., Leyrat, A.A., Lui, J.H., Li, N.Z., Szpankowski, L., Fowler, B., Chen, P.L. *et al.* (2014) Low-coverage single-cell mRNA sequencing reveals cellular heterogeneity and activated signaling pathways in developing cerebral cortex. *Nat Biotechnol*, **32**, 1053-+.

31. Ma, L.C., Hernandez, M.O., Zhao, Y.M., Mehta, M., Tran, B., Kelly, M., Rae, Z., Hernandez, J.M., Davis, J.L., Martin, S.P. *et al.* (2019) Tumor Cell Biodiversity Drives Microenvironmental Reprogramming in Liver Cancer. *Cancer Cell*, **36**, 418-+.
32. Puram, S.V., Tirosh, I., Parikh, A.S., Patel, A.P., Yizhak, K., Gillespie, S., Rodman, C., Luo, C.L., Mroz, E.A., Emerick, K.S. *et al.* (2017) Single-Cell Transcriptomic Analysis of Primary and Metastatic Tumor Ecosystems in Head and Neck Cancer. *Cell*, **171**, 1611-+.
33. Muraro, M.J., Dharmadhikari, G., Grun, D., Groen, N., Dielen, T., Jansen, E., van Gurp, L., Engelse, M.A., Carlotti, F., de Koning, E.J.P. *et al.* (2016) A Single-Cell Transcriptome Atlas of the Human Pancreas. *Cell Syst*, **3**, 385-+.
34. Baron, M., Veres, A., Wolock, S.L., Faust, A.L., Gaujoux, R., Vetere, A., Ryu, J.H., Wagner, B.K., Shen-Orr, S.S., Klein, A.M. *et al.* (2016) A Single-Cell Transcriptomic Map of the Human and Mouse Pancreas Reveals Inter- and Intra-cell Population Structure. *Cell Syst*, **3**, 346-+.
35. Segerstolpe, A., Palasantza, A., Eliasson, P., Andersson, E.M., Andreasson, A.C., Sun, X.Y., Picelli, S., Sabirsh, A., Clausen, M., Bjursell, M.K. *et al.* (2016) Single-Cell Transcriptome Profiling of Human Pancreatic Islets in Health and Type 2 Diabetes. *Cell Metab*, **24**, 593-607.
36. Pijuan-Sala, B., Griffiths, J.A., Guibentif, C., Hiscock, T.W., Jawaid, W., Calero-Nieto, F.J., Mulas, C., Ibarra-Soria, X., Tyser, R.C.V., Ho, D.L.L. *et al.* (2019) A single-cell molecular map of mouse gastrulation and early organogenesis. *Nature*, **566**, 490-+.
37. Li, Z.J., Herold, T., He, C.J., Valk, P.J.M., Chen, P., Jurinovic, V., Mansmann, U., Radmacher, M.D., Maharry, K.S., Sun, M. *et al.* (2013) Identification of a 24-Gene Prognostic Signature That Improves the European LeukemiaNet Risk Classification of Acute Myeloid Leukemia: An International Collaborative Study. *J Clin Oncol*, **31**, 1172-1181.
38. Welsh, K.J., Nedelcu, E., Wahed, A., Bai, Y., Dasgupta, A. and Nguyen, A. (2015) Bioinformatics Analysis to Determine Prognostic Mutations of 72 de novo Acute Myeloid Leukemia Cases from the Cancer Genome Atlas (TCGA) with 23 Most Common Mutations and no Abnormal Cytogenetics. *Ann Clin Lab Sci*, **45**, 515-521.
39. Hanzelmann, S., Castelo, R. and Guinney, J. (2013) GSVA: gene set variation analysis for microarray and RNA-Seq data. *Bmc Bioinformatics*, **14**.
